# Characterization of Large Copy Number Variation in Mexican Type 2 Diabetes subjects

**DOI:** 10.1038/s41598-017-17361-7

**Published:** 2017-12-06

**Authors:** Iván de Jesús Ascencio-Montiel, Dalila Pinto, Esteban J. Parra, Adán Valladares-Salgado, Miguel Cruz, Stephen W. Scherer

**Affiliations:** 1 Instituto Mexicano del Seguro Social, Coordinación de Vigilancia Epidemiológica, Mier y Pesado 120, Col. del Valle, Benito Juárez, 03100 Mexico City, Mexico; 2 Departments of Psychiatry, Genetics and Genomic Sciences, The Mindich Child Health & Development Institute, Seaver Autism Center, Institute for Genomics and Multiscale Biology, at the Icahn School of Medicine at Mount Sinai, New York, 1470 Madison Avenue, S8-115, New York, NY 10029 USA; 3 Department of Anthropology, University of Toronto at Mississauga, 3359 Mississauga Road, room 352, Health Sciences Complex, Mississauga, ON L5L 1C6 Canada; 4 Unidad de Investigación Médica en Bioquímica, Hospital de Especialidades, Centro Médico Nacional Siglo XXI, Instituto Mexicano del Seguro Social, Av. Cuauhtémoc 330, Col. del Doctores, 06720 Mexico City, Mexico; 5 The Centre for Applied Genomics. The Hospital for Sick Children. Peter Gilgan Centre for Research and Learning, 686 Bay Street, Room 139800, Toronto, Ontario, M5G 0A4 Canada; 6 McLaughlin Centre and Department of Molecular Genetics, University of Toronto, 686 Bay Street, 13th Floor, Toronto, Ontario, M5G 0A4 Canada

## Abstract

The effect of Copy Number Variants (CNVs) on Type 2 Diabetes (T2D) remains little explored. The present study characterized large rare CNVs in 686 T2D and 194 non-T2D subjects of Mexican ancestry genotyped using the Affymetrix Genome-Wide Human SNP array 5.0. Rare CNVs with ≥ 100 kb length were identified using a stringent strategy based on merging CNVs calls generated using Birdsuit, iPattern and PennCNV algorithms. We applied three different strategies to evaluate the distribution of CNVs in the T2D and non-T2D samples: 1) Burden analysis, 2) Identification of CNVs in loci previously associated to T2D, and 3) Identification of CNVs observed only in the T2D group. In the CNV burden analysis, the T2D group showed a higher proportion of CNVs, and also a higher proportion of CNVs overlapping at least one gene than the non T2D group. Five of the six loci previously associated with T2D had duplications or deletions in the T2D sample, but not the non-T2D sample. A gene-set analysis including genes with CNVs observed only in the T2D group highlighted gene-sets related with sensory perception (olfactory receptors, OR) and phenylpyruvate tautomerase/dopachrome isomerase activity (*MIF* and *DDT* genes).

## Introduction

Type 2 Diabetes (T2D) is a complex metabolic disorder that constitutes a worldwide public health problem^1^. The evidence of a strong hereditary component in T2D has led to numerous efforts to study the genetic factors underlying the disease, mainly through genome-wide association studies (GWAS) focused on common variants^2–6^.

Copy Number Variants (CNVs), defined as DNA deletions or duplications above 1 kb in length, represent a significant proportion of human genetic variation and have been linked to many complex human diseases, but their implication in T2D remains little explored. In particular, large CNVs may encompass many genes and/or regulatory sequences and could be of particular relevance for T2D because of their potential pathological effects^7^.

The current Mexican population is an admixed population with a genetic background derived from European, Native American and to lesser extent, West African populations^8^. Mexico has one of the highest prevalences of T2D in the world^9^. In this study, we characterized large rare CNVs in T2D and non-T2D individuals (controls) of Mexican ancestry that were genotyped with the Affymetrix 5.0 array^10^. We then applied three different strategies in order to evaluate the distribution of CNVs in the T2D and control samples: 1) Burden analysis, 2) Identification of CNVs in loci previously associated to T2D, and 3) Identification of CNVs observed only in the T2D group.

## Results

### Subjects

A total of 686 T2D and 194 non-T2D control subjects were analyzed. As outlined in Table 1, the T2D group had more females and showed higher body mass index (BMI) and triglycerides but lower Multidimensional Scaling (MDS) ancestry vector 1 values than the control group (e.g. lower European ancestry). No differences were observed in relation to CNV size and deletion/duplication distribution among comparison groups [Supplementary Figure 4].

**Table 1:** General characteristics of the study subjects and of the rare CNVs in the T2D and control groups.

**Characteristic**	**T2D group**	**Control group**	**P value**
N	686	194	
**General characteristics**			
Male/female sex	205/481	89/105	<0.001*
Age, years	49 ± 12	50 ± 7	0.086
BMI, kg/m^2^	29.2 ± 6.4	27.4 ± 4.3	<0.001*
Total cholesterol, mg/dL	210.5 ± 57	205.5 ± 50	0.119
Triglycerides, mg/dL	197.5 ± 133	133 ± 63	<0.001*
MDS1 ancestry vector	−0.0047 ± 0.0291	0.0072 ± 0.0355	<0.001*
MDS2 ancestry vector	0.0018 ± 0.0067	0.0018 ± 0.0072	0.653
MDS3 ancestry vector	0.0009 ± 0.0055	0.0001 ± 0.0061	0.094
**Rare CNVs**			
Number of Rare CNVs	526	131	
Deletions	189 (35.9)	37 (28.2)	
Duplications	337 (64.1)	94 (71.8)	0.097
CNVs size in Mb	214.5 ± 230.3	208.2 ± 245.9	0.759
Deletions	171.3 ± 164.8	156.6 ± 87.4	0.335
Duplications	241.3 ± 255	254.1 ± 354.1	0.543
**Genic Rare CNVs**			
Number of Genic Rare CNVs	358	83	
Deletions	86 (24)	17 (20.5)	
Duplications	272 (76)	66 (79.5)	0.492
CNVs size in Mb	233.7 ± 245.3	257.7 ± 351.1	0.282
Deletions	181.7 ± 219.2	160.5 ± 78	0.552
Duplications	246.1 ± 244.6	311.6 ± 354.9	0.119

Data presented as counts (percentage) or median ± interquartile range.

P value between T2D and control groups by X^2^ and Mann–Whitney U test (numerical variables were not normally distributed).

T2D: type 2 diabetes; MDS1: Multidimensional scaling; BMI: Body Mass Index (kg/m^2^) CNVs: Copy number variations. *P < 0.05.

### Rare CNV burden analysis

**Figure 1:**
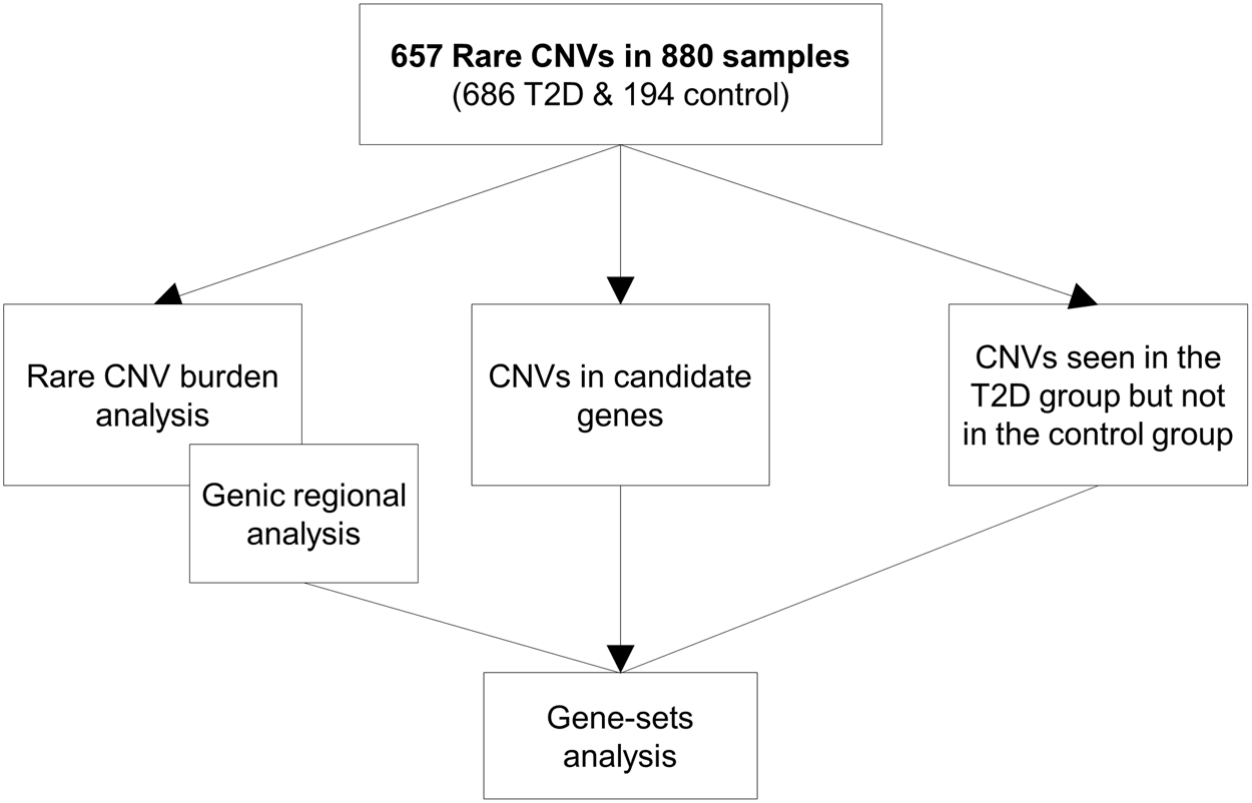
Analysis flowchart of the study’s aims.

In the first comparative analysis [Fig. 1], the rare CNV burden analysis, the T2D group showed a higher CNV deletion rate (0.25 vs 0.19, corrected p-value = 0.029) and a higher proportion of rare genic CNVs (e.g. CNVs overlapping with at least one gene) than the control group (0.42 vs 0.31, corrected p-value = 0.044). We did not observe significant differences in CNV rate, genic CNV rate, CNV sample proportion, total CNV size and genic CNV enrichment between the T2D and control groups [Table 2]. In the genic CNV regional analysis, *ZNF718* CNVs were found inversely associated with T2D (p = 0.048 for Duplication + Deletions). However, it is important to note that all the CNVs identified in this gene were present only in the control group [Table 3].

**Table 2:** Rare CNV burden analysis with respect to CNV size and CNV rate.

**Rare CNV burden analyzes**	**T2D group**	**Control group**	**Both groups**	**T2D/ Control ratio**	**Raw P-value**	**Corrected P-value**
**CNV rate** ^1^						
Deletions	0.25	0.19	0.26	1.30	0.127	0.029*
Duplications	0.51	0.48	0.49	1.04	0.437	0.475
Deletions + Duplications	0.75	0.68	0.75	1.11	0.21	0.116
**CNV sample proportion** ^2^						
Deletions	0.22	0.18	0.22	1.20	0.187	0.097
Duplications	0.40	0.37	0.37	1.08	0.319	0.533
Deletions + Duplications	0.55	0.48	0.51	1.13	0.112	0.269
**Total CNV size** ^3^						
Deletions	291.9	271.1	299.30	1.08	0.386	0.258
Duplications	419.2	484.0	444.90	0.87	0.816	0.85
Deletions + Duplications	420.2	466.5	449.40	0.90	0.811	0.699
**Average CNV size** ^4^						
Deletions	258.70	263.90	259.10	0.98	0.499	0.574
Duplications	341.70	360.50	336.60	0.95	0.642	0.811
Deletions + Duplications	306.20	338.00	311.10	0.91	0.779	0.871
**Genic CNV rate** ^5^						
Deletions	0.32	0.16	0.27	2.03	0.079	0.073
Duplications	1.25	1.20	1.14	1.04	0.467	0.641
Deletions + Duplications	1.57	1.36	1.41	1.16	0.304	0.424
**Genic CNV proportion** ^**6**^						
Deletions	0.11	0.08	0.11	1.38	0.176	0.116
Duplications	0.34	0.26	0.30	1.32	0.065	0.107
Deletions + Duplications	0.42	0.31	0.37	1.33	0.027*	0.044*
**Genic CNV enrichment** ^**7**^						
Deletions	0.005	0.005	0.005	0.94	0.51	0.545
Duplications	0.009	0.008	0.009	1.23	0.195	0.176
Deletions + Duplications	0.008	0.007	0.007	1.17	0.231	0.298

^1^Number of rare CNVs per sample.

^2^Proportion of samples with one or more rare CNVs.

^3^Total rare CNV kb length spanned.

^4^Average rare CNV size.

^5^Number of regions/genes spanned by rare CNVs.

^6^Proportion of rare CNVs with at least one gene.

^7^Number of regions/genes per total rare CNVs kb size.

T2D: type 2 diabetes; CNVs: Copy number variations; kb kilobases.

P-values were estimated by permutation (one-sided, 10,000 permutations). Corrected P-values were adjusted by sex, body mass index, triglycerides and ancestry (MDS1). *P < 0.05.

**Table 3:** CNV regions associated with T2D and rare CNVs diabetes candidate genes.

**Genic region**	**Location**	**Coordinates** ^**1**^	**Size (Mb)**	**DGV frequency (%)** ^**2**^	**CNV type**	**T2D group (n = 686)**	**Control group (n = 194)**
**CNV regions associated with T2D**							
*ZNF718*	4p16.3	chr4:43,276-146,490	103.2	0.0252	Deletions	0 (0)	1 (0.52)
					Duplications	0 (0)	1 (0.52)
					Deletions + Duplications*	0 (0)	2 (1.03)
**Rare CNVs in diabetes candidate genes**							
*AMY2B*	1p21.1	chr1:103,898,844-103,923,672	24.8	0.0486	Duplication	1 (0.15)	0 (0)
*LPP*	3q28	chr3:189,413,414-190,080,135	666.7	0.0026	Duplication	1 (0.15)	0 (0)
*ARL15*	5p15.2	chr5:53,216,370-53,642,160	425.8	0.0430	Duplication	0 (0)	1 (0.52)
*HFE*	6p22.1	chr6:26,195,487-26,203,448	8.0	0.0087	Deletion	1 (0.15)	0 (0)
*CDKAL1*	6p22.3	chr6:20,642,666-21,339,743	697.1	0.0000	Deletion	1 (0.15)	0 (0)
*RASGRP1*	15q14	chr15:36,567,593-36,644,299	76.7	0.0339	Duplication	1 (0.15)	0 (0)

^1^Coordinates according NCBI v36, hg18.

^2^Frequency according to the Database of Genomic Variants (DGV).

T2D: type 2 diabetes; CNVs: Copy number variations; kb kilobases; NA: Not available.

P-values were estimated by exact Fisher test. Corrected P-values by sex, body mass index, triglycerides and ancestry (MDS1) were not available. *P < 0.05.

### CNVs in candidate genes

We evaluated if rare CNVs in our samples overlapped with a list of 129 *loci* previously described as being implicated in T2D [Supplementary Table 3]. In the T2D group, we found duplications in the genes *AMY2B*, *LPP* and *RASGRP1* and deletions in the *HFE* and *CDKAL1* genes. In the control group, we found a deletion in the *ARL15* gene [Table 3].

### CNVs seen in the T2D group but not in the control group

We observed a total of 76 genic CNVs in the T2D group that were not seen in the control group and also had frequencies below 0.2% in the Database of Genomic Variants (DGV). These CNVs overlapped with 123 genes, 59 of which are listed in the Online Mendelian Inheritance in Man database (www.omim.org ) [Supplementary Table 4].

### Gene-sets analysis

**Figure 2:**
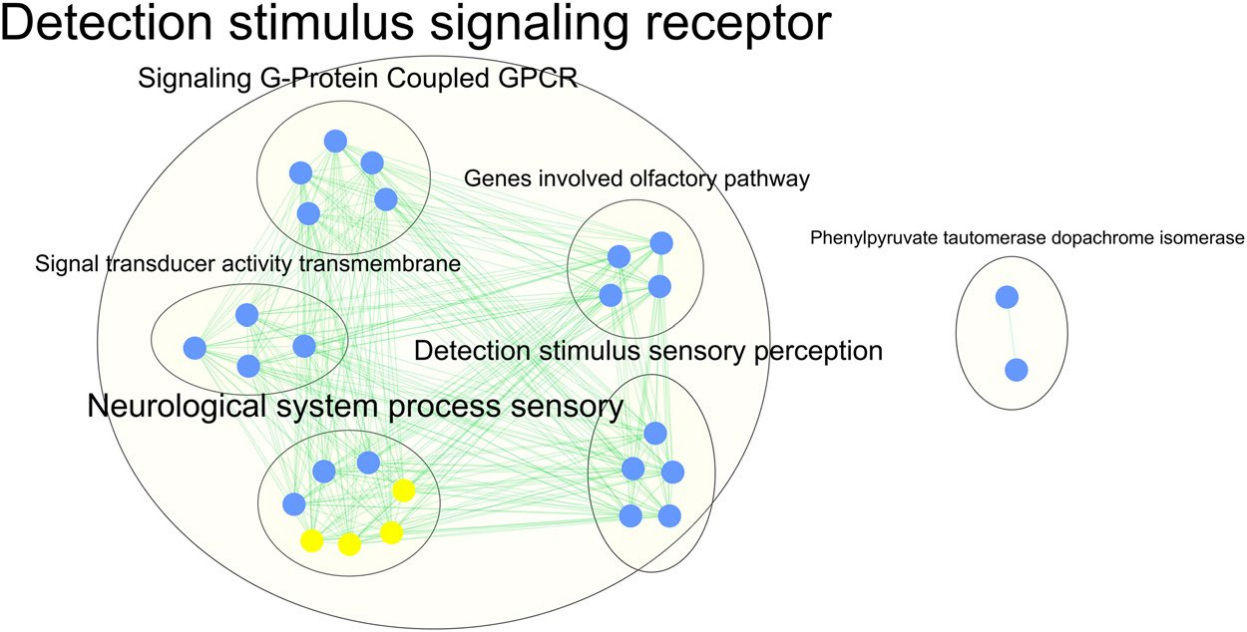
Results of the gene-set analysis. The map shows a network of gene sets (nodes) related by mutual overlap (edges). A total of 130 genes derived from the genic regional analysis, the analysis of CNVs in candidate genes and the analysis of genic CNVs seen in the T2D group but not in the control group were used as input for the gene-set analysis, which identified 27 gene-sets. Blue circles corresponded to gene-sets with duplications and yellow circles represented gene-sets with duplications and deletions.

A total of 130 genes were included in the gene-set analysis (one gene identified in the genic regional analysis, 6 genes from the candidate gene analysis, and 123 genes from the analysis of CNVs that are present in the T2D group but not in the control group). The gene-set analysis identified 27 gene-sets. Most of the gene-sets related to sensory perception (olfactory receptors –OR-). Two of the gene-sets pointed to phenylpyruvate tautomerase/dopachrome isomerase activity. Duplications are the most common type of CNV observed in these gene-sets [Fig. 2, Supplementary Table 5].

## Discussion

T2D is a complex disorder caused by genetic and environmental factors. The high concordance between twins and the disparities in rates of development of T2D from children of parents with and without the diabetes show the influence of genetic factors in the occurrence the disease^11^. GWAS have emerged as the most successful methodology to discover genetic variants associated with complex diseases^4–6^.

CNVs represent a significant proportion of human genetic variation. It has been estimated that 79.55% of the genome contains CNVs (http://dgv.tcag.ca/dgv/app/home). In the last few years, there has been an increased interest in the evaluation of the contribution of rare CNVs to complex diseases^7,12–14^. This type of genomic variation could be relevant for disease risk due to dosage or disrupting effects. For example, genic duplications may create imbalance due to the excess of products of duplicated genes^7^.

It is possible to characterize CNVs using microarray data. In this study, we characterized large rare CNVs in 686 T2D and 194 control subjects of Mexican ancestry genotyped using the Affymetrix Genome-Wide Human SNP array 5.0. In our comparative analyses, we found that the T2D group showed a higher proportion of CNVs overlapping with at least one gene than the control group. In our genic CNV regional analysis, we observed that CNVs (Duplications + Deletions) in the *ZNF718* gene were associated to T2D status. However, all the duplications and deletions were observed in the control group, and this does not support a role of CNVs within this gene in the pathogenesis of T2D. We found CNVs in 6 *loci* previously associated with T2D (*AMY2B*, *LPP*, *RASGRP1*, *HFE*, *CDKAL1*, *ARL15)*. With the exception of the deletion found in the *ARL15* gene, all the other CNVs (duplications or deletions) were observed in the T2D group. Finally, we identified a list of 130 genes for further analysis, based on three different strategies: 1) Genic CNV regional analysis, 2) Identification of CNVs overlapping with previously reported T2D genes, and 3) Genic CNVs observed in the T2D but not in the control group. A gene-set analysis including these 130 genes identified 27 gene-sets. Most of the aforementioned gene-sets included OR genes. The remaining gene-sets included the genes *MIF* and *DDT*, which are involved in Dopachrome/phenylpyruvate isomerase activity.

Loss of smell has been associated with reductions in fat mass and insulin resistance^15^. However, it is important to note that the OR gene family is the largest vertebrate gene family and several studies have shown that OR loci are enriched in CNVs because they are often located in segmentally duplicated regions^16–20^. For example, in a high-resolution study using oligonucleotide tiling microarrays, Hasin *et al*.^16^ analyzed 851 OR gene and pseudogene loci, and reported the presence of CNVs in 93 OR genes and 153 pseudogenes. Our gene-set results have been primarily driven by the inclusion of genes with CNVs identified in the T2D group but absent in the control group. It is possible that the enrichment in OR genes observed in our analysis is the result of ORs being prone to the presence of CNVs, in combination with the unbalanced number of T2D and control samples in our study, instead of a functional effect of CNVs located within OR genes on T2D risk. Another gene-set identified in our analysis includes the gene *MIF*, which codes for the cytokine macrophage migration inhibitory factor that has been previously related with adipose tissue inflammatory processes and with T2D^21,22^. It would be important to carry out additional research to evaluate if CNVs affecting this gene may play a role in T2D risk.

Similarly to a recent study in Mexican Americans^23^, we found that duplications are on average larger in size than deletions. However, in contrast to Blackburn’s study, in which deletions were more frequent than duplications (deletion/duplication ratio between 1.56 to 2.75), in our study we observed a larger number of duplications than deletions (deletion/duplication ratio between 0.25 to 0.56). These differences may be due to the type of microarray assay, the CNV calling algorithms and/or the CNV length and selection protocols used in both studies. Our study also found that the T2D group had a higher CNV deletion rate in comparison with the control group. Our CNV deletion and duplication distribution was quite similar to that described in the T2D and control cohorts from the Welcome Trust Case-Control Consortium study^24^.

The contemporary Mexican population is the result of an admixture process that involved Native American, European and African populations^25–28^. The average ancestral contributions of this sample have been estimated as 60%, 35% and 5% respectively^25^. It is well known that the presence of population stratification can inflate the rate of false positives in association studies in admixed populations. One strategy to minimize this problem was to include ancestry proportions as covariates in the statistical analyses. In our study, we used the coordinates of the first MDS axis, which reflects the relative ancestral contributions from European and Native American populations. In addition to the MDS1 ancestry vector, the rare CNV burden analysis was also adjusted by sex, BMI, and triglycerides, given that significant differences for these variables were observed between the T2D and control groups in the initial comparative analysis. Unfortunately, due the small counts, the aforementioned adjustment was not possible neither for *ZNF718* gene nor for the diabetes candidate gene analysis.

The lack of detailed clinical information like blood glucose and hemoglobin A1c concentrations, the relatively high percentage of samples dropped during the QC protocol, the limited sample size and the unbalanced number of T2D subjects and controls are the main limitations of our study. With respect to the number of samples removed, Grassi^29^ reported that 12.8% of the samples genotyped with the Affymetrix Genome-Wide Human SNP array 5.0 were eliminated using only two quality control criteria (gender incompatibilities, and genotyping rate <90%). The higher percentage of samples removed in our study (32.8%) could be explained by the rigorous four-step quality control analysis used to maximize our confidence in the CNV calls.

Despite the limited sample size and the unbalanced number of T2D and control samples, in a post-hoc analysis, the power to detect overall genic CNV proportion differences and *ZNF718* gene differences among comparison groups were 0.799 and 0.646 respectively. However, the statistical power decrease to 0.483 when there was a CNV in a control subject but not in T2D and 0.003 when there was a CNV in a T2D subject but not in controls.

## Conclusions

In this study conducted in Mexico we found that the T2D group showed a higher proportion of CNVs than the control sample, and also a higher proportion of CNVs overlapping at least one gene than the control group. We found duplications or deletions in six genes that have been previously associated with type 2 diabetes. Finally, gene-sets related with sensory perception (olfactory receptors) and phenylpyruvate tautomerase/dopachrome isomerase activity were enriched in the T2D group.

## Methods

### Study design and population

As outlined below, we analyzed a total of 686 T2D and 194 non-T2D control subjects selected from the initial sample comprised 1,310 individuals (967 T2D subjects and 343 controls) that were previously included in a GWAS for T2D^10^. Briefly, the T2D sample included subjects previously diagnosed according to the American Diabetes Association (ADA) criteria^30^ recruited from Family Medicine Clinics located at the southern part of Mexico City. The control group included healthy blood donors without family history of T2D and with normal glucose tolerance tests, according to the ADA criteria, that were recruited from the Central Blood Bank of the National Medical Center “Siglo XXI”. Both groups were sampled between 2000 and 2005. Information on sex, age, BMI, total cholesterol and triglycerides was available for all the samples. Ancestry proportions were estimated using a genome-wide panel of 3,500 Ancestry Informative Markers (AIMs) with the program ADMIXMAP [http://homepages.ed.ac.uk/pmckeigu/admixmap/index.html] and also a MDS approach based on genome-wide data for six parental population samples (Nahua and Maya from Mexico, Andeans and Aymara from South America, Spanish, CEPH European and Yoruba) and the Mexico City sample.

### SNP genotyping

Genotyping was carried out at the Centre for Applied Genomics (Toronto, ON) using the Affymetrix Genome-Wide Human SNP array 5.0 (Affymetrix, Santa Clara, CA) and following standard protocols.

### Quality control analysis and CNV analysis

A four-step quality control (QC) procedure was applied to the initial set of 1,310 samples to ensure that ascertainment of CNVs was consistent between comparison groups. Rare CNVs with ≥100 kb length were identified using a stringent strategy based on merging CNVs calls generated using Birdsuite (version 1.5.3)^31^, iPattern^32,33^ and PennCNV^34^ algorithms. At the end of the QC analysis, the dataset comprised 880 individuals (686 T2D subjects and 194 control subjects) and 657 rare large CNVs. [Supplementary Information, Supplementary Figures 1–3 and Supplementary Tables 1 and 2].

### Comparative analysis of general characteristics

Comparisons of sex, age, BMI, total cholesterol, triglycerides, ancestry vectors, CNV size and deletion/duplications proportions between the T2D and control groups were carried out with the X^2^ and Mann–Whitney U tests.

### Rare CNV burden analysis

The CNV burden analysis was carried out using PLINK v 1.07 (http://zzz.bwh.harvard.edu/plink/), considering two categories: rare CNVs and genic rare CNVs (e.g. CNVs that overlap with at least one gene). The global CNV burden in the T2D group compared to the control group was tested using seven measures: 1) CNV rate (number of CNVs per sample), 2) CNV sample proportion (proportion of samples with one or more CNVs), 3) total CNV size, 4) average CNV size, 5) genic CNV rate (number of regions/genes spanned by rare CNVs), 6) genic CNV proportion (proportion of rare CNVs overlapping at least one gene) and 7) genic enrichment (number of regions/genes per total CNVs kb size). Statistical significance was evaluated using an adaptive permutation procedure for one-sided tests (i.e., hypothesizing that T2D subjects will show greater burden of rare CNVs than controls). For each of 10,000 permutations, samples were randomly reassigned either T2D or control status. Rare measures were also adjusted by significant variables in the comparative group analysis: sex, BMI, triglycerides and ancestry (coordinates of the first MDS axis, which explains the relative proportion of Native American and European ancestry in the individuals analyzed). CNVs were considered rare if they were found at a frequency of <1% in the Mexican control sample set (n = 194), and showed an overlap of less than 50% of their length with other CNVs or segmental duplications found at a frequency >1%. Rare CNVs were classified as genic based on RefSeq annotations (UCSC, v. March 2006, NCBI v36, hg18).

In a second comparative analysis of burden of rare CNVs (genic regional analysis), we tested for specific genic regions associated with T2D, using Fisher’s exact test to assess for differences between the T2D and the control groups.

### CNVs in candidate genes and CNVs seen in the T2D group but not in the control group

After the rare burden analysis and the genic regional analysis, we looked for CNVs present in a list of 129 *loci* previously described as being implicated in T2D [Supplementary Table 3], and we also looked for CNVs present in T2D subjects but not in the control group.

### Gene-sets analysis

A final analysis was carried out to find gene-sets that may be implicated in T2D. Here we considered the results of the genic regional analysis, the analysis of CNVs in candidate genes and the analysis of genic CNVs seen in the T2D group but not in the control group. Enrichment Map plugin v2.2.1 (http://baderlab.org/Software/EnrichmentMap) and Cytoscape software v3.5.1 (http://cytoscape.org/) were used to draw a map of the gene-sets network, in which the nodes represented the gene-sets and the edges corresponded to the overlap between sets.

### Ethics Statement

The research was approved by the National Ethical Committee of the Mexican Institute of Social Security (register number 2008-785-073) and by The Ethics Review Office at the University of Toronto. Written informed consent was obtained from each participant. All methods were performed in accordance with the ethical standards and regulations of the institutional research committees and national laws and with the 1964 Helsinki declaration and its later amendments.

### Data Availability Statement

The datasets analysed during the current study are not publicly available due to ethical concerns, but are available from the corresponding author on reasonable request.

## Electronic supplementary material

Supplementary Information
