# Supplementary material for: Characterization of Large Copy Number Variation in Mexican Type 2 Diabetes subjects
Source: Sci Rep. 2017 Dec 6;7:17105. doi: 10.1038/s41598-017-17361-7 (PMC5719030; doi:10.1038/s41598-017-17361-7)
Supplement: Supplementary file 1 — Supplementary Information [file 41598_2017_17361_MOESM1_ESM.doc]

**Supplementary Information**

**Title: "Characterization of Large Copy Number Variation in Mexican Type 2 Diabetes subjects"**

**Names of the authors**

Iván de Jesús Ascencio-Montiel*(1); Dalila Pinto (2); Esteban J. Parra (3); Adán Valladares-Salgado (4); Miguel Cruz (4) & Stephen W. Scherer (5,6)

Affiliations

(1) Instituto Mexicano del Seguro Social, Coordinación de Vigilancia Epidemiológica. Address: Mier y Pesado 120, Col. del Valle, Benito Juárez, 03100, Mexico City, Mexico.

(2) Seaver Autism Center, The Mindich Child Health & Development Institute, Icahn School of Medicine at Mount Sinai, New York. Address: 1470 Madison Avenue, S8-115. New York, NY 10029, USA

(3) Department of Anthropology, University of Toronto at Mississauga, Mississauga, Ontario, L5L 1C6, Canada. Address: 3359 Mississauga Road, room 352, Health Sciences Complex. Mississauga, ON L5L 1C6, Canadá.

(4) Unidad de Investigación Médica en Bioquímica, Hospital de Especialidades, Centro Médico Nacional Siglo XXI, Instituto Mexicano del Seguro Social. Address: Av. Cuauhtémoc 330, Col. del Doctores, 06720, Mexico City, Mexico.

(5) The Centre for Applied Genomics. The Hospital for Sick Children. Peter Gilgan Centre for Research and Learning. Address: 686 Bay Street, Room 139800. Toronto, Ontario M5G 0A4, Canada.

(6) McLaughlin Centre and Department of Molecular Genetics, University of Toronto. Address: 686 Bay Street, 13th Floor. Toronto, Ontario M5G 0A4, Canada

*Corresponding Author

ivan-ascencio@hotmail.com

Phone +(52) 55-5726-1700 ext 15754

**Quality control analysis and CNV analysis**

Prior to the statistical analysis, a four steps quality control (QC) analysis were applied to the initial set of 1,310 samples to ensure that ascertainment of CNVs was consistent between comparison groups [**Supplementary Figure 1**].

In the first three steps (basic exclusion QC criteria, sample batch level QC and sample level QC), 346 samples were eliminated: one sample was due to genotyping call rate < 95%, 238 due to array intensity standard deviation > 0.3, 105 due to standard deviation (SD) for Log R ratio > 0.3, one due to B Allele Frequency (BAF) drift > 0.01 and another one due to wave factor < -0.035 and > +0.035 [**Supplementary Figure 2** and **Supplementary Table 1**].

After those three first QC steps, CNV calling was carried out using the three following algorithms: Birdsuite (version 1.5.3) (Korn *et al.* 2008), iPattern (Pinto *et al.* 2010; Pinto *et al.* 2011) and PennCNV (Wang *et al.* 2007), getting 34,578, 45,342 and 45,854 CNVs calls, respectively in the remaining 964 samples. CNVs calls located at the Y chromosome and CNVs with < 5 probes and < 5kb length were excluded from analysis, this last criterion was applied due the high probability that a CNV with low size and detected by few probes, correspond to a false detection. [**Supplementary Table 2**].

Later, 76 samples were discarded if their number of CNVs were above the 95th percentile in any of the three algorithms (72, 45 and 53 CNVs for PennCNV, Birdsuite and iPattern respectively). After removing these outlier samples, the correlation of the CNV calls per sample between algorithms was evaluated, showing that iPattern and Birdsuite had the best correlation (Pearson correlation coefficient=0.397, p<0.001) [**Supplementary Figure 3**].

Based on the correlation results, a stringent dataset was contructed merging the CNV calls from Birdsuite and iPattern algorithms (the PennCNV program was used only for further confirmation) [**Supplementary Figure 1**]. So, the dataset at the end of the stringent dataset construction comprised 47,882 CNVs from 888 participants.

From this list (888 subjects and 47,883 stringent CNVs), we excluded CNVs identified by only one algorithm (Birdsuite or iPattern) (n=30,155), with <100kb length (n=13,406), located at immunoglobulin (n=203) or pseudoautosomal regions (n=11), classified as gain/loss, that is with discordant copy number in iPattern and Birdsuite (n=81), or located at within pericentromeric pq arms (n=3), after visual inspection. Once these exclusion criteria were applying, there were 4,023 CNVs.

After that, 8 samples were discarded because did not show the expectation to had at least one CNVs with size ≥ 100kb (6 T2D subjects and 2 control subjects). Also, common CNVs (n=3,255) and CNVs with segmental duplications overlapping >50% of its length (n=111) were eliminated from the analysis, in order to get a sampleset with rare and large CNVs. CNVs were considered rare if they were found at a frequency of <1% of the Mexican control sample set (n=194), and did not overlap with >50% of its length, a CNV found at a frequency >1% or segmental duplications. Rare genic CNVs were obtained using genic regions identified based on RefSeq annotations (UCSC, v. March 2006, NCBI v36, hg18).

At the end of all this QC steps, the dataset comprised 880 individuals (686 T2D subjects and 194 control subjects) and 657 rare large CNVs. [**Supplementary Table 3** and **Supplementary Figure 1**]. The results of MDS1, MDS2 and MDS3 ancestry vectors among included and exluded T2D and control subjects are sowh in **Supplementary Figure 5**].

For validation of CNVs, visual inspection was done using PennCNV program. Representative plots of CNV regions associated with T2D and rare CNVs diabetes candidate genes are shown in **Supplementary Figure 6**.

**Supplementary Table 1**: Quality control (QC) analysis steps for samples in T2D and control groups

| **QC Evaluation (Sample filters)** | **T2D group** | | **Control group** | | **Both groups** | |
| --- | --- | --- | --- | --- | --- | --- |
|  | **# Samples removed** | **# Samples after QC** | **# Samples removed** | **# Samples after QC** | **# Samples removed** | **# Samples after QC** |
| Number of samples | 0 | 967 | 0 | 343 | 0 | 1310 |
| **Basic exclusion QC criteria** |  |  |  |  |  |  |
| Genotyping call rate < 95% | 1 | 966 | 0 | 343 | 1 | 1309 |
| Intensity QC call rate < 86% | 0 | 966 | 0 | 343 | 0 | 1309 |
| **Sample batch level QC** |  |  |  |  |  |  |
| Standard deviation of intensity >0.3 | 172 | 794 | 66 | 277 | 238 | 1071 |
| **Sample level QC** |  |  |  |  |  |  |
| Standard deviation of Log R ratio >0.3 | 39 | 755 | 66 | 211 | 105 | 966 |
| Standard deviation of B Allele Frequency >0.1 | 0 | 755 | 0 | 211 | 0 | 966 |
| B Allele Frequency drift > 0.01 | 1 | 754 | 0 | 211 | 1 | 965 |
| Weave factor < -0.035 and > +0.035 | 1 | 753 | 0 | 211 | 1 | 964 |
| **CNV detection sample level QC** |  |  |  |  |  |  |
| Number of calls ≥5kb and ≥5 probes obtained by  iPattern, PennCNV and Birdsuite algorithms >p95* | 61 | 692 | 15 | 196 | 76 | 888 |
| Without stringent calls ≥ 100kb | 6 | 686 | 2 | 194 | 8 | 880 |
| **Final results of QC analysis** |  |  |  |  |  |  |
| Number of samples | 0 | 686 | 0 | 194 | 0 | 880 |
| * Samples with a number of CNV calls above the 95th percentile in any of the three algorithms (72, 45 and 53 CNVs for PennCNV, Birdsuite and iPattern respectively), were removed | | | | | | |

**Supplementary Table 2**: Quality control (QC) analysis steps for CNVs for the stringent dataset

| **QC Evaluation (CNVs filters)** | **Autosomal CNVs** | | **Chr. X CNVs** | | **Total CNVs** | |
| --- | --- | --- | --- | --- | --- | --- |
|  | **# CNVs removed** | **# CNVs after QC** | **# CNVs removed** | **# CNVs after QC** | **# CNVs removed** | **# CNVs after QC** |
| Number of CNVs | 0 | 46,908 | 0 | 978 | 0 | 47,886 |
| **QC CNVs selection** |  |  |  |  |  |  |
| CNVs detected by one algorithm | 29,313 | 17,595 | 846 | 132 | 30,159 | 17,727 |
| CNVs located at pseudoautosomal region | 0 | 17,595 | 11 | 121 | 11 | 17,716 |
| CNVs located at immunoglobulin region | 203 | 17,392 | 0 | 121 | 203 | 17,513 |
| CNVs located at chromosome's pq arms | 3 | 17,389 | 0 | 121 | 3 | 17,510 |
| CNVs with gap overlapping >50% | 0 | 17,389 | 0 | 121 | 0 | 17,510 |
| Gain/loss CNVs | 81 | 17,308 | 0 | 121 | 81 | 17,429 |
| CNVs with length < 100kb | 13,360 | 3,948 | 46 | 75 | 13,406 | 4,023 |
| **Rare CNVs selection** |  |  |  |  |  |  |
| Common CNVs | 3,246 | 702 | 9 | 66 | 3,255 | 768 |
| CNVs with segmental duplications overlapping >50% | 110 | 592 | 1 | 65 | 111 | 657 |
| **Final results of CNVs QC evaluation** |  |  |  |  |  |  |
| Number of CNVs | 0 | 592 | 0 | 65 | 0 | 657 |

**Supplementary Table 3**: List of known T2D genes.

| **Gene symbol or Locus** | **Type** | **Cytoband** | **Associated condition** | **CNV type** | **Clinical association** | **Population** | **Reference** |
| --- | --- | --- | --- | --- | --- | --- | --- |
| ***ABCC8*** | Gene | 11p15.1 | Familial study | NA | PNDM | European | Babenko et.al 2006 |
| ***ADAMTS9*** | Gene | 3p14.1 | GWAS finding | NA | T2D | European | Zeggini et al., 2008 |
| ***ADCY5*** | Gene | 3q21.1 | GWAS finding | NA | T2D | European | Dupuis et al., 2010 |
| ***ADRB3*** | Gene | 8p12 | GWAS finding | NA | T2D | Mexican | Cruz et al. 2010 |
| ***AGPAT2*** | Gene | 9q34.3 | GWAS finding | NA | BSS | European | Agarwal et.al 2002 |
| ***AKNAD1*** | CNV associated | 1p13.3 | Case-control study | Deletion | T2D | Jordan | Dajani et.al 2015 |
| ***AKT2*** | Gene | 19q13.2 | GWAS finding | NA | T2D | European | George et.al 2004 |
| ***ALMS1*** | Gene | 2p13.2 | Familial study | NA | AS | European | Minton et.al 2006 |
| ***AMY2A*** | CNV associated | 1p21.1 | Case-control study | Duplication | T2D end stage renal disease | African Americans | Bailey et.al 2013 |
| ***AMY2B*** | CNV associated | 1p21.1 | Case-control study | Duplication | T2D end stage renal disease | African Americans | Bailey et.al 2013 |
| ***ANK1*** | Gene | 8p11.1 | GWAS finding | NA | T2D | European, East Asian | Harder et al., 2013; Imamura et al., 2012 |
| ***ANKRD55*** | Gene | 5q11.2 | GWAS finding | NA | T2D | European | Harder et al., 2013 |
| ***AP3S2*** | Gene | 15q26.1 | GWAS finding | NA | T2D | East Asian | Kooner et al., 2011 |
| ***ARAP1*** | Gene | 11q13.4 | GWAS finding | NA | T2D | European | Voigh et al., 2010 |
| ***ARL15*** | Gene | 5p15.2 | GWAS finding | NA | T2D adiponectin levels | European | Richards et al., 2009 |
| ***BCAR1*** | Gene | 16q23.1 | GWAS finding | NA | T2D | European | Harder et al., 2013 |
| ***BCL11A*** | Gene | 2p16.1 | GWAS finding | NA | T2D | European | Voigh et al., 2010 |
| ***BSCL2*** | Gene | 11q12.3 | Familial study | NA | T1D | East Asian | Jin et al., 2007 |
| ***C2CD4A*** | Gene | 15q22.2 | GWAS finding | NA | T2D | European | Strawbridge et al., 2011 |
| ***CAPN10*** | Gene | 2q37.3 | Case-control study | NA | T2D | Mexican | del Bosque et al., 2004 |
| ***CAV1*** | Gene | 7q31.2 | Familial study | NA | BSS | Hispanic-Latino | Kim et al., 2008 |
| ***CCND2*** | Gene | 12p13 | GWAS finding | NA | T2D | European | Perry et al., 2009 |
| ***CDC123*** | Gene | 10p13 | GWAS finding | NA | T2D | European | Zeggini et al., 2008 |
| ***CDKAL1*** | Gene | 6p22.3 | GWAS finding | NA | T2D | European | Diabetes Genetics Initiative et al,. 2007; Scott el al., 2007; Zeggini et al., 2007; Steinhorsdottir et al., 2007 |
| ***CDKN2A*** | Gene | 9p21.3 | GWAS finding | NA | T2D | European | Diabetes Genetics Initiative et al,. 2007;Scott el al., 2007; Zeggini et al., 2007 |
| ***CEL*** | Gene | 9q34.2 | Familial study | NA | MODY | European | Torsvik et al., 2010 |
| ***CILP2*** | Gene | 19p13.11 | GWAS finding | NA | T2D | European | Harder et al., 2013 |
| ***CISD2*** | Gene | 4q24 | GWAS finding | NA | WS | European | Chen et al., 2010 |
| ***DGKB*** | Gene | 7p21.2 | GWAS finding | NA | T2D | European | Dupuis et al., 2010 |
| ***DUSP8*** | Gene | 11p15.5 | GWAS finding | NA | T2D | European | Kong et al. 2009 |
| ***DUSP9*** | Gene | Xq28 | GWAS finding | NA | T2D | European | Voigh et al., 2010 |
| ***EIF2AK3*** | Gene | 2p11.2 | Familial study | NA | WRS | European | Brickwood et al., 2003 |
| ***ENPP1*** | Gene | 6q22-q23 | Cohort study | NA | T2D | European | Kubaszek et al., 2004 |
| ***FAF1*** | Gene | 1p33 | GWAS finding | NA | T2D | European | DIAbetes Genetics Replication et al., 2014 |
| ***FTO*** | Gene | 16q12.2 | GWAS finding | NA | T2D | European | Frayling et al., 2007 |
| ***FXN*** | Gene | 9q21.11 | Familial study | NA | FA | European | Campuzano et al., 1996 |
| ***GCC1*** | Gene | 7q32.1 | GWAS finding | NA | T2D | East Asian | Cho et al., 2011 |
| ***GCGR*** | Gene | 17q25 | Familial study | NA | T2D | European | Hager et al., 1995 |
| ***GCK*** | Gene | 7p13 | GWAS finding | NA | T2D | European | Dupuis et al., 2010 |
| ***GCKR*** | Gene | 2p23.3 | GWAS finding | NA | T2D | European | Dupuis et al., 2010 |
| ***GIPR*** | Gene | 19q13.3 | GWAS finding | NA | T2D glucose metabolism | East Asian | Hu et al., 2010 |
| ***GLIS3*** | Gene | 9p24.2 | GWAS finding | NA | T2D | East Asian | Cho et al., 2011 |
| ***GPD2*** | Gene | 2q24.1 | Single case | NA | T2D | European | Novials et al., 1997 |
| ***GRB14*** | Gene | 2q22-q24 | GWAS finding | NA | T2D | East Asian | Kooner et al., 2011 |
| ***GRK5*** | Gene | 10q26.11 | GWAS finding | NA | T2D | East Asian | Li et al., 2013 |
| ***HFE*** | Gene | 6p22.1 | Case-control study | NA | T2D | European | Qi et al., 2005 |
| ***HHEX*** | Gene | 10q23.33 | GWAS finding | NA | T2D | European | Sladker et al., 2007 |
| ***HMG20A*** | Gene | 15q24 | GWAS finding | NA | T2D | European, East Asian | Harder et al., 2013; Kooner et al., 2011 |
| ***HMGA1*** | Gene | 6p21 | Case-control study | NA | T2D | European | Chiefari et al., 2011 |
| ***HMGA2*** | Gene | 12q14.3 | GWAS finding | NA | T2D | European | Voigh et al., 2010 |
| ***HNF1A*** | Gene | 12q24.31 | GWAS finding | NA | T2D | European | Voigh et al., 2010; Sandhu et al., 2007; Winckler et al., 2007; Gudmundsson et al., 2007; Franks et al., 2008 |
| ***HNF1B*** | Gene | 17q12 | GWAS finding | NA | T2D | European | Sandhu et al., 2007; Winckler et al., 2007; Gudmundsson et al., 2007; Franks et al., 2008 |
| ***HNF4A*** | Gene | 20q13.12 | Familial study | NA | MODY | European | Ellard et al., 2006 |
| ***IGF2BP2*** | Gene | 3q27.2 | GWAS finding | NA | T2D | European | Diabetes Genetics Initiative et al,. 2007;Scott el al., 2007; Zeggini et al., 2007 |
| ***INPP5B*** | Gene | 1p34.3 | CNV tagging SNPs | Intronic rs16824514 | T2D | European | Chen et al.,2010 |
| ***INS*** | Gene | 11p15.5 | Familial study | NA | PNDM, MODY | European | Edghill et al., 2008; Molven et al.,2008 |
| ***INSR*** | Gene | 19p13.2 | GWAS finding | NA | T2D | European | Barroso et al., 2003 |
| ***IRS1*** | Gene | 2q36.3 | GWAS finding | NA | T2D | European | Rung et al., 2009. |
| ***IRS2*** | Gene | 13q34 | Case-control study | NA | T2D | European, East Asian | Mammarella et al., 2000|Wang et al., 2001 |
| ***JAZF1*** | Gene | 7p15.1 | GWAS finding | NA | T2D | European | Zeggini et al., 2008 |
| ***KCNIP1*** | CNV associated | 5q35.1 | Case-control study | Deletion | T2D | East Asian | Lee et.al 2014 |
| ***KCNJ11*** | Gene | 11p15.1 | GWAS finding | NA | T2D | European | Gloyn et al., 2000 |
| ***KCNK16*** | Gene | 6p21.2-p21.1 | GWAS finding | NA | T2D | East Asian | Cho et al., 2011 |
| ***KCNQ1*** | Gene | 11p15.4 | GWAS finding | NA | T2D | East Asian | Yasuda et al., 2008; Unoki et al., 2088 |
| ***KLF14*** | Gene | 7q32.2 | GWAS finding | NA | T2D | European | Voigh et al., 2010 |
| ***KLHDC5*** | Gene | 12p11.22 | GWAS finding | NA | T2D | European | Harder et al., 2013 |
| ***LEPR*** | Gene | 1p31 | Case-control study | Low CN | T2D | East Asian | Jeon et al., 2010 |
| ***LIPC*** | Gene | 15q21-q23 | Case-control study | NA | T2D | European | Todorova et al., 2004 | Garup et al., 2008 |
| ***LMNA*** | Gene | 1q22 | GWAS finding | NA | T2D | European | Wegner et al., 2007 |
| ***LMNB2*** | Gene | 19p13.3 | Case-control study | NA | BRSS | European | Hegele et al., 2006 |
| ***LPP*** | Gene | 3q28 | GWAS finding | NA | T2D | European | DIAbetes Genetics Replication et al., 2014 |
| ***MAEA*** | Gene | 4p16.3 | GWAS finding | NA | T2D | East Asian | Cho et al., 2011 |
| ***MAPK8IP1*** | Gene | 11p11.2 | Familial study | NA | T2D | European | Waeber et al., 2000 |
| ***MC4R*** | Gene | 18q22 | GWAS finding | NA | T2D | European | Xi et al., 2012 |
| ***MGEA5*** | Gene | 10q24.1-q24.3 | Case-control study | NA | T2D | Mexican | Cameron et al., 2007 |
| ***MOSC2*** | Gene | 1q41 | CNV tagging SNPs | intronic rs337147 | T2D | European | Chen et al.,2010 |
| ***MPHOSPH9*** | Gene | 12q24.31 | GWAS finding | NA | T2D | European | DIAbetes Genetics Replication et al., 2014 |
| ***MTNR1B*** | Gene | 11q21 | GWAS finding | NA | T2D | European | Bouatia-Naji et al., 2009; Lyssenko et al., 2009; Prokopenko et al., 2009 |
| ***NEUROD1*** | Gene | 2q31.3 | Familial study | NA | MODY | European | Kristinsson et al., 2001 |
| ***NOTCH2*** | Gene | 1p12 | GWAS finding | NA | T2D | European | Zeggini et al., 2008 |
| ***PAX4*** | Gene | 7q32 | Case-control study | NA | T2D, MODY type 9 | East Asian | Shimajiri et al., 2001|Plengvidhya, et al., 2007 |
| ***PDX1*** | Gene | 13q12.2 | Familial study | NA | PNDM | European | Nicolino et al., 2010 |
| ***PEPD*** | Gene | 19q13.11 | GWAS finding | NA | T2D | East Asian | Cho et al., 2011 |
| ***PLAGL1*** | Gene | 6q24.2 | Familial study | NA | TNDM | East Asian | Kamiya et al., 2000 |
| ***POU5F1/TCF19*** | Gene | 6p21.3 | GWAS finding | NA | T1D | European | Cheung et al., 2011 |
| ***PPARG*** | Gene | 3p25.1 | GWAS finding | NA | T2D | European | Altshuler et al., 2000 |
| ***PPP1R3A*** | Gene | 7q31.1 | Case-control study | NA | Insulin resistance | European | Hansen et.al., 1995 |
| ***PRC1*** | Gene | 15q26.1 | GWAS finding | NA | T2D | European | Voigh et al., 2010 |
| ***PROX1*** | Gene | 1q41 | GWAS finding | NA | T2D | European | Dupuis et al., 2010 |
| ***PSMD6*** | Gene | 3p14.1 | GWAS finding | NA | T2D | East Asian | Cho et al., 2011 |
| ***PTPN1*** | Gene | 20q13.1-q13.2 | Case-control study | NA | T2D | European | Mok et al., 2002 |
| ***PTPRD*** | Gene | 9p23 | GWAS finding | NA | T2D | East Asian | Tsai et al., 2010 |
| ***RASGRP1*** | Gene | 15q14 | GWAS finding | NA | T2D | East Asian | Li et al., 2013 |
| ***RBMS1*** | Gene | 2q24.2 | GWAS finding | NA | T2D | European | Qi et al., 2010 |
| ***RETN*** | Gene | 19p13.2 | Case-control study | NA | T2D | East Asian | Tan et al., 2003 |
| ***RFX6*** | Gene | 6q22.2 | Familial study | NA | MRS | European | Pearl et al., 2011 |
| ***RND3*** | Gene | 2q23.3 | GWAS finding | NA | T2D | African Americans | Palmer et al., 2012 |
| ***SIRT1*** | Gene | 10q21.3 | GWAS finding | NA | T2D | Mexican | Cruz et al. 2010 |
| ***SLC2A2*** | Gene | 3q26.2 | Single case | NA | T2D | European | Mueckler et.al., 1994 |
| ***SLC2A4*** | Gene | 17p13 | Case-control study | NA | T2D | European | Kusari et al., 1991 |
| ***SLC30A8*** | Gene | 8q24.11 | GWAS finding | NA | T2D | European | Sladker et al., 2007 |
| ***SPRY2*** | Gene | 13q31.1 | Case-control study | NA | T2D | East Asian | Imamura et al., 2011 |
| ***SRR*** | Gene | 17p13.3 | GWAS finding | NA | T2D | East Asian | Tsai et al., 2010 |
| ***SSR1/RREB1*** | Gene | 6p24.3 | GWAS finding | NA | T2D | Mexican | Below et al., 2011 |
| ***ST6GAL1*** | Gene | 3q27-q28 | GWAS finding | NA | T2D | East Asian | Kooner et al., 2011 |
| ***TCF7L2*** | Gene | 10q25.2 | GWAS finding | NA | T2D | European, Mexican | Grant el al., 2007; Cruz et al. 2010 |
| ***THADA*** | Gene | 2p21 | GWAS finding | NA | T2D | European | Zeggini et al., 2008 |
| ***TLE1*** | Gene | 9q21.32 | GWAS finding | NA | T2D | European | Morris et al., 2012 |
| ***TLE4*** | Gene | 9q21.31 | GWAS finding | NA | T2D | European | de Miguel-Yanes et al., 2012 |
| ***TMEM154*** | Gene | 4q31.3 | GWAS finding | NA | T2D | European | DIAbetes Genetics Replication et al., 2014 |
| ***TMEM163*** | Gene | 2q21.3 | GWAS finding | NA | T2D | Indian | Tabassum et al., 2013 |
| ***TNF*** | Gene | 6p21.3 | Case-control study | NA | T2D | Mexican | Perez-Luque et al., 2011; Guzmán-Flores et al., 2011 |
| ***TP53INP1*** | Gene | 8q22.1 | GWAS finding | NA | T2D | European | DIAbetes Genetics Replication et al., 2014 |
| ***TSPAN8*** | Gene | 12q21.1 | CNV tagging SNPs | exonic rs1798090, CNVR5583.1 | T2D | European | Wellcome Trust Case Control Consortium, et al. 2010 |
| ***UBE2E2*** | Gene | 3p24.2 | GWAS finding | NA | T2D | East Asian | Yamauchi et al., 2010 |
| ***VPS26A*** | Gene | 10q21.1 | GWAS finding | NA | T2D | East Asian | Kooner et al., 2011 |
| ***WFS1*** | Gene | 4p16.1 | GWAS finding | NA | T2D | European | Sandhu et al., 2007; Winckler et al., 2007; Gudmundsson et al., 2007; Franks et al., 2008 |
| ***ZBED3*** | Gene | 5q14.1 | GWAS finding | NA | T2D | European | Voigh et al., 2010 |
| ***ZFAND3*** | Gene | 6p21.2 | GWAS finding | NA | T2D | East Asian | Cho et al., 2011 |
| ***ZFAND6*** | Gene | 15q25.1 | GWAS finding | NA | T2D | European | Voigh et al., 2010 |
| ***ZMIZ1*** | Gene | 10q22.3 | GWAS finding | NA | T2D | European | Harder et al., 2013 |
| **chr4:550000-1850000** | Locus | 4p16.3 | Case-control study | Deletion in 4p16.3 region | T2D | East Asian | Kudo et al., 2011 |
| **chr9:81041948-81241948** | Locus | 9q21.31 | GWAS finding | NA | T2D | European | Voigh et al., 2010 |
| **chr15:45994758-45999227** | Locus | 15q21.1 | Case-control study | Deletion | T2D | East Asian | Bae et al., 2011 |
| **chr16:28472750-28510612** | Gene | 16p11.2 | Case-control study | CNVR6685.1 | T2D | European | Grassi et al., 2011 |
| **chr16:86950000-88700000** | CNV associated | 16q24.2-3 | Case-control study | Deletion | T2D | East Asian | Kodama et.al 2014 |
| **chr19:46794847-46894847** | Locus | 19q13.2 | CNV associated | intergenic rs2016070 | T2D | European | Chen et al.,2010 |
| **chr22:20722473-21702142** | Locus | 22q11.22 | Case-control study | Deletion | T2D | East Asian | Bae et al., 2011 |
| **chr22:3559620-3561217** | Locus | 22q11.22 | Case-control study | Deletion | T2D | East Asian | Bae et al., 2011 |

AS: Alström syndrome; BSS: Berardinelli-Seip syndrome; BRSS: Barraquer–Simons syndrome; FA: Friedreich's ataxia; GWAS: genome-wide association study; MODY: maturity-onset diabetes of the young; MRS: Mitchell–Riley syndrome; PNDM: permanent neonatal diabetes mellitus; T1D: type 1 diabetes; T2D: type 2 diabetes; WRS: Wolcott-Rallison syndrome; WS: Wolfram syndrome; NA: not available

**Supplementary Table 4**: Rare genic CNVs seen in T2D group but not in control group

| **CNV type** | **Location** | **Coordinates^1^** | **Size (Mb)** | **Number of genes** | **Genes** | **Genes with OMIM IDs** | **DGV frequency (%)^2^** |
| --- | --- | --- | --- | --- | --- | --- | --- |
| Duplication | 1p36.32 | chr1:2,319,424-2,492,802 | 173.4 | 7 | *PANK4, PLCH2, TNFRSF14, LOC115110, PEX10, RER1, HES5* | *PANK4 (#606162), PLCH2 (#612836), TNFRSF14 (#602746), PEX10 (#602859), HES5 (#607348)* | 0.0081 |
| Duplication | 1q44 | chr1:246,051,092-246,521,546 | 470.5 | 16 | *OR2L1P, OR2L13, OR2M1P, OR2M3, OR11L1, OR2M5, OR2M4, OR2L2, TRIM58, OR2T8, OR2M2, OR2W3, OR2AK2, OR2T33, OR2L8, OR2L3* | *OR2W3 (#616729)* | 0.0055 |
| Duplication | 1q44 | chr1:246,253,723-246,853,119 | 599.4 | 22 | *OR2L13, OR2L2, OR2L3, OR2T3, OR2T10, OR2T34, OR2T33, OR2G6, OR14C36, OR2M5, OR2M4, OR2M7, OR2M3, OR2M2, OR2T6, OR2T4, OR2T5, OR2T2, OR2T12, OR2T1, OR2M1P, OR2T29* | *-* | 0.0130 |
| Duplication | 1q44 | chr1:246,488,966-246,750,023 | 261.1 | 10 | *OR2T5, OR2T33, OR2M7, OR2T6, OR2T12, OR14C36, OR2T2, OR2T4, OR2T3, OR2T1* | *-* | 0.0172 |
| Duplication | 1q44 | chr1:246,488,966-246,669,603 | 180.6 | 7 | *OR2T33, OR2M7, OR2T6, OR2T12, OR14C36, OR2T4, OR2T1* | *-* | 0.0183 |
| Deletion | 2p16.3 | chr2:50,756,435-50,916,417 | 160 | 1 | *NRXN1* | *NRXN1 (#600565)* | 0.0050 |
| Duplication | 2q13 | chr2:110,173,879-110,519,409 | 345.5 | 4 | *NCRNA00116, MALL, MIR4267, NPHP1* | *MALL (#602022), NPHP1 (#607100)* | 0.0096 |
| Duplication | 2q13 | chr2:110,173,879-110,519,409 | 345.5 | 4 | *NCRNA00116, MALL, MIR4267, NPHP1* | *MALL (#602022), NPHP1 (#607100)* | 0.0096 |
| Duplication | 2q13 | chr2:110,173,879-110,519,409 | 345.5 | 4 | *NCRNA00116, MALL, MIR4267, NPHP1* | *MALL (#602022), NPHP1 (#607100)* | 0.0096 |
| Duplication | 2q13 | chr2:110,190,851-110,337,635 | 146.8 | 3 | *MALL, NCRNA00116, NPHP1* | *MALL (#602022), NPHP1 (#607100)* | 0.0177 |
| Duplication | 2q13 | chr2:110,206,673-110,340,647 | 134 | 3 | *MALL, NCRNA00116, NPHP1* | *MALL (#602022), NPHP1 (#607100)* | 0.0194 |
| Duplication | 3p26.3 | chr3:839,367-1,403,263 | 563.9 | 1 | *CNTN6* | *CNTN6 (#607220)* | 0.0055 |
| Deletion | 3p26.3 | chr3:2,333,948-2,458,305 | 124.4 | 1 | *CNTN4* | *CNTN4 (#607280)* | 0.0088 |
| Duplication | 3p25.1 | chr3:12,611,255-12,781,080 | 169.8 | 2 | *RAF1, TMEM40* | *RAF1 (#164760)* | 0.0041 |
| Duplication | 3p25.1 | chr3:12,611,255-12,781,080 | 169.8 | 2 | *RAF1, TMEM40* | *RAF1 (#164760)* | 0.0041 |
| Duplication | 3p25.1 | chr3:12,611,255-12,781,080 | 169.8 | 2 | *RAF1, TMEM40* | *RAF1 (#164760)* | 0.0041 |
| Duplication | 3p25.1 | chr3:12,611,255-12,746,049 | 134.8 | 1 | *RAF1* | *RAF1 (#164760)* | 0.0030 |
| Duplication | 3p25.1 | chr3:12,611,255-12,781,080 | 169.8 | 2 | *RAF1, TMEM40* | *RAF1 (#164760)* | 0.0041 |
| Duplication | 3p25.1 | chr3:12,611,255-12,789,263 | 178 | 2 | *RAF1, TMEM40* | *RAF1 (#164760)* | 0.0039 |
| Duplication | 3p25.1 | chr3:12,622,076-12,781,080 | 159 | 2 | *RAF1, TMEM40* | *RAF1 (#164760)* | 0.0044 |
| Deletion | 3p14.2 | chr3:60,048,193-60,165,656 | 117.5 | 1 | *FHIT* | *FHIT (#601153)* | 0.0017 |
| Duplication | 3p14.1 | chr3:67,887,469-68,390,184 | 502.7 | 1 | *FAM19A1* | *-* | 0.0029 |
| Duplication | 3p14.1 | chr3:67,945,122-68,495,977 | 550.9 | 1 | *FAM19A1* | *-* | 0.0017 |
| Duplication | 3p14.1 | chr3:67,945,122-69,066,376 | 1121.3 | 2 | *FAM19A4, FAM19A1* | *-* | 0.0114 |
| Duplication | 3p14.1 | chr3:68,517,293-69,076,570 | 559.3 | 2 | *FAM19A4, FAM19A1* | *-* | 0.0440 |
| Duplication | 3q13.2 | chr3:113,575,498-113,708,624 | 133.1 | 1 | *BTLA* | *BTLA (#607925)* | 0.0294 |
| Duplication | 3q13.2 | chr3:113,585,255-113,708,624 | 123.4 | 1 | *BTLA* | *BTLA (#607925)* | 0.0294 |
| Duplication | 3q13.2 | chr3:113,585,255-113,708,624 | 123.4 | 1 | *BTLA* | *BTLA (#607925)* | 0.0294 |
| Duplication | 3q13.2 | chr3:113,585,255-113,708,624 | 123.4 | 1 | *BTLA* | *BTLA (#607925)* | 0.0294 |
| Duplication | 3q13.2 | chr3:113,585,255-113,708,624 | 123.4 | 1 | *BTLA* | *BTLA (#607925)* | 0.0294 |
| Duplication | 3q13.2 | chr3:113,585,255-113,708,624 | 123.4 | 1 | *BTLA* | *BTLA (#607925)* | 0.0294 |
| Duplication | 3q13.2 | chr3:113,594,489-113,708,624 | 114.1 | 1 | *BTLA* | *BTLA (#607925)* | 0.1953 |
| Duplication | 5p13.2 | chr5:37,193,280-37,628,809 | 435.5 | 3 | *WDR70, NUP155, C5orf42* | *WDR70 (#617233), NUP155 (#606694), C5orf42 (#614571)* | 0.0298 |
| Duplication | 5q15 | chr5:96,167,578-96,491,379 | 323.8 | 4 | *LNPEP, ERAP2, ERAP1, LIX1* | *LNPEP (#151300), ERAP2 (#609497), ERAP1 (#606832), LIX1 (#610466)* | 0.0179 |
| Duplication | 6q11.1 | chr6:61,943,890-62,511,387 | 567.5 | 2 | *MTRNR2L9, KHDRBS2* | *KHDRBS2 (#610487)* | 0.0032 |
| Duplication | 6q26 | chr6:162,665,597-162,990,011 | 324.4 | 1 | *PARK2* | *-* | 0.0074 |
| Duplication | 6q26 | chr6:162,685,017-162,891,538 | 206.5 | 1 | *PARK2* | *-* | 0.0082 |
| Duplication | 7q11.21 | chr7:64,289,075-64,725,571 | 436.5 | 2 | *INTS4L1, ZNF92* | *ZNF92 (#603974)* | 0.0115 |
| Deletion | 7q11.21 | chr7:64,289,075-64,725,571 | 436.5 | 2 | *INTS4L1, ZNF92* | *ZNF92 (#603974)* | 0.0115 |
| Deletion | 7q31.1 | chr7:110,651,112-110,977,623 | 326.5 | 1 | *IMMP2L* | *IMMP2L (#605977)* | 0.0095 |
| Deletion | 7q31.1 | chr7:110,727,055-111,109,991 | 382.9 | 1 | *IMMP2L* | *IMMP2L (#605977)* | 0.0089 |
| Duplication | 10q26.3 | chr10:135,029,305-135,356,694 | 327.4 | 15 | *DUX4L7, SPRN, PAOX, DUX4, DUX4L6, FRG2B, CYP2E1, MTG1, SYCE1, ECHS1, LOC619207, DUX4L3, DUX4L2, SPRNP1, DUX4L5* | *SPRN (#610447), PAOX (#615853), DUX4 (#606009), CYP2E1 (#124040), SYCE1 (#611486), ECHS1 (#602292)* | 0.0312 |
| Duplication | 11p15.5 | chr11:1,053,767-1,258,853 | 205.1 | 3 | *MUC2, TOLLIP, MUC5B* | *MUC2 (#158370), TOLLIP (#606277), MUC5B (#600770)* | 0.0068 |
| Duplication | 11p14.3 | chr11:24,734,687-24,864,737 | 130.1 | 1 | *LUZP2* | *LUZP2 (#608178)* | 0.0053 |
| Duplication | 11p14.3 | chr11:24,750,900-24,866,240 | 115.3 | 1 | *LUZP2* | *LUZP2 (#608178)* | 0.0028 |
| Duplication | 11p14.3 | chr11:24,750,900-24,866,240 | 115.3 | 1 | *LUZP2* | *LUZP2 (#608178)* | 0.0028 |
| Duplication | 11p11.12 | chr11:50,052,671-50,626,381 | 573.7 | 2 | *LOC441601, LOC646813* | *-* | 0.0058 |
| Duplication | 11p11.12 | chr11:50,097,032-50,567,700 | 470.7 | 2 | *LOC441601, LOC646813* | *-* | 0.0049 |
| Duplication | 11p11.12 | chr11:50,105,549-50,703,817 | 598.3 | 2 | *LOC441601, LOC646813* | *-* | 0.0048 |
| Duplication | 11p11.12 | chr11:50,227,128-50,626,381 | 399.3 | 1 | *LOC646813* | *-* | 0.0068 |
| Duplication | 13q14.3 | chr13:50,927,592-51,386,544 | 459 | 4 | *NCRNA00282, DHRS12, WDFY2, CCDC70* | *DHRS12 (#616163), WDFY2 (#610418)* | 0.0176 |
| Duplication | 13q33.3 | chr13:106,571,549-106,993,097 | 421.5 | 1 | *FAM155A* | *-* | 0.0037 |
| Duplication | 15q13.3 | chr15:29,754,102-30,298,896 | 544.8 | 1 | *CHRNA7* | *CHRNA7 (#118511)* | 0.0097 |
| Duplication | 15q13.3 | chr15:29,796,277-30,337,472 | 541.2 | 1 | *CHRNA7* | *CHRNA7 (#118511)* | 0.0103 |
| Duplication | 15q13.3 | chr15:29,798,781-30,298,896 | 500.1 | 1 | *CHRNA7* | *CHRNA7 (#118511)* | 0.0106 |
| Duplication | 15q13.3 | chr15:29,798,781-30,207,546 | 408.8 | 1 | *CHRNA7* | *CHRNA7 (#118511)* | 0.0056 |
| Duplication | 15q13.3 | chr15:29,806,023-30,337,472 | 531.5 | 1 | *CHRNA7* | *CHRNA7 (#118511)* | 0.0105 |
| Duplication | 15q13.3 | chr15:29,806,023-30,298,896 | 492.9 | 1 | *CHRNA7* | *CHRNA7 (#118511)* | 0.0108 |
| Duplication | 16p13.2 | chr16:6,691,895-7,055,720 | 363.8 | 1 | *RBFOX1* | *RBFOX1 (#605104)* | 0.0078 |
| Duplication | 16p13.2 | chr16:6,691,895-7,063,647 | 371.8 | 1 | *RBFOX1* | *RBFOX1 (#605104)* | 0.0078 |
| Duplication | 16p13.2 | chr16:6,691,895-7,055,720 | 363.8 | 1 | *RBFOX1* | *RBFOX1 (#605104)* | 0.0078 |
| Duplication | 16p13.2 | chr16:6,691,895-7,055,720 | 363.8 | 1 | *RBFOX1* | *RBFOX1 (#605104)* | 0.0078 |
| Duplication | 16p13.2 | chr16:6,691,895-7,063,647 | 371.8 | 1 | *RBFOX1* | *RBFOX1 (#605104)* | 0.0078 |
| Duplication | 16p13.2 | chr16:6,698,381-7,055,720 | 357.3 | 1 | *RBFOX1* | *RBFOX1 (#605104)* | 0.0080 |
| Duplication | 17q25.3 | chr17:76,914,079-77,124,208 | 210.1 | 6 | *C17orf70, ACTG1, BAHCC1, MIR3186, FSCN2, TMEM105* | *ACTG1 (#102560), FSCN2 (#607643)* | 0.0138 |
| Duplication | 18q23 | chr18:75,684,875-75,904,807 | 219.9 | 5 | *HSBP1L1, TXNL4A, KCNG2, C18orf22, PQLC1* | *TXNL4A (#611595), KCNG2 (#605696)* | 0.0524 |
| Duplication | 18q23 | chr18:75,694,643-75,904,807 | 210.2 | 5 | *HSBP1L1, TXNL4A, KCNG2, C18orf22, PQLC1* | *TXNL4A (#611595), KCNG2 (#605696)* | 0.0524 |
| Duplication | 18q23 | chr18:75,694,643-75,904,807 | 210.2 | 5 | *HSBP1L1, TXNL4A, KCNG2, C18orf22, PQLC1* | *TXNL4A (#611595), KCNG2 (#605696)* | 0.0524 |
| Duplication | 18q23 | chr18:75,694,643-75,904,807 | 210.2 | 5 | *HSBP1L1, TXNL4A, KCNG2, C18orf22, PQLC1* | *TXNL4A (#611595), KCNG2 (#605696)* | 0.0524 |
| Deletion | 20p12.1 | chr20:14,587,135-15,019,192 | 432.1 | 1 | *MACROD2* | *MACROD2 (#611567)* | 0.0092 |
| Duplication | 22q11.23 | chr22:22,463,570-22,670,785 | 207.2 | 9 | *GSTT2B, GSTT2, DDTL, SLC2A11, MIF, GSTTP1, DERL3, SMARCB1, DDT* | *GSTT2 (#600437), SLC2A11 (#610367), MIF (#153620), DERL3 (#610305), SMARCB1 (#601607), DDT (#602750)* | 0.0631 |
| Deletion | Xq21.31 | chrX:87,724,805-88,045,123 | 320.3 | 1 | *CPXCR1* | *-* | 0.0000 |
| Deletion | Xq21.31 | chrX:87,724,805-88,045,123 | 320.3 | 1 | *CPXCR1* | *-* | 0.0000 |
| Deletion | Xq21.31 | chrX:87,724,805-88,045,123 | 320.3 | 1 | *CPXCR1* | *-* | 0.0000 |
| Deletion | Xq21.31 | chrX:87,724,805-88,045,123 | 320.3 | 1 | *CPXCR1* | *-* | 0.0000 |
| Duplication | Xq28 | chrX:153,208,580-153,357,637 | 149.1 | 11 | *EMD, FAM50A, PLXNA3, TKTL1, ATP6AP1, TAZ, RPL10, DNASE1L1, FLNA, GDI1, SNORA70* | *EMD (#300384), FAM50A (#300453), PLXNA3 (#300022), TKTL1 (#300044), ATP6AP1 (#300197), TAZ (#300394), RPL10 (#312173), DNASE1L1 (#300081), FLNA (#300017), GDI1 (#300104)* | 0.0038 |
| ^1^ Coordinates according NCBI v36, hg18 | | | | | | | |
| ^2^ Frequency according to the Database of Genomic Variants (DGV) | | | | | | | |

**Supplementary Table 5**: Results of the Gene-set analysis.

| **Gene-set name** | **Gene-set ID** | **Number of genes** | **Genes ^1^** | **CNV type** | **Cluster name** |
| --- | --- | --- | --- | --- | --- |
| Signal Transduction | 485565 | 28 | *OR14C36, OR2AK2, OR2G6, OR2L2, OR2L3, OR2L8, OR2M2, OR2M3, OR2M4, OR2M5, OR2M7, OR2T1, OR2T10, OR2T12, OR2T2, OR2T29, OR2T3, OR2T33, OR2T34, OR2T4, OR2T5, OR2T6, OR2T8, OR2W3, OR4A5, OR4C12, RAF1, GDI1* | Duplications | Signal transducer activity transmembrane |
| Signaling by GPCR | 485692 | 26 | *OR14C36, OR2AK2, OR2G6, OR2L2, OR2L3, OR2L8, OR2M2, OR2M3, OR2M4, OR2M5, OR2M7, OR2T1, OR2T10, OR2T12, OR2T2, OR2T29, OR2T3, OR2T33, OR2T34, OR2T4, OR2T5, OR2T6, OR2T8, OR2W3, OR4A5, OR4C12* | Duplications | Signaling G-Protein Coupled GPCR |
| GPCR downstream signaling | 485711 | 26 | *OR14C36, OR2AK2, OR2G6, OR2L2, OR2L3, OR2L8, OR2M2, OR2M3, OR2M4, OR2M5, OR2M7, OR2T1, OR2T10, OR2T12, OR2T2, OR2T29, OR2T3, OR2T33, OR2T34, OR2T4, OR2T5, OR2T6, OR2T8, OR2W3, OR4A5, OR4C12* | Duplications | Signaling G-Protein Coupled GPCR |
| Olfactory Signaling Pathway | 485724 | 26 | *OR14C36, OR2AK2, OR2G6, OR2L2, OR2L3, OR2L8, OR2M2, OR2M3, OR2M4, OR2M5, OR2M7, OR2T1, OR2T10, OR2T12, OR2T2, OR2T29, OR2T3, OR2T33, OR2T34, OR2T4, OR2T5, OR2T6, OR2T8, OR2W3, OR4A5, OR4C12* | Duplications | Genes involved olfactory pathway |
| System process | GO:0003008 | 40 | *OR11L1, NPHP1, OR14C36, OR2AK2, OR2G6, OR2L2, KCNG2, OR2L3, OR2L8, OR2M2, OR2M3, OR2M4, OR2M5, OR2M7, HES5, OR2T1, OR2T10, EMD, OR2T12, OR2T2, OR2T29, OR2T3, OR2T33, OR2T34, OR2T4, OR2T5, OR2T6, OR2T8, OR2W3, OR4A5, OR4C12, CHRNA7, GDNF, RAF1, TAZ, ERAP1, NRXN1, ERAP2, NIPBL, FSCN2* | Duplications & Deletions | Neurological system process sensory |
| Dopachrome isomerase activity | GO:0004167 | 3 | *BC036909, MIF, DDT* | Duplications | Dopachrome isomerase activity |
| Signal transducer activity | GO:0004871 | 36 | *OR11L1, OR14C36, OR2AK2, OR2G6, OR2L2, OR2L3, OR2L8, OR2M2, OR2M3, OR2M4, OR2M5, OR2M7, OR2T1, OR2T10, OR2T12, OR2T2, OR2T29, OR2T3, OR2T33, OR2T34, OR2T4, OR2T5, OR2T6, OR2T8, OR2W3, OR4A5, OR4C12, CHRNA7, IL5RA, PLXNA3, TNFRSF14, FLNA, FLRT3, PLCH2, RAF1, TOLLIP* | Duplications | Signal transducer activity transmembrane |
| Receptor activity | GO:0004872 | 35 | *OR11L1, OR14C36, OR2AK2, OR2G6, OR2L2, OR2L3, OR2L8, OR2M2, OR2M3, OR2M4, OR2M5, OR2M7, OR2T1, OR2T10, OR2T12, OR2T2, OR2T29, OR2T3, OR2T33, OR2T34, OR2T4, OR2T5, OR2T6, OR2T8, OR2W3, OR4A5, OR4C12, CHRNA7, IL5RA, PLXNA3, TNFRSF14, BTLA, ERAP1, FLJ00268, NRXN1* | Duplications & Deletions | Neurological system process sensory |
| Transmembrane signaling receptor activity | GO:0004888 | 31 | *OR11L1, OR14C36, OR2AK2, OR2G6, OR2L2, OR2L3, OR2L8, OR2M2, OR2M3, OR2M4, OR2M5, OR2M7, OR2T1, OR2T10, OR2T12, OR2T2, OR2T29, OR2T3, OR2T33, OR2T34, OR2T4, OR2T5, OR2T6, OR2T8, OR2W3, OR4A5, OR4C12, CHRNA7, IL5RA, PLXNA3, TNFRSF14* | Duplications | Signal transducer activity transmembrane |
| G-protein coupled receptor activity | GO:0004930 | 27 | *OR11L1, OR14C36, OR2AK2, OR2G6, OR2L2, OR2L3, OR2L8, OR2M2, OR2M3, OR2M4, OR2M5, OR2M7, OR2T1, OR2T10, OR2T12, OR2T2, OR2T29, OR2T3, OR2T33, OR2T34, OR2T4, OR2T5, OR2T6, OR2T8, OR2W3, OR4A5, OR4C12* | Duplications | Signaling G-Protein Coupled GPCR |
| Olfactory receptor activity | GO:0004984 | 27 | *OR11L1, OR14C36, OR2AK2, OR2G6, OR2L2, OR2L3, OR2L8, OR2M2, OR2M3, OR2M4, OR2M5, OR2M7, OR2T1, OR2T10, OR2T12, OR2T2, OR2T29, OR2T3, OR2T33, OR2T34, OR2T4, OR2T5, OR2T6, OR2T8, OR2W3, OR4A5, OR4C12* | Duplications | Genes involved olfactory pathway |
| Cell surface receptor linked signaling pathway | GO:0007166 | 38 | *OR11L1, OR14C36, OR2AK2, OR2G6, OR2L2, OR2L3, OR2L8, OR2M2, OR2M3, OR2M4, OR2M5, OR2M7, HES5, OR2T1, OR2T10, OR2T12, OR2T2, OR2T29, OR2T3, OR2T33, OR2T34, OR2T4, OR2T5, OR2T6, OR2T8, OR2W3, OR4A5, OR4C12, BC036909, MIF, IL5RA, PLXNA3, TNFRSF14, FLNA, RAF1, BTLA, CNTN6, SOX4* | Duplications | Signaling G-Protein Coupled GPCR |
| G-protein coupled receptor protein signaling pathway | GO:0007186 | 28 | *OR11L1, OR14C36, OR2AK2, OR2G6, OR2L2, OR2L3, OR2L8, OR2M2, OR2M3, OR2M4, OR2M5, OR2M7, OR2T1, OR2T10, OR2T12, OR2T2, OR2T29, OR2T3, OR2T33, OR2T34, OR2T4, OR2T5, OR2T6, OR2T8, OR2W3, OR4A5, OR4C12, FLNA* | Duplications | Signaling G-Protein Coupled GPCR |
| Sensory perception | GO:0007600 | 29 | *OR11L1, OR14C36, OR2AK2, OR2G6, OR2L2, OR2L3, OR2L8, OR2M2, OR2M3, OR2M4, OR2M5, OR2M7, OR2T1, OR2T10, OR2T12, OR2T2, OR2T29, OR2T3, OR2T33, OR2T34, OR2T4, OR2T5, OR2T6, OR2T8, OR2W3, OR4A5, OR4C12, NIPBL, FSCN2* | Duplications | Neurological system process sensory |
| Sensory perception of chemical stimulus | GO:0007606 | 27 | *OR11L1, OR14C36, OR2AK2, OR2G6, OR2L2, OR2L3, OR2L8, OR2M2, OR2M3, OR2M4, OR2M5, OR2M7, OR2T1, OR2T10, OR2T12, OR2T2, OR2T29, OR2T3, OR2T33, OR2T34, OR2T4, OR2T5, OR2T6, OR2T8, OR2W3, OR4A5, OR4C12* | Duplications | Neurological system process sensory |
| Sensory perception of smell | GO:0007608 | 27 | *OR11L1, OR14C36, OR2AK2, OR2G6, OR2L2, OR2L3, OR2L8, OR2M2, OR2M3, OR2M4, OR2M5, OR2M7, OR2T1, OR2T10, OR2T12, OR2T2, OR2T29, OR2T3, OR2T33, OR2T34, OR2T4, OR2T5, OR2T6, OR2T8, OR2W3, OR4A5, OR4C12* | Duplications | Neurological system process sensory |
| Detection of chemical stimulus | GO:0009593 | 27 | *OR11L1, OR14C36, OR2AK2, OR2G6, OR2L2, OR2L3, OR2L8, OR2M2, OR2M3, OR2M4, OR2M5, OR2M7, OR2T1, OR2T10, OR2T12, OR2T2, OR2T29, OR2T3, OR2T33, OR2T34, OR2T4, OR2T5, OR2T6, OR2T8, OR2W3, OR4A5, OR4C12* | Duplications | Detection stimulus sensory perception |
| Signaling receptor activity | GO:0038023 | 31 | *OR11L1, OR14C36, OR2AK2, OR2G6, OR2L2, OR2L3, OR2L8, OR2M2, OR2M3, OR2M4, OR2M5, OR2M7, OR2T1, OR2T10, OR2T12, OR2T2, OR2T29, OR2T3, OR2T33, OR2T34, OR2T4, OR2T5, OR2T6, OR2T8, OR2W3, OR4A5, OR4C12, CHRNA7, IL5RA, PLXNA3, TNFRSF14* | Duplications | Signal transducer activity transmembrane |
| Response to chemical stimulus | GO:0042221 | 47 | *OR11L1, OR14C36, OR2AK2, OR2G6, OR2L2, OR2L3, OR2L8, OR2M2, OR2M3, OR2M4, OR2M5, OR2M7, OR2T1, OR2T10, EMD, OR2T12, NDUFS4, OR2T2, OR2T29, OR2T3, OR2T33, OR2T34, OR2T4, OR2T5, OR2T6, OR2T8, OR2W3, ADNP2, OR4A5, OR4C12, BC036909, MIF, CHRNA7, IL5RA, PLXNA3, TNFRSF14, GDNF, DERL3, RAF1, NRXN1, CNTN4, CNTN6, CYP2E1, SOX4, ATP7B, PAOX, ACTG1* | Duplications & Deletions | Neurological system process sensory |
| Phenylpyruvate tautomerase activity | GO:0050178 | 2 | *BC036909, MIF* | Duplications | Phenylpyruvate tautomerase activity |
| Neurological system process | GO:0050877 | 34 | *OR11L1, OR14C36, OR2AK2, OR2G6, OR2L2, KCNG2, OR2L3, OR2L8, OR2M2, OR2M3, OR2M4, OR2M5, OR2M7, HES5, OR2T1, OR2T10, OR2T12, OR2T2, OR2T29, OR2T3, OR2T33, OR2T34, OR2T4, OR2T5, OR2T6, OR2T8, OR2W3, OR4A5, OR4C12, CHRNA7, RAF1, NRXN1, NIPBL, FSCN2* | Duplications & Deletions | Neurological system process sensory |
| Detection of stimulus involved in sensory perception | GO:0050906 | 27 | *OR11L1, OR14C36, OR2AK2, OR2G6, OR2L2, OR2L3, OR2L8, OR2M2, OR2M3, OR2M4, OR2M5, OR2M7, OR2T1, OR2T10, OR2T12, OR2T2, OR2T29, OR2T3, OR2T33, OR2T34, OR2T4, OR2T5, OR2T6, OR2T8, OR2W3, OR4A5, OR4C12* | Duplications | Detection stimulus sensory perception |
| Detection of chemical stimulus involved in sensory perception | GO:0050907 | 27 | *OR11L1, OR14C36, OR2AK2, OR2G6, OR2L2, OR2L3, OR2L8, OR2M2, OR2M3, OR2M4, OR2M5, OR2M7, OR2T1, OR2T10, OR2T12, OR2T2, OR2T29, OR2T3, OR2T33, OR2T34, OR2T4, OR2T5, OR2T6, OR2T8, OR2W3, OR4A5, OR4C12* | Duplications | Detection stimulus sensory perception |
| Detection of chemical stimulus involved in sensory perception of smell | GO:0050911 | 27 | *OR11L1, OR14C36, OR2AK2, OR2G6, OR2L2, OR2L3, OR2L8, OR2M2, OR2M3, OR2M4, OR2M5, OR2M7, OR2T1, OR2T10, OR2T12, OR2T2, OR2T29, OR2T3, OR2T33, OR2T34, OR2T4, OR2T5, OR2T6, OR2T8, OR2W3, OR4A5, OR4C12* | Duplications | Detection stimulus sensory perception |
| Detection of stimulus | GO:0051606 | 28 | *OR11L1, OR14C36, OR2AK2, OR2G6, OR2L2, OR2L3, OR2L8, OR2M2, OR2M3, OR2M4, OR2M5, OR2M7, OR2T1, OR2T10, OR2T12, OR2T2, OR2T29, OR2T3, OR2T33, OR2T34, OR2T4, OR2T5, OR2T6, OR2T8, OR2W3, OR4A5, OR4C12, SOX4* | Duplications | Detection stimulus sensory perception |
| Olfactory transduction | M14091 | 27 | *OR11L1, OR14C36, OR2AK2, OR2G6, OR2L2, OR2L3, OR2L8, OR2M2, OR2M3, OR2M4, OR2M5, OR2M7, OR2T1, OR2T10, OR2T12, OR2T2, OR2T29, OR2T3, OR2T33, OR2T34, OR2T4, OR2T5, OR2T6, OR2T8, OR2W3, OR4A5, OR4C12* | Duplications | Genes involved olfactory pathway |
| Genes involved in Olfactory Signaling Pathway | R-HSA-381753 | 26 | *OR14C36, OR2AK2, OR2G6, OR2L2, OR2L3, OR2L8, OR2M2, OR2M3, OR2M4, OR2M5, OR2M7, OR2T1, OR2T10, OR2T12, OR2T2, OR2T29, OR2T3, OR2T33, OR2T34, OR2T4, OR2T5, OR2T6, OR2T8, OR2W3, OR4A5, OR4C12* | Duplications | Genes involved olfactory pathway |
| ^1^ Genes could fall in more than one gene set, so gene-sets are not necessarily independent | | | | | |

**Supplementary Figure 1**: Quality control flowchart steps for selection of samples and CNVs


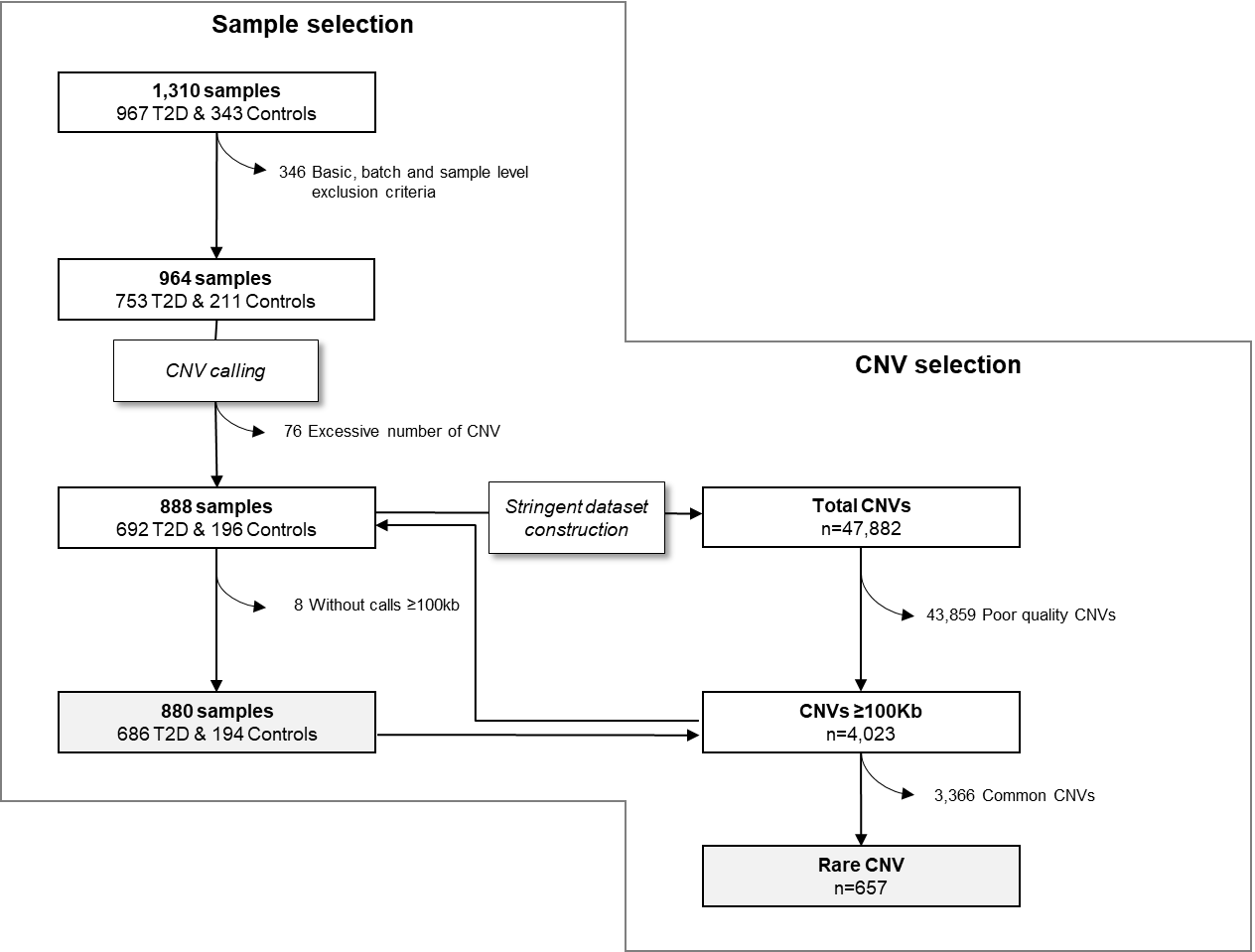


**Supplementary Figure 2**: Sample batch level and sample level quality control measures thresholds used to select samples


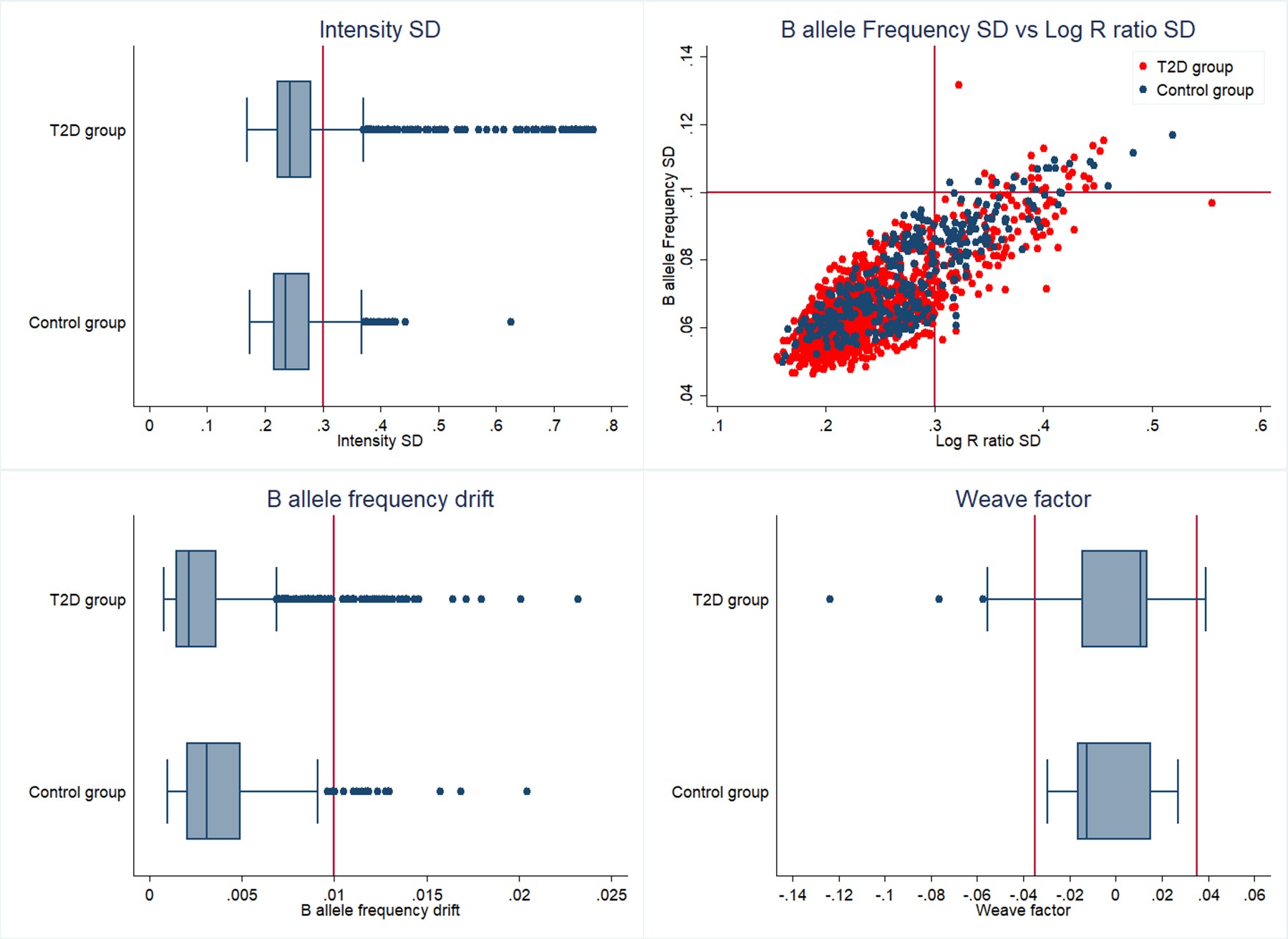


**Supplementary Figure 3**: Correlation between the number of calls obtained by iPattern, Birdsuite and PennCNV algorithms.

Correlation between the number of calls ≥ 5 probes and ≥ 5kb length per sample obtained by iPattern, Birdsuite and PennCNV algorithms in the samples that passed the QC criteria. The Pearson correlation coefficient is shown. Figures at the top and at the bottom represent the samples before (753 T2D samples and 211 control samples) and after using the percentile 95 numbers of calls per sample through the algorithms cutoff (692 T2D samples and 196 control samples).


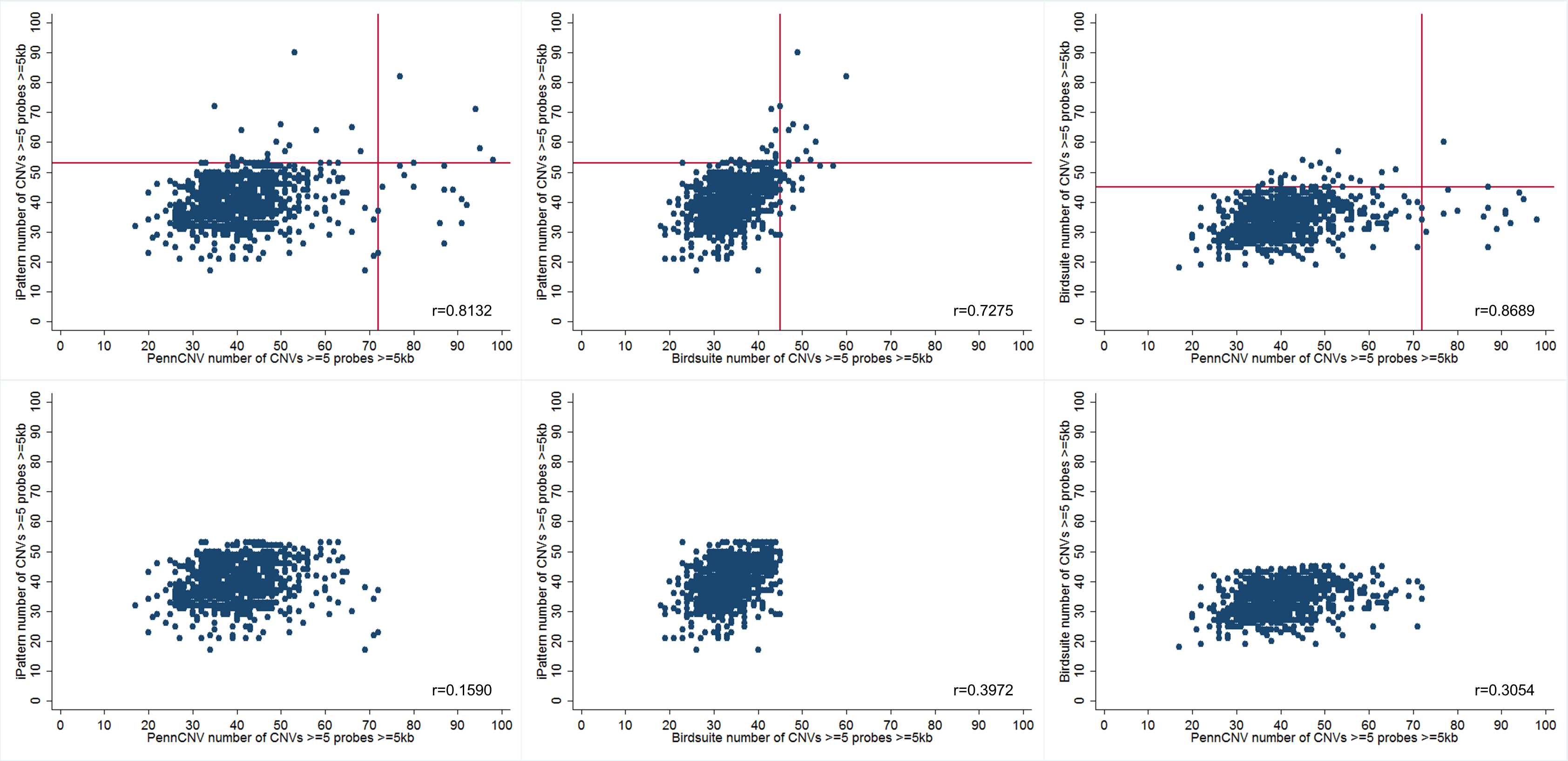


**Supplementary Figure 4**: Distribution of age, body mass index, total cholesterol, triglycerides, MDS1 and MDS-2 ancestry vectors, CNVs size and Genic CNVs size in T2D and control groups

**
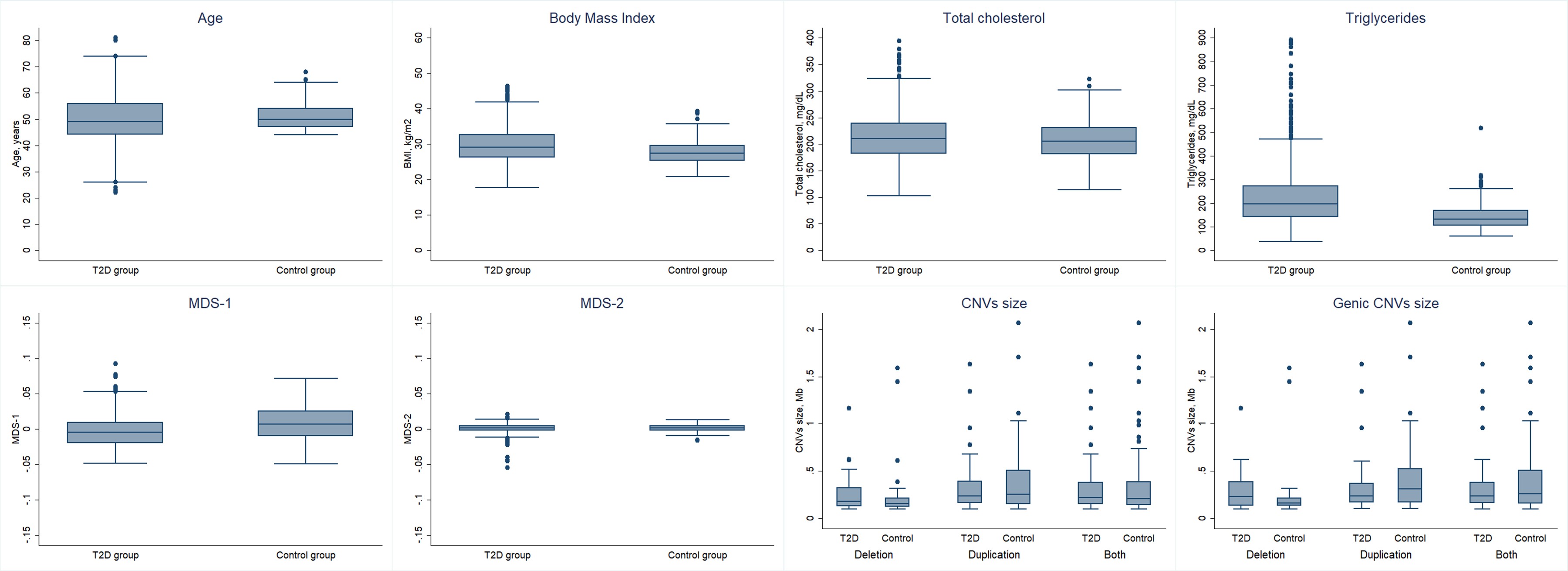
**

**Supplementary Figure 5**: Multidimensional Scaling (MDS) plot showing the ancestry distribution in the T2D and control groups


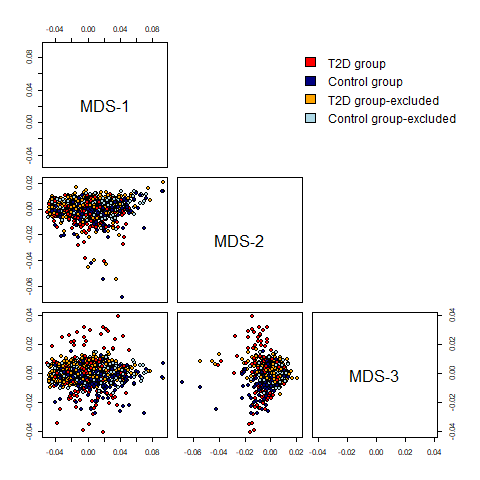


**Supplementary Figure 6**: Representative plots of CNV regions associated with T2D and rare CNVs diabetes candidate genes.

| **Region** | **Duplication** | **Deletion** | **Normal Copy Number** |
| --- | --- | --- | --- |
| ***ZNF718*** | 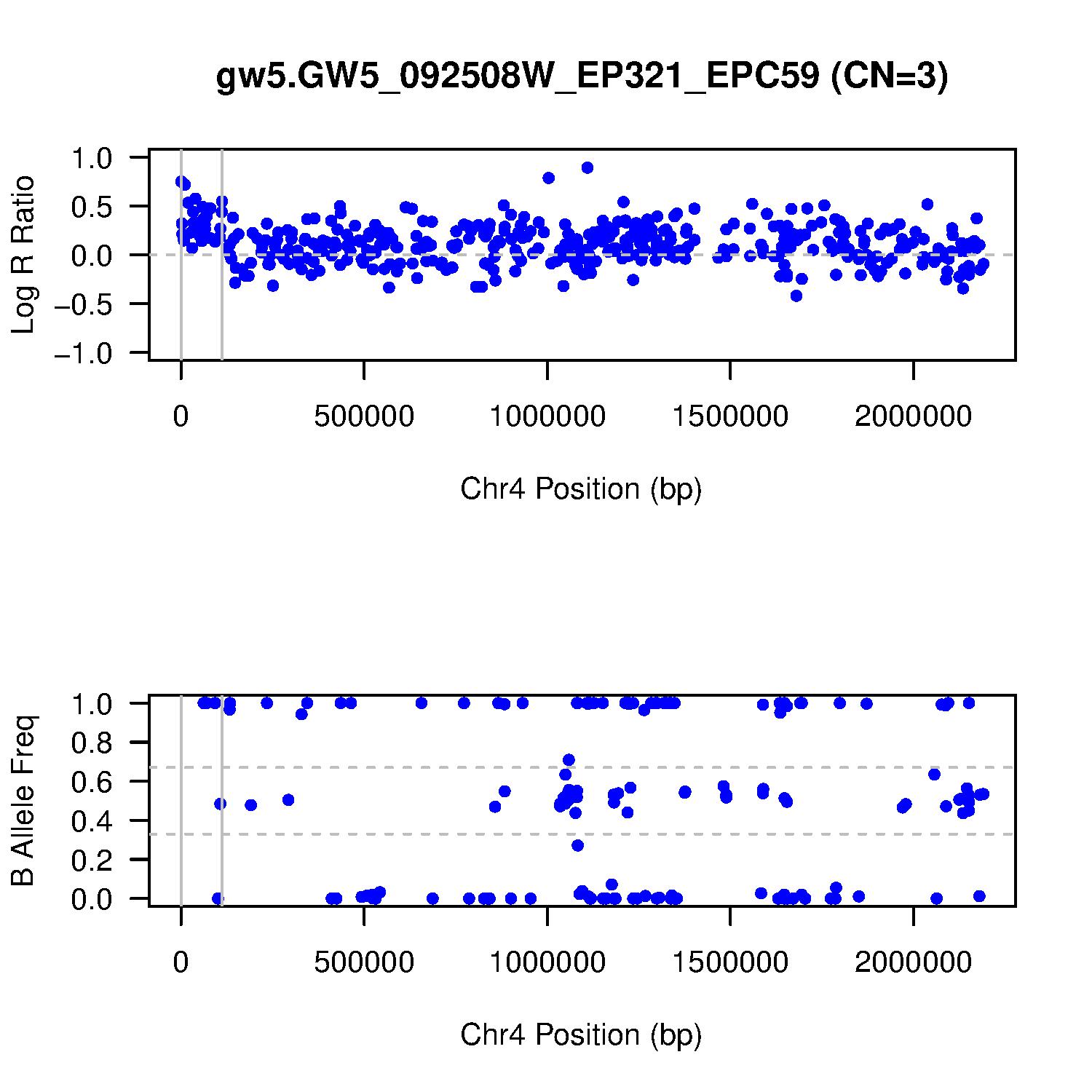 | 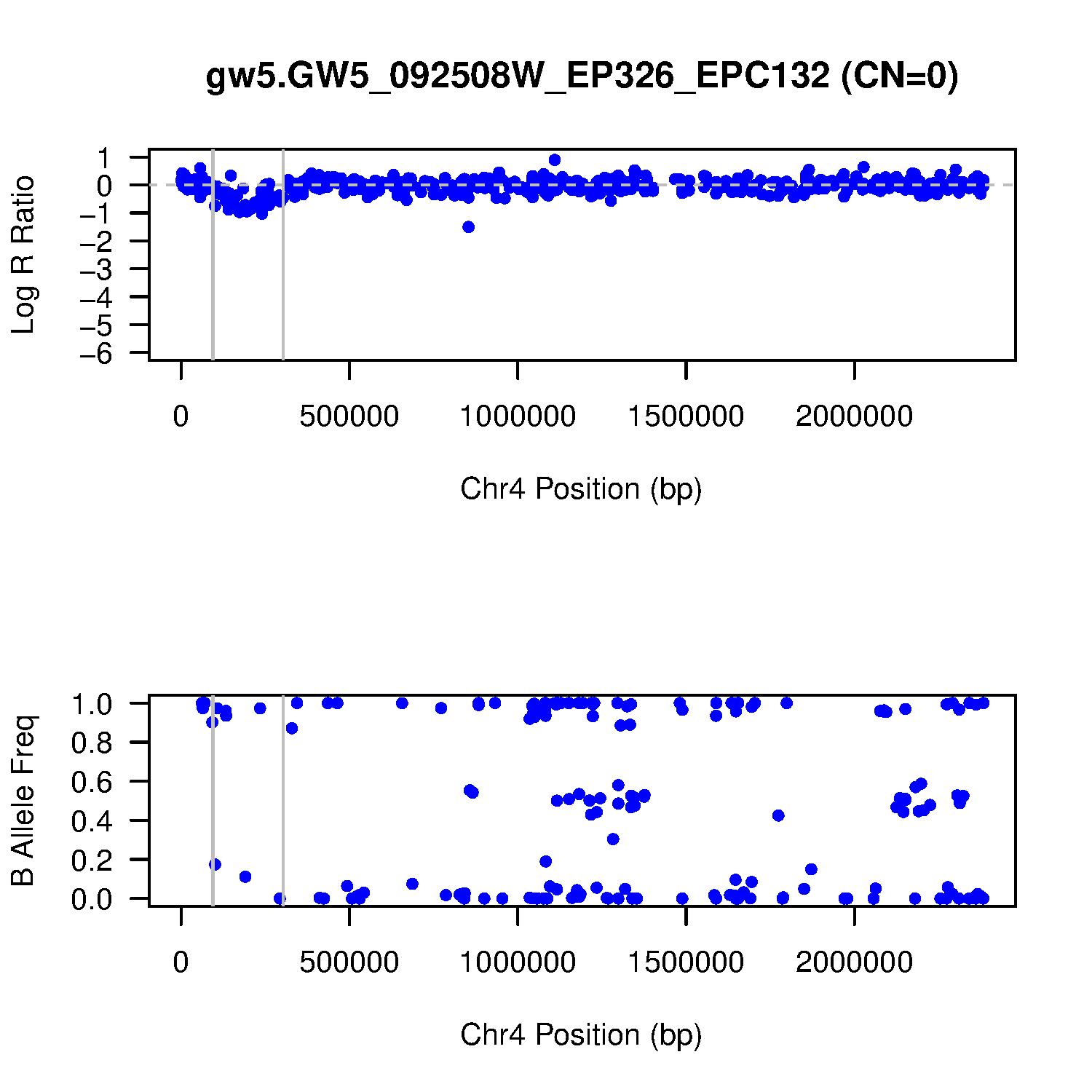 | 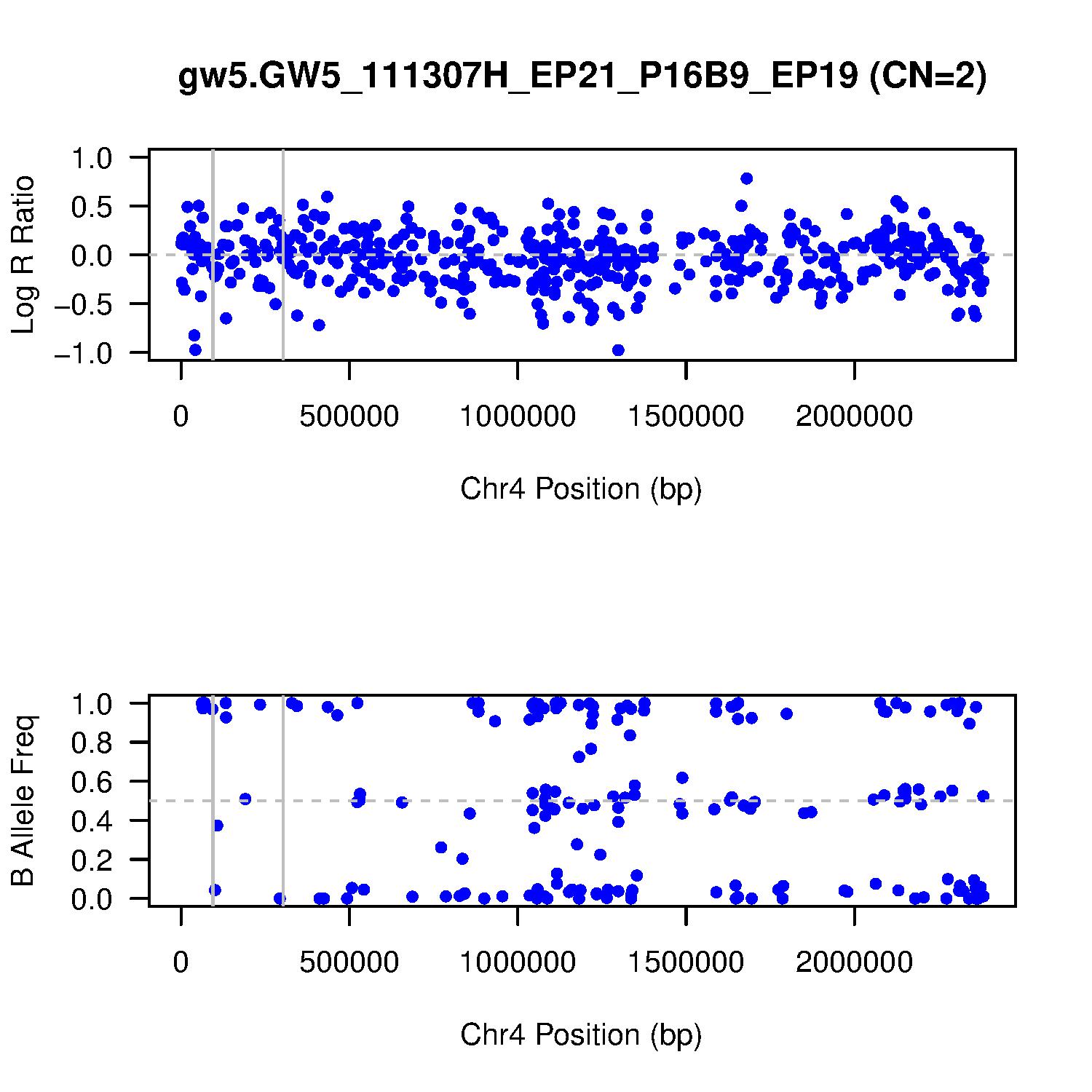 |
| ***AMY2B*** | 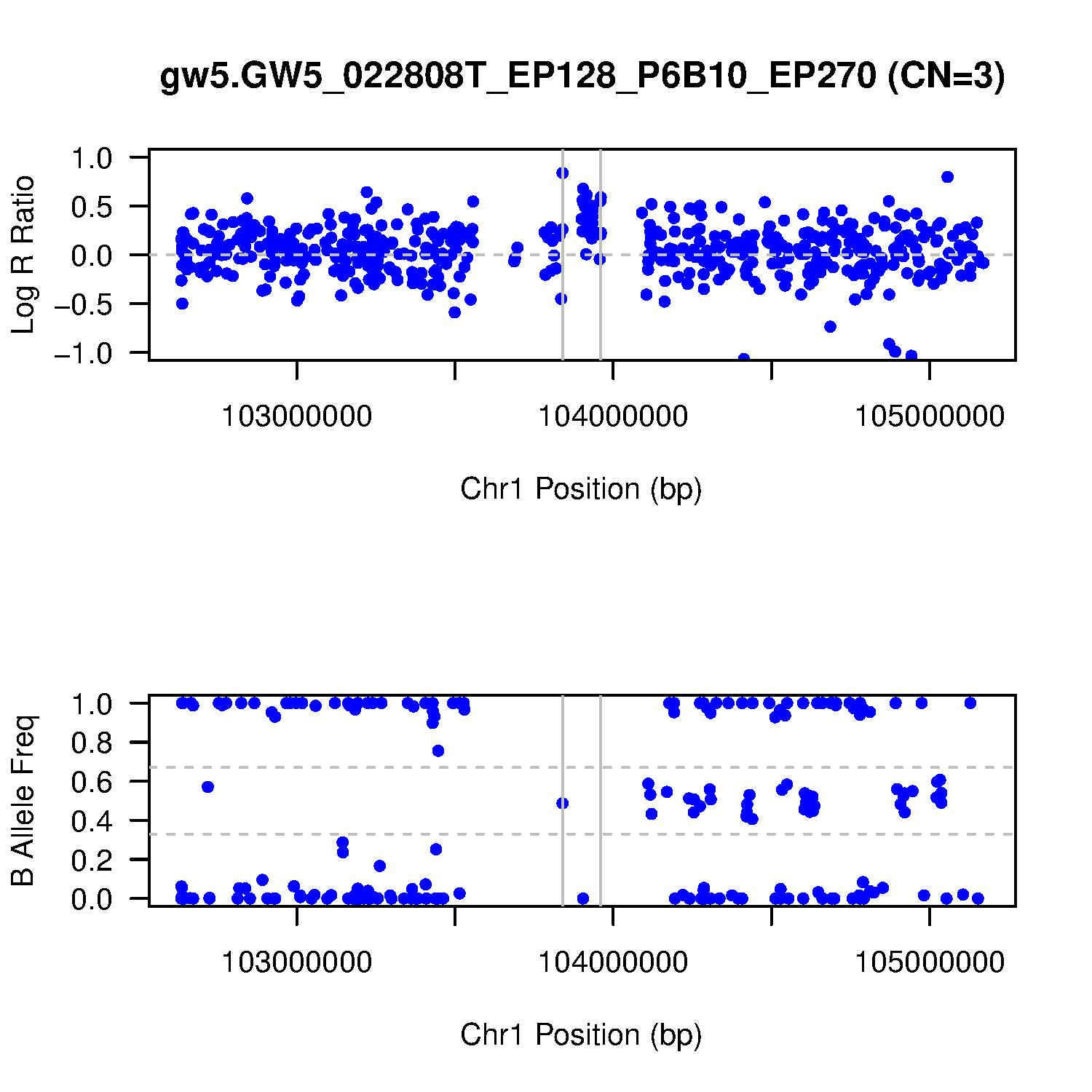 | Not detected | 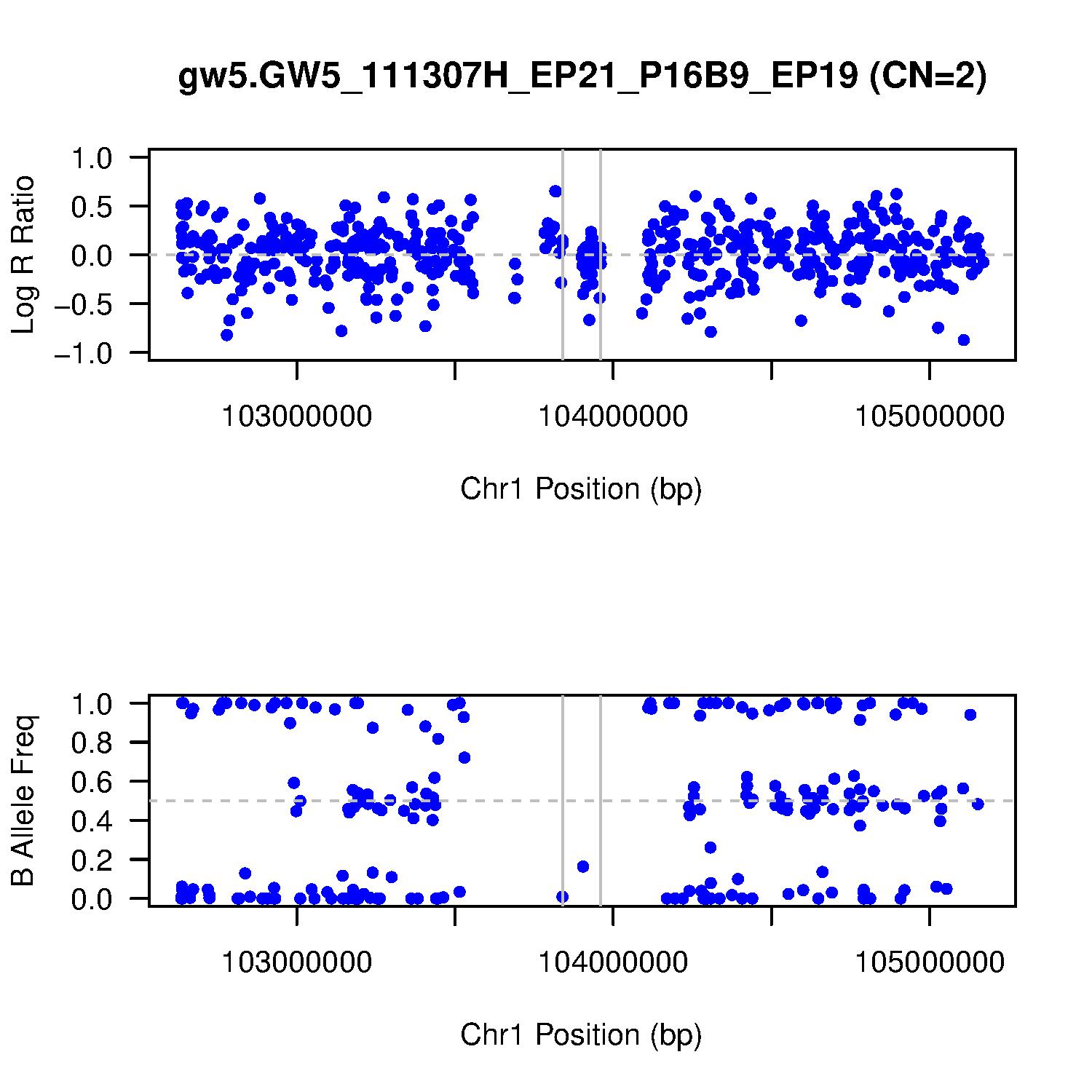 |
| ***LPP*** | 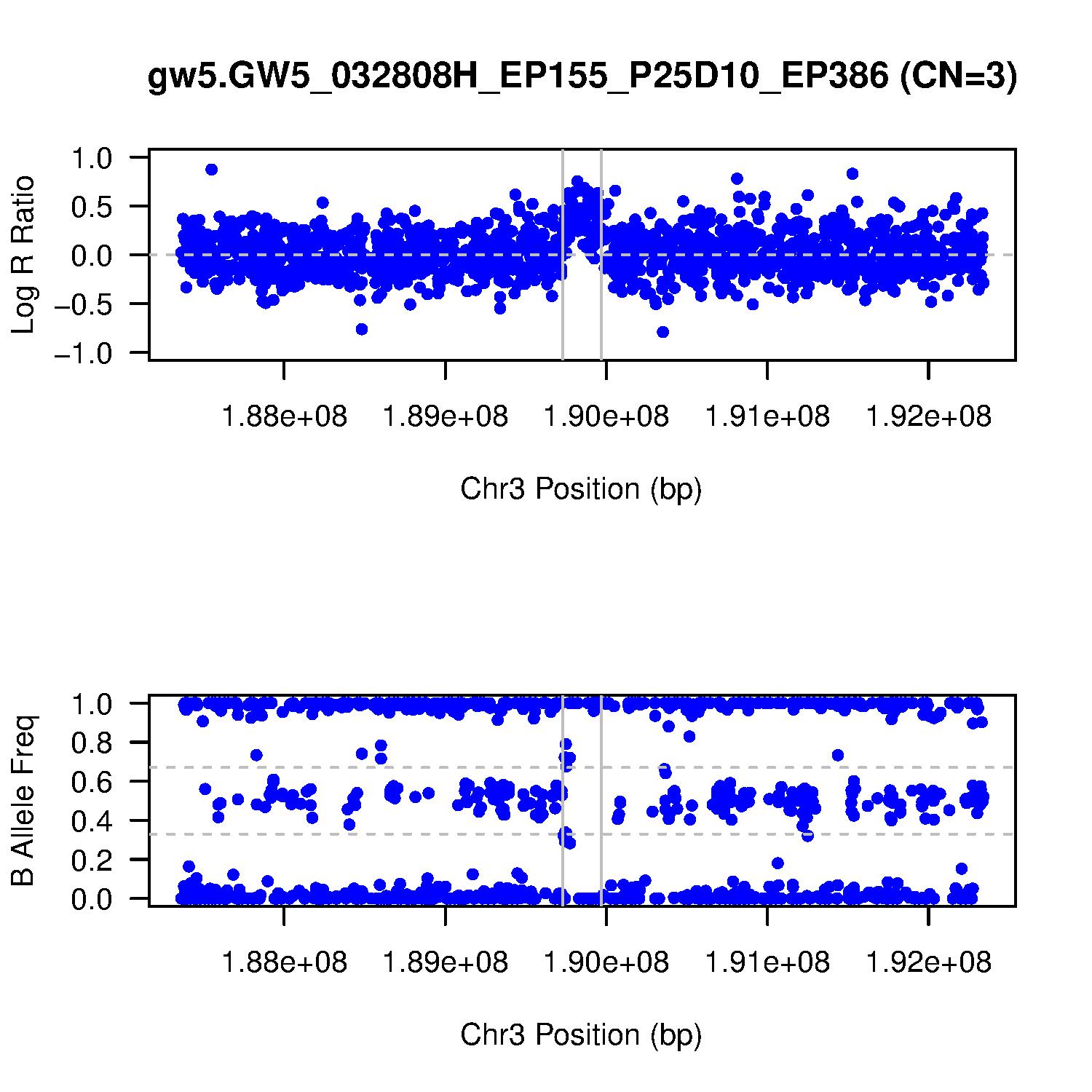 | Not detected | 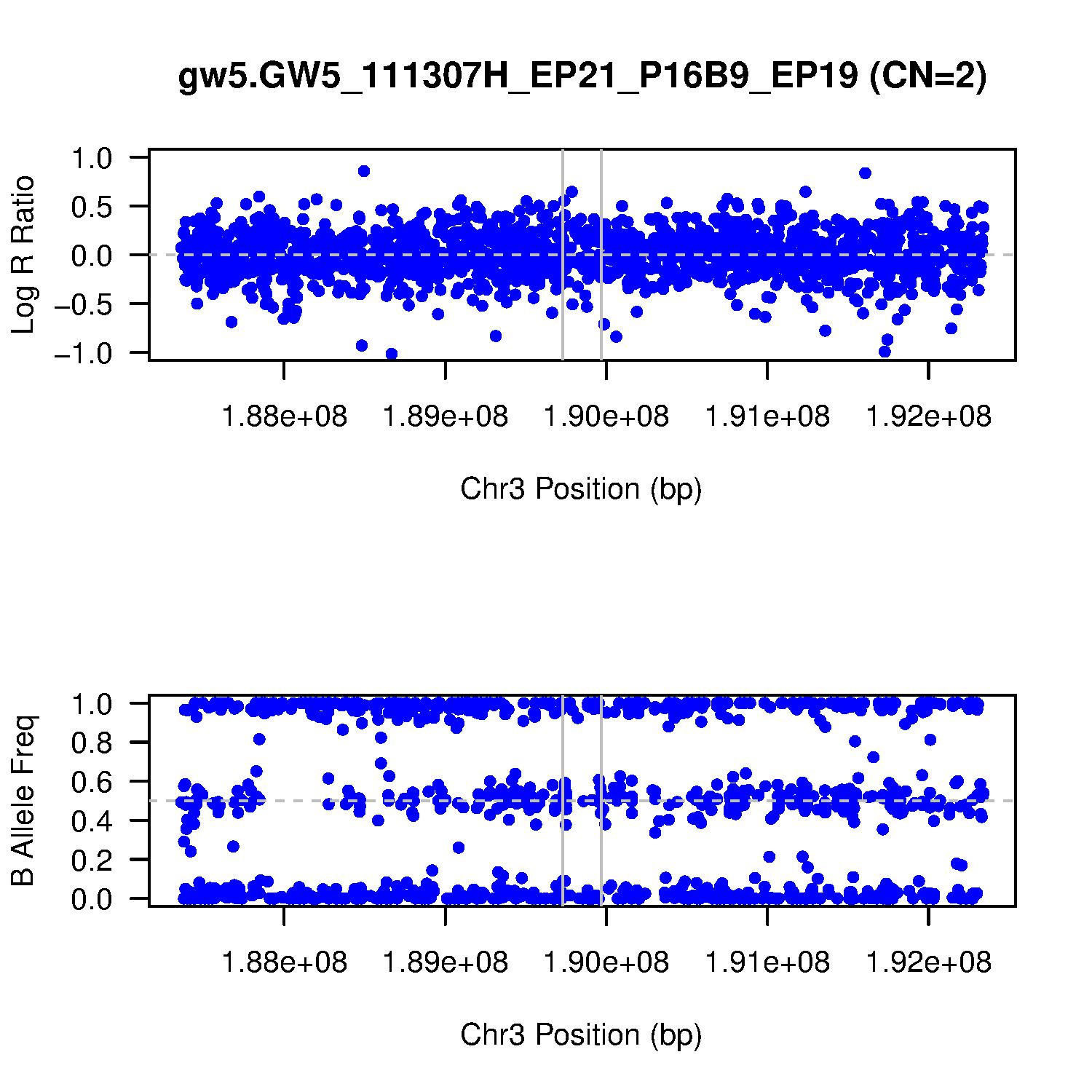 |
| ***ARL15*** | 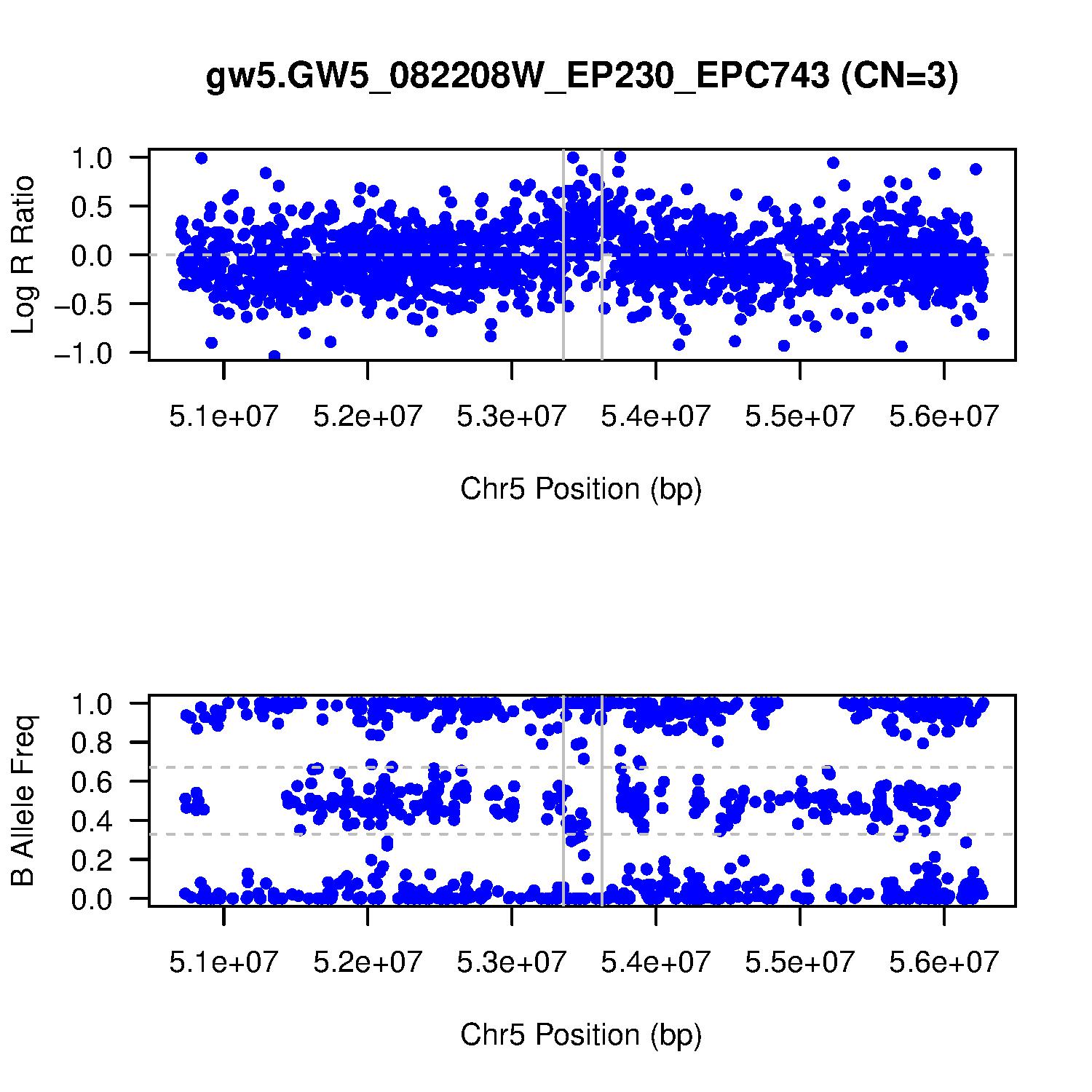 | Not detected | 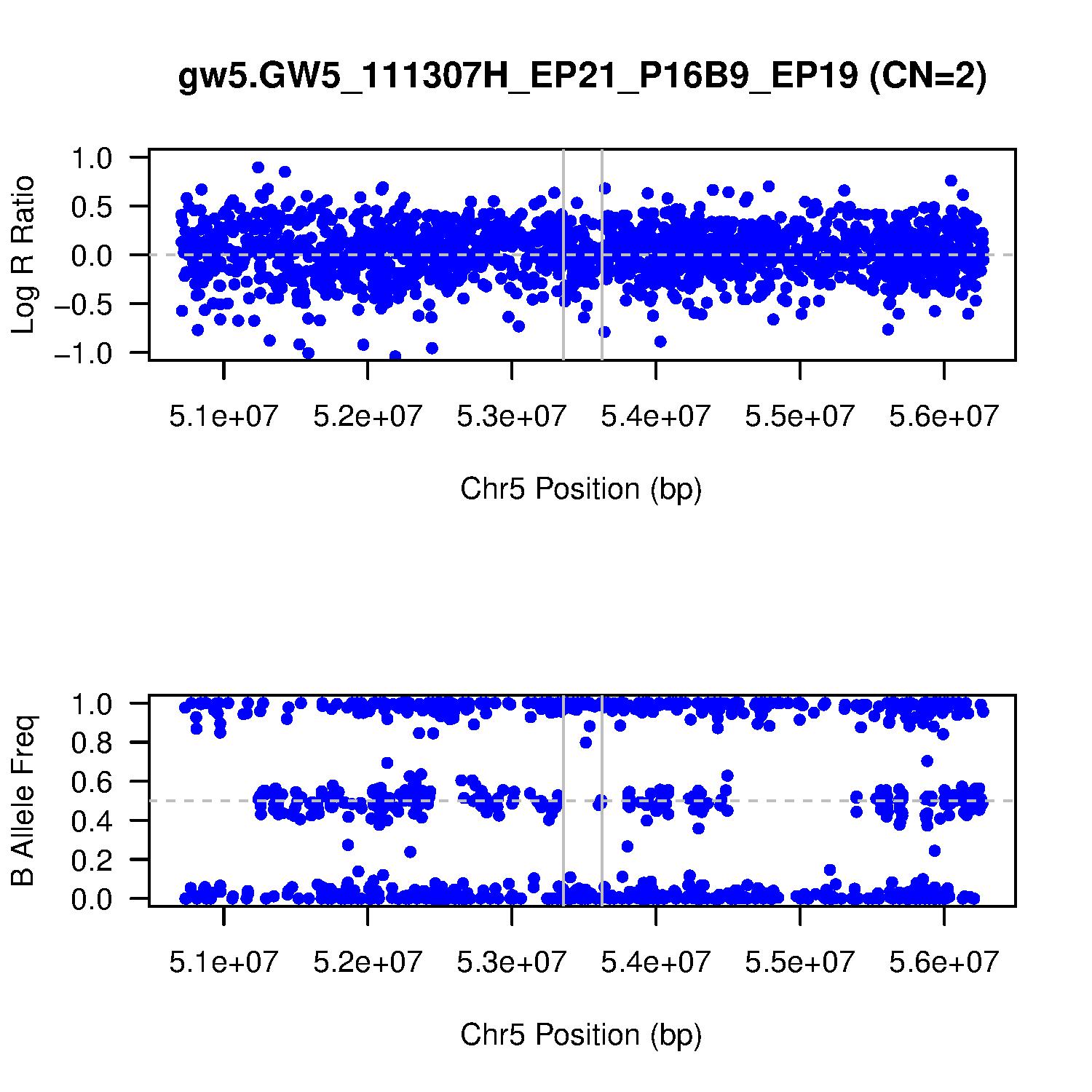 |
| ***HFE*** | Not detected | 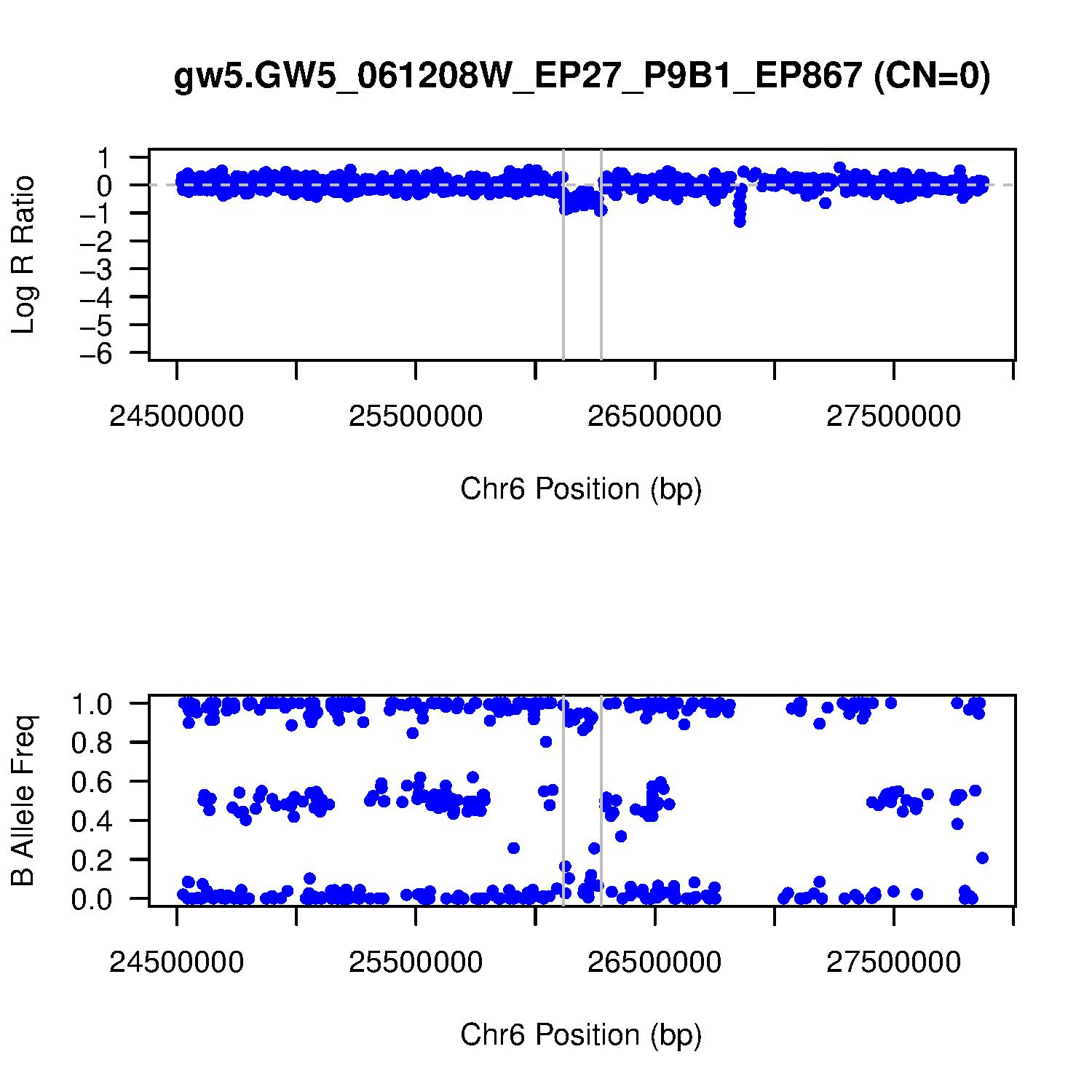 | 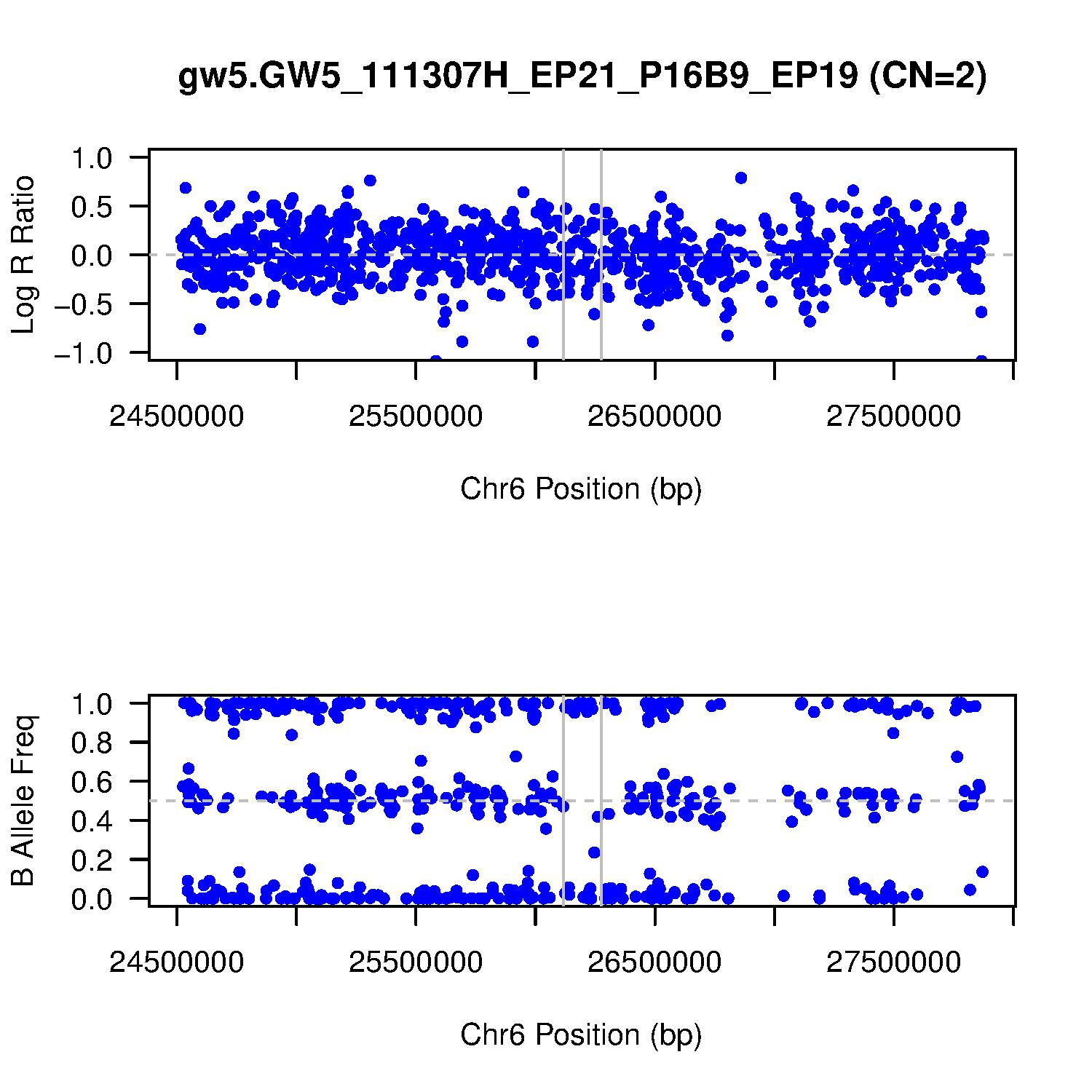 |
| ***CDKAL1*** | Not detected | 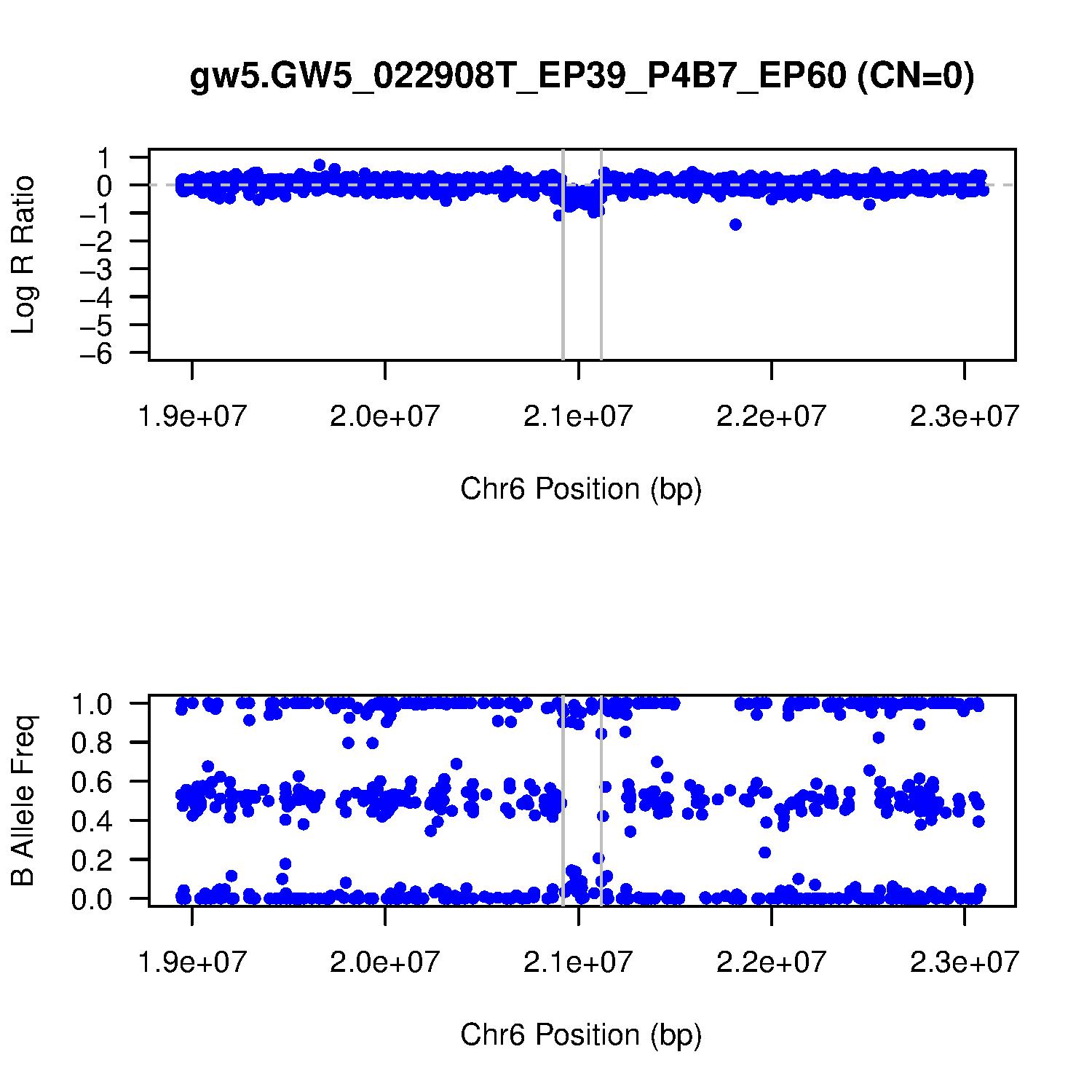 | 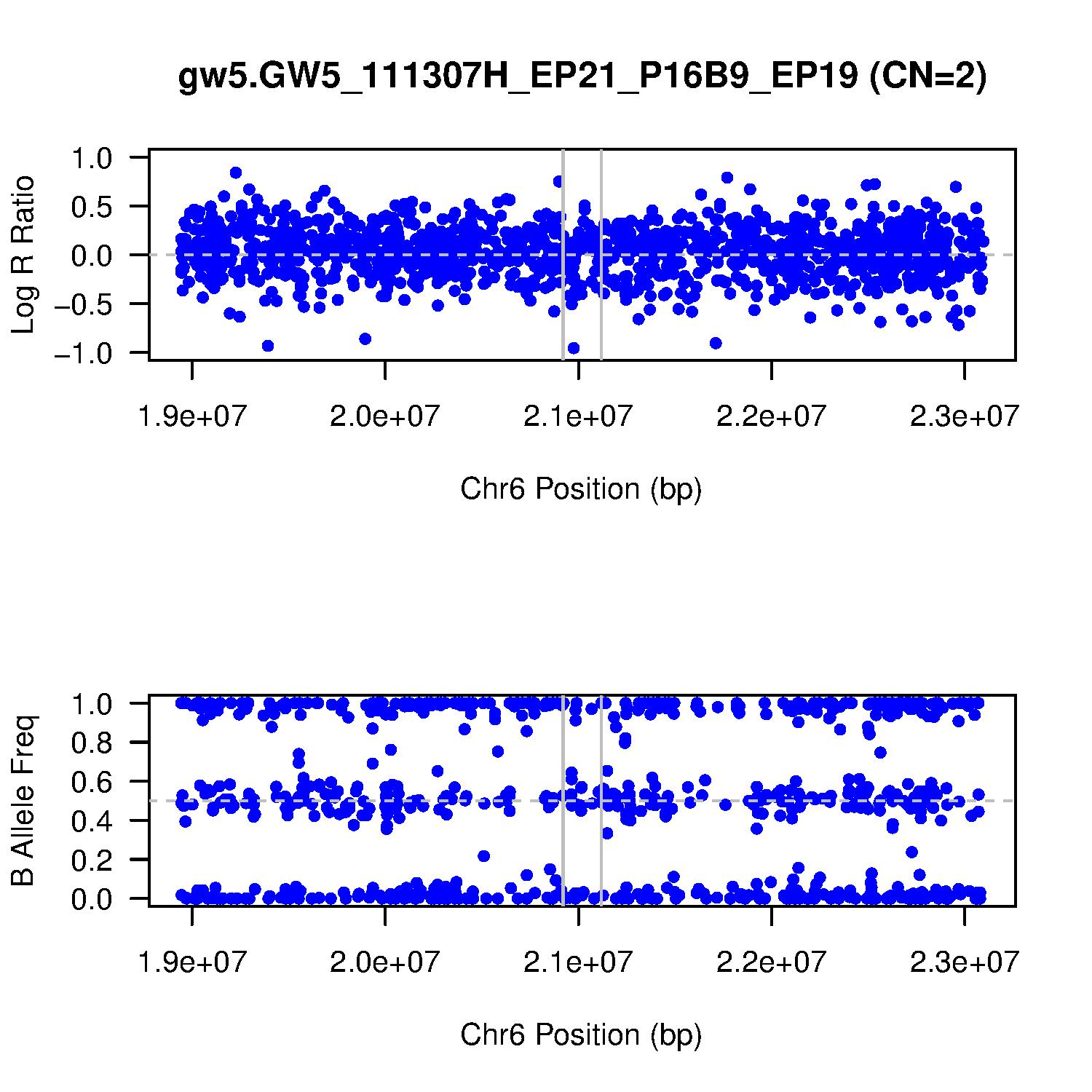 |
| ***RASGRP1*** | 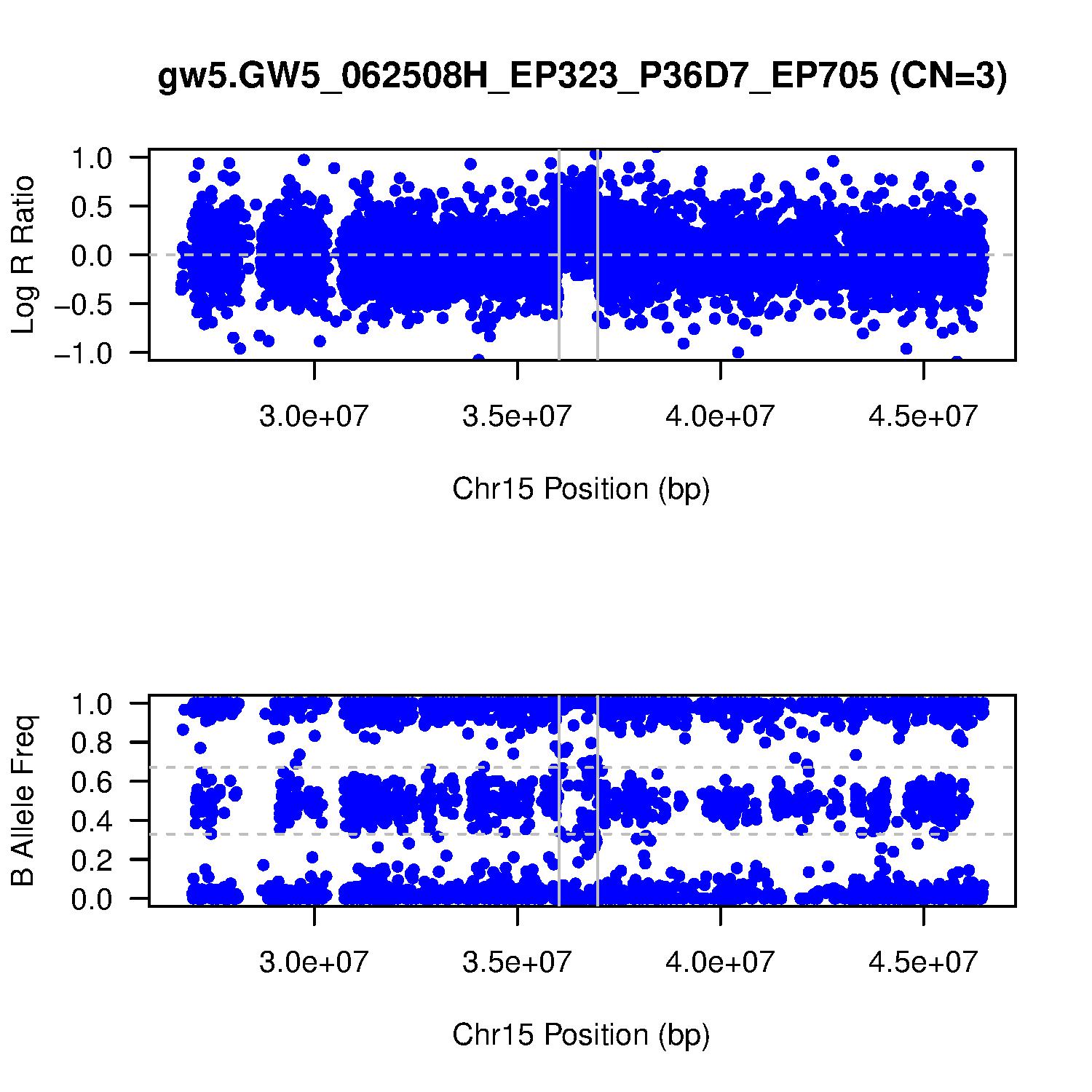 | Not detected | 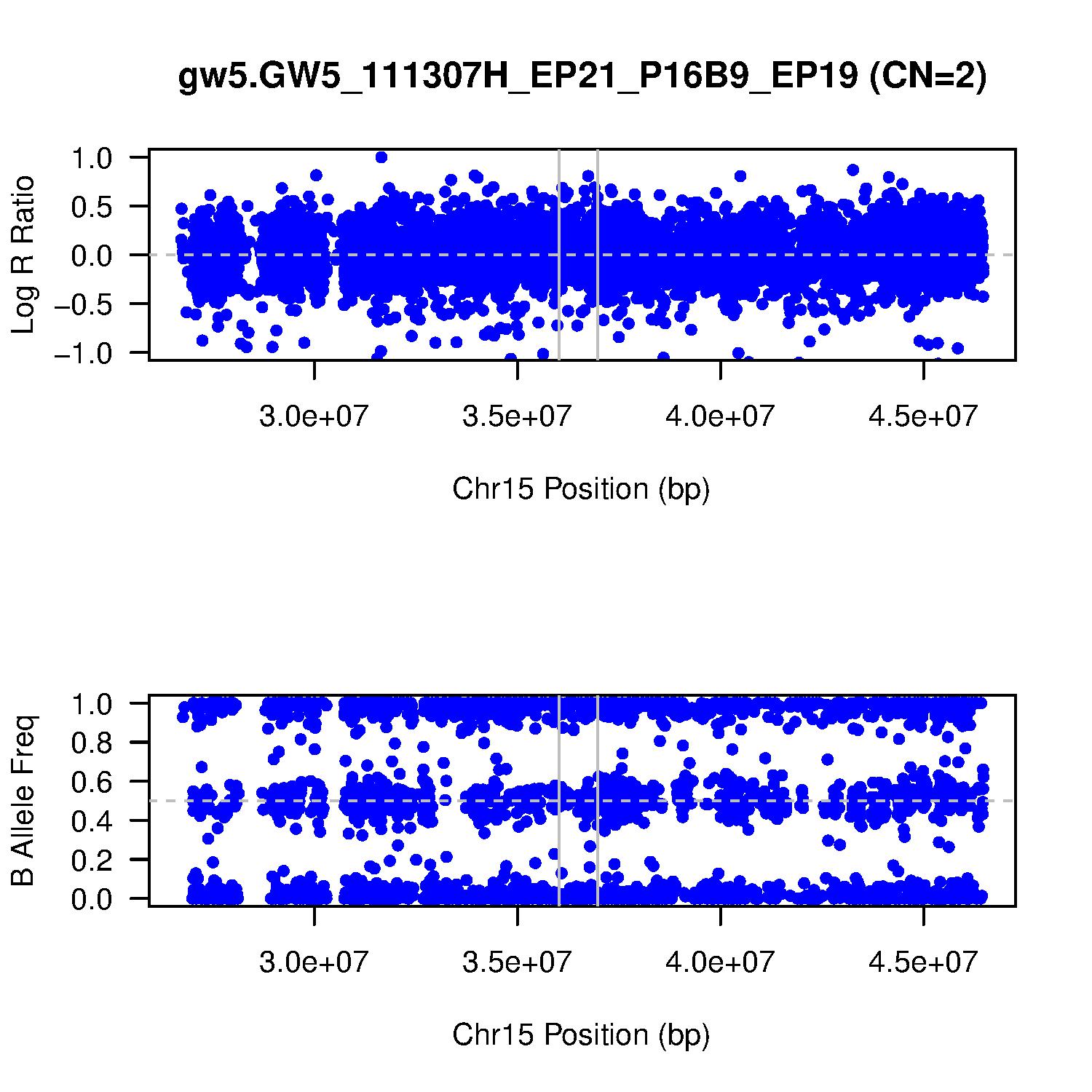 |

**References for Supplementary Table 3:**

- Agarwal AK, Arioglu E, De Almeida S, Akkoc N, Taylor SI, Bowcock AM, Barnes RI, Garg A. AGPAT2 is mutated in congenital generalized lipodystrophy linked to chromosome 9q34. Nat Genet 2002;31:21-23
- Altshuler D, Hirschhorn JN, Klannemark M, Lindgren CM, Vohl MC, Nemesh J, Lane CR, Schaffner SF, Bolk S, Brewer C, Tuomi T, Gaudet D, Hudson TJ, Daly M, Groop L, Lander ES. The common PPARgamma Pro12Ala polymorphism is associated with decreased risk of type 2 diabetes. Nat Genet 2000;26:76-80
- Babenko AP, Polak M, CavŽ H, Busiah K, Czernichow P, Scharfmann R, Bryan J, Aguilar-Bryan L, Vaxillaire M, Froguel P. Activating mutations in the ABCC8 gene in neonatal diabetes mellitus. N Engl J Med 2006;355:456-466
- Bae JS, Cheong HS, Kim JH, Park BL, Kim JH, Park TJ, Kim JY, Pasaje CF, Lee JS, Park YJ, Park M, Park C, Koh I, Chung YJ, Lee JY, Shin HD. The genetic effect of copy number variations on the risk of type 2 diabetes in a Korean population. PLoS One 2011;6:e19091
- Bailey JN, Lu L, Chou JW, Xu J, McWilliams DR, Howard TD, Freedman BI, Bowden DW, Langefeld CD, Palmer ND. The Role of Copy Number Variation in African Americans with Type 2 Diabetes-Associated End Stage Renal Disease. J Mol Genet Med 2013 Jul 31;7:61.
- Barroso I, Luan J, Middelberg RP, Harding AH, Franks PW, Jakes RW, Clayton D, Schafer AJ, O'Rahilly S, Wareham NJ. Candidate gene association study in type 2 diabetes indicates a role for genes involved in beta-cell function as well as insulin action. PLoS Biol 2003;1:E20
- Below JE, Gamazon ER, Morrison JV, Konkashbaev A, Pluzhnikov A, McKeigue PM, Parra EJ, Elbein SC, Hallman DM, Nicolae DL, Bell GI, Cruz M, Cox NJ, Hanis CL. Genome-wide association and meta-analysis in populations from Starr County, Texas, and Mexico City identify type 2 diabetes susceptibility loci and enrichment for expression quantitative trait loci in top signals. Diabetologia 2011;54(8):2047-2055
- Bouatia-Naji N, Bonnefond A, Cavalcanti-Proença C, Sparsø T, Holmkvist J, Marchand M, Delplanque J, Lobbens S, Rocheleau G, Durand E, De Graeve F, Chèvre JC, Borch-Johnsen K, Hartikainen AL, Ruokonen A, Tichet J, Marre M, Weill J, Heude B, Tauber M, Lemaire K, Schuit F, Elliott P, Jørgensen T, Charpentier G, Hadjadj S, Cauchi S, Vaxillaire M, Sladek R, Visvikis-Siest S, Balkau B, Lévy-Marchal C, Pattou F, Meyre D, Blakemore AI, Jarvelin MR, Walley AJ, Hansen T, Dina C, Pedersen O, Froguel P. A variant near MTNR1B is associated with increased fasting plasma glucose levels and type 2 diabetes risk. Nat Genet 2009;41:89-94
- Brickwood S, Bonthron DT, Al-Gazali LI, Piper K, Hearn T, Wilson DI, Hanley NA. Wolcott-Rallison syndrome: pathogenic insights into neonatal diabetes from new mutation and expression studies of EIF2AK3. J Med Genet 2003;40:685-689
- Cameron EA, Martinez-Marignac VL, Chan A, Valladares A, Simmonds LV, Wacher N, Kumate J, McKeigue P, Shriver MD, Kittles R, Cruz M, Parra EJ. MGEA5-14 polymorphism and type 2 diabetes in Mexico City. Am J Hum Biol 2007;19:593-596
- Campuzano V, Montermini L, Molt˜ MD, Pianese L, CossŽe M, Cavalcanti F, Monros E, Rodius F, Duclos F, Monticelli A, Zara F, Ca–izares J, Koutnikova H, Bidichandani SI, Gellera C, Brice A, Trouillas P, De Michele G, Filla A, De Frutos R, Palau F, Patel PI, Di Donato S, Mandel JL, Cocozza S, Koenig M, Pandolfo M. Friedreich's ataxia: autosomal recessive disease caused by an intronic GAA triplet repeat expansion. Science 1996;271:1423-1427
- Chen X, Li X, Wang P, Liu Y, Zhang Z, Zhao G, Xu H, Zhu J, Qin X, Chen S, Hu L, Kong X. Novel association strategy with copy number variation for identifying new risk Loci of human diseases. PLoS One 2010;5:e12185.
- Chen YF, Wu CY, Kirby R, Kao CH, Tsai TF. A role for the CISD2 gene in lifespan control and human disease. Ann N Y Acad Sci 2010;1201:58-64
- Cheung YH, Watkinson J, Anastassiou D. Conditional meta-analysis stratifying on detailed HLA genotypes identifies a novel type 1 diabetes locus around TCF19 in the MHC. Hum Genet 2011;129:161-176
- Chiefari E, Tanyolaç S, Paonessa F, Pullinger CR, Capula C, Iiritano S, Mazza T, Forlin M, Fusco A, Durlach V, Durlach A, Malloy MJ, Kane JP, Heiner SW, Filocamo M, Foti DP, Goldfine ID, Brunetti A. Functional variants of the HMGA1 gene and type 2 diabetes mellitus. JAMA 2011;305:903-912
- Cho YS, Chen CH, Hu C, Long J, Ong RT, Sim X, Takeuchi F, Wu Y, Go MJ, Yamauchi T, Chang YC, Kwak SH, Ma RC, Yamamoto K, Adair LS, Aung T, Cai Q, Chang LC, Chen YT, Gao Y, Hu FB, Kim HL, Kim S, Kim YJ, Lee JJ, Lee NR, Li Y, Liu JJ, Lu W, Nakamura J, Nakashima E, Ng DP, Tay WT, Tsai FJ, Wong TY, Yokota M, Zheng W, Zhang R, Wang C, So WY, Ohnaka K, Ikegami H, Hara K, Cho YM, Cho NH, Chang TJ, Bao Y, Hedman ÅK, Morris AP, McCarthy MI; DIAGRAM Consortium; MuTHER Consortium, Takayanagi R, Park KS, Jia W, Chuang LM, Chan JC, Maeda S, Kadowaki T, Lee JY, Wu JY, Teo YY, Tai ES, Shu XO, Mohlke KL, Kato N, Han BG, Seielstad M. Meta-analysis of genome-wide association studies identifies eight new loci for type 2 diabetes in east Asians. Nat Genet 2011;44:67-72
- Cruz M, Valladares-Salgado A, Garcia-Mena J, Ross K, Edwards M, Angeles-Martinez J, Ortega-Camarillo C, de la Pe–a JE, Burguete-Garcia AI, Wacher-Rodarte N, Ambriz R, Rivera R, D'artote AL, Peralta J, Parra EJ, Kumate J. Candidate gene association study conditioning on individual ancestry in patients with type 2 diabetes and metabolic syndrome from Mexico City. Diabetes Metab Res Rev 2010;26:261-270
- Dajani R, Li J, Wei Z, Glessner JT, Chang X, Cardinale CJ, Pellegrino R, Wang T, Hakooz N, Khader Y, Sheshani A, Zandaki D, Hakonarson H. CNV Analysis Associates AKNAD1 with Type-2 Diabetes in Jordan Subpopulations. Sci Rep 2015 Aug 21;5:13391
- de Miguel-Yanes JM, Shrader P, Pencina MJ, Fox CS, Manning AK, Grant RW, Dupuis J, Florez JC, D'Agostino RB Sr, Cupples LA, Meigs JB; MAGIC Investigators; DIAGRAM+ Investigators. Genetic risk reclassification for type 2 diabetes by age below or above 50 years using 40 type 2 diabetes risk single nucleotide polymorphisms. Diabetes Care 2011;34:121-125
- del Bosque-Plata L, Aguilar-Salinas CA, Tusié-Luna MT, Ramírez-Jiménez S, Rodríguez-Torres M, Aurón-Gómez M, Ramírez E, Velasco-Pérez ML, Ramírez-Silva A, Gómez-Pérez F, Hanis CL, Tsuchiya T, Yoshiuchi I, Cox NJ, Bell GI. Association of the calpain-10 gene with type 2 diabetes mellitus in a Mexican population. Mol Genet Metab 2004;81:122-126
- Diabetes Genetics Initiative of Broad Institute of Harvard and MIT, Lund University, and Novartis Institutes of BioMedical Research, Saxena R, Voight BF, Lyssenko V, Burtt NP, de Bakker PI, Chen H, Roix JJ, Kathiresan S, Hirschhorn JN, Daly MJ, Hughes TE, Groop L, Altshuler D, Almgren P, Florez JC, Meyer J, Ardlie K, Bengtsson Bostršm K, Isomaa B, Lettre G, Lindblad U, Lyon HN, Melander O, Newton-Cheh C, Nilsson P, Orho-Melander M, RŒstam L, Speliotes EK, Taskinen MR, Tuomi T, Guiducci C, Berglund A, Carlson J, Gianniny L, Hackett R, Hall L, Holmkvist J, Laurila E, Sjšgren M, Sterner M, Surti A, Svensson M, Svensson M, Tewhey R, Blumenstiel B, Parkin M, Defelice M, Barry R, Brodeur W, Camarata J, Chia N, Fava M, Gibbons J, Handsaker B, Healy C, Nguyen K, Gates C, Sougnez C, Gage D, Nizzari M, Gabriel SB, Chirn GW, Ma Q, Parikh H, Richardson D, Ricke D, Purcell S. Genome-wide association analysis identifies loci for type 2 diabetes and triglyceride levels. Science 2007;316:1331-1336
- DIAbetes Genetics Replication And Meta-analysis (DIAGRAM) Consortium, Asian Genetic Epidemiology Network Type 2 Diabetes (AGEN-T2D) Consortium, South Asian Type 2 Diabetes (SAT2D) Consortium, Mexican American Type 2 Diabetes (MAT2D) Consortium and Type 2 Diabetes Genetic Exploration by Next-generation sequencing in multi-Ethnic Samples (T2D-GENES) Consortium. Genome-wide trans-ancestry meta-analysis provides insight into the genetic architecture of type 2 diabetes susceptibility. Nature Genetics 2014, article accepted
- Dupuis J, Langenberg C, Prokopenko I, Saxena R, Soranzo N, Jackson AU, Wheeler E, Glazer NL, Bouatia-Naji N, Gloyn AL, Lindgren CM, Mägi R, Morris AP, Randall J, Johnson T, Elliott P, Rybin D, Thorleifsson G, Steinthorsdottir V, Henneman P, Grallert H, Dehghan A, Hottenga JJ, Franklin CS, Navarro P, Song K, Goel A, Perry JR, Egan JM, Lajunen T, Grarup N, Sparsø T, Doney A, Voight BF, Stringham HM, Li M, Kanoni S, Shrader P, Cavalcanti-Proença C, Kumari M, Qi L, Timpson NJ, Gieger C, Zabena C, Rocheleau G, Ingelsson E, An P, O'Connell J, Luan J, Elliott A, McCarroll SA, Payne F, Roccasecca RM, Pattou F, Sethupathy P, Ardlie K, Ariyurek Y, Balkau B, Barter P, Beilby JP, Ben-Shlomo Y, Benediktsson R, Bennett AJ, Bergmann S, Bochud M, Boerwinkle E, Bonnefond A, Bonnycastle LL, Borch-Johnsen K, Böttcher Y, Brunner E, Bumpstead SJ, Charpentier G, Chen YD, Chines P, Clarke R, Coin LJ, Cooper MN, Cornelis M, Crawford G, Crisponi L, Day IN, de Geus EJ, Delplanque J, Dina C, Erdos MR, Fedson AC, Fischer-Rosinsky A, Forouhi NG, Fox CS, Frants R, Franzosi MG, Galan P, Goodarzi MO, Graessler J, Groves CJ, Grundy S, Gwilliam R, Gyllensten U, Hadjadj S, Hallmans G, Hammond N, Han X, Hartikainen AL, Hassanali N, Hayward C, Heath SC, Hercberg S, Herder C, Hicks AA, Hillman DR, Hingorani AD, Hofman A, Hui J, Hung J, Isomaa B, Johnson PR, Jørgensen T, Jula A, Kaakinen M, Kaprio J, Kesaniemi YA, Kivimaki M, Knight B, Koskinen S, Kovacs P, Kyvik KO, Lathrop GM, Lawlor DA, Le Bacquer O, Lecoeur C, Li Y, Lyssenko V, Mahley R, Mangino M, Manning AK, Martínez-Larrad MT, McAteer JB, McCulloch LJ, McPherson R, Meisinger C, Melzer D, Meyre D, Mitchell BD, Morken MA, Mukherjee S, Naitza S, Narisu N, Neville MJ, Oostra BA, Orrù M, Pakyz R, Palmer CN, Paolisso G, Pattaro C, Pearson D, Peden JF, Pedersen NL, Perola M, Pfeiffer AF, Pichler I, Polasek O, Posthuma D, Potter SC, Pouta A, Province MA, Psaty BM, Rathmann W, Rayner NW, Rice K, Ripatti S, Rivadeneira F, Roden M, Rolandsson O, Sandbaek A, Sandhu M, Sanna S, Sayer AA, Scheet P, Scott LJ, Seedorf U, Sharp SJ, Shields B, Sigurethsson G, Sijbrands EJ, Silveira A, Simpson L, Singleton A, Smith NL, Sovio U, Swift A, Syddall H, Syvänen AC, Tanaka T, Thorand B, Tichet J, Tönjes A, Tuomi T, Uitterlinden AG, van Dijk KW, van Hoek M, Varma D, Visvikis-Siest S, Vitart V, Vogelzangs N, Waeber G, Wagner PJ, Walley A, Walters GB, Ward KL, Watkins H, Weedon MN, Wild SH, Willemsen G, Witteman JC, Yarnell JW, Zeggini E, Zelenika D, Zethelius B, Zhai G, Zhao JH, Zillikens MC; DIAGRAM Consortium; GIANT Consortium; Global BPgen Consortium, Borecki IB, Loos RJ, Meneton P, Magnusson PK, Nathan DM, Williams GH, Hattersley AT, Silander K, Salomaa V, Smith GD, Bornstein SR, Schwarz P, Spranger J, Karpe F, Shuldiner AR, Cooper C, Dedoussis GV, Serrano-Ríos M, Morris AD, Lind L, Palmer LJ, Hu FB, Franks PW, Ebrahim S, Marmot M, Kao WH, Pankow JS, Sampson MJ, Kuusisto J, Laakso M, Hansen T, Pedersen O, Pramstaller PP, Wichmann HE, Illig T, Rudan I, Wright AF, Stumvoll M, Campbell H, Wilson JF; Anders Hamsten on behalf of Procardis Consortium; MAGIC investigators, Bergman RN, Buchanan TA, Collins FS, Mohlke KL, Tuomilehto J, Valle TT, Altshuler D, Rotter JI, Siscovick DS, Penninx BW, Boomsma DI, Deloukas P, Spector TD, Frayling TM, Ferrucci L, Kong A, Thorsteinsdottir U, Stefansson K, van Duijn CM, Aulchenko YS, Cao A, Scuteri A, Schlessinger D, Uda M, Ruokonen A, Jarvelin MR, Waterworth DM, Vollenweider P, Peltonen L, Mooser V, Abecasis GR, Wareham NJ, Sladek R, Froguel P, Watanabe RM, Meigs JB, Groop L, Boehnke M, McCarthy MI, Florez JC, Barroso I. New genetic loci implicated in fasting glucose homeostasis and their impact on type 2 diabetes risk. Nat Genet 2010;42:105-116 [Erratum in Nat Genet 2010;42:464]
- Edghill EL, Flanagan SE, Patch AM, Boustred C, Parrish A, Shields B, Shepherd MH, Hussain K, Kapoor RR, Malecki M, MacDonald MJ, St¿y J, Steiner DF, Philipson LH, Bell GI; Neonatal Diabetes International Collaborative Group, Hattersley AT, Ellard S. Insulin mutation screening in 1,044 patients with diabetes: mutations in the INS gene are a common cause of neonatal diabetes but a rare cause of diabetes diagnosed in childhood or adulthood. Diabetes 2008;57:1034-1042
- Ellard S, Colclough K. Mutations in the genes encoding the transcription factors hepatocyte nuclear factor 1 alpha (HNF1A) and 4 alpha (HNF4A) in maturity-onset diabetes of the young. Hum Mutat 2006;27:854-869
- Franks PW, Rolandsson O, Debenham SL, et al. Replication of the association between variants in WFS1 and risk of type 2 diabetes in European populations. Diabetologia 2008;51:458-463 [Erratum, Diabetologia 2008;51:523]
- Frayling TM, Timpson NJ, Weedon MN, Zeggini E, Freathy RM, Lindgren CM, Perry JR, Elliott KS, Lango H, Rayner NW, Shields B, Harries LW, Barrett JC, Ellard S, Groves CJ, Knight B, Patch AM, Ness AR, Ebrahim S, Lawlor DA, Ring SM, Ben-Shlomo Y, Jarvelin MR, Sovio U, Bennett AJ, Melzer D, Ferrucci L, Loos RJ, Barroso I, Wareham NJ, Karpe F, Owen KR, Cardon LR, Walker M, Hitman GA, Palmer CN, Doney AS, Morris AD, Smith GD, Hattersley AT, McCarthy MI. A common variant in the FTO gene is associated with body mass index and predisposes to childhood and adult obesity. Science 2007;316:889-894
- George S, Rochford JJ, Wolfrum C, Gray SL, Schinner S, Wilson JC, Soos MA, Murgatroyd PR, Williams RM, Acerini CL, Dunger DB, Barford D, Umpleby AM, Wareham NJ, Davies HA, Schafer AJ, Stoffel M, O'Rahilly S, Barroso I. A family with severe insulin resistance and diabetes due to a mutation in AKT2. Science 2004;304:1325-1328
- Gloyn AL, Weedon MN, Owen KR, Turner MJ, Knight BA, Hitman G, Walker M, Levy JC, Sampson M, Halford S, McCarthy MI, Hattersley AT, Frayling TM. Large-scale association studies of variants in genes encoding the pancreatic beta-cell KATP channel subunits Kir6.2 (KCNJ11) and SUR1 (ABCC8) confirm that the KCNJ11 E23K variant is associated with type 2 diabetes. Diabetes 2003;52:568-572
- Grant SF, Thorleifsson G, Reynisdottir I, Benediktsson R, Manolescu A, Sainz J, Helgason A, Stefansson H, Emilsson V, Helgadottir A, Styrkarsdottir U, Magnusson KP, Walters GB, Palsdottir E, Jonsdottir T, Gudmundsdottir T, Gylfason A, Saemundsdottir J, Wilensky RL, Reilly MP, Rader DJ, Bagger Y, Christiansen C, Gudnason V, Sigurdsson G, Thorsteinsdottir U, Gulcher JR, Kong A, Stefansson K. Variant of transcription factor 7-like 2 (TCF7L2) gene confers risk of type 2 diabetes. Nat Genet 2006;38:320-323
- Grassi MA, Tikhomirov A, Ramalingam S, Below JE, Cox NJ, Nicolae DL. Genome-wide meta-analysis for severe diabetic retinopathy. Hum Mol Genet 2011;20:2472-2481
- Gudmundsson J, Sulem P, Steinthorsdottir V, Bergthorsson JT, Thorleifsson G, Manolescu A, Rafnar T, Gudbjartsson D, Agnarsson BA, Baker A, Sigurdsson A, Benediktsdottir KR, Jakobsdottir M, Blondal T, Stacey SN, Helgason A, Gunnarsdottir S, Olafsdottir A, Kristinsson KT, Birgisdottir B, Ghosh S, Thorlacius S, Magnusdottir D, Stefansdottir G, Kristjansson K, Bagger Y, Wilensky RL, Reilly MP, Morris AD, Kimber CH, Adeyemo A, Chen Y, Zhou J, So WY, Tong PC, Ng MC, Hansen T, Andersen G, Borch-Johnsen K, Jorgensen T, Tres A, Fuertes F, Ruiz-Echarri M, Asin L, Saez B, van Boven E, Klaver S, Swinkels DW, Aben KK, Graif T, Cashy J, Suarez BK, van Vierssen Trip O, Frigge ML, Ober C, Hofker MH, Wijmenga C, Christiansen C, Rader DJ, Palmer CN, Rotimi C, Chan JC, Pedersen O, Sigurdsson G, Benediktsson R, Jonsson E, Einarsson GV, Mayordomo JI, Catalona WJ, Kiemeney LA, Barkardottir RB, Gulcher JR, Thorsteinsdottir U, Kong A, Stefansson K. Two variants on chromosome 17 confer prostate cancer risk, and the one in TCF2 protects against type 2 diabetes. Nat Genet 2007;39:977-983
- Guzmán-Flores JM, Muñoz-Valle JF, Sánchez-Corona J, Cobián JG, Medina-Carrillo L, García-Zapién AG, Cruz-Quevedo EG, Flores-Martínez SE. Tumor necrosis factor-alpha gene promoter -308G/A and -238G/A polymorphisms in Mexican patients with type 2 diabetes mellitus. Dis Markers 2011;30:19-24
- Hager J, Hansen L, Vaisse C, Vionnet N, Philippi A, Poller W, Velho G, Carcassi C, Contu L, Julier C, Cambien F, Passa P, Lathrop M, Kindsvogel W, Demenais F, Nishimura E, Froguel P. A missense mutation in the glucagon receptor gene is associated with non-insulin-dependent diabetes mellitus. Nat Genet 1995;9:299-304
- Hansen L, Hansen T, Vestergaard H, Bjørbaek C, Echwald SM, Clausen JO, Chen YH, Chen MX, Cohen PT, Pedersen O. A widespread amino acid polymorphism at codon 905 of the glycogen-associated regulatory subunit of protein phosphatase-1 is associated with insulin resistance and hypersecretion of insulin. Hum Mol Genet 1995;4:1313-1320
- Harder MN, Ribel-Madsen R, Justesen JM, Sparsø T, Andersson EA, Grarup N, Jørgensen T, Linneberg A, Hansen T, Pedersen O. Type 2 diabetes risk alleles near BCAR1 and in ANK1 associate with decreased β-cell function whereas risk alleles near ANKRD55 and GRB14 associate with decreased insulin sensitivity in the Danish Inter99 cohort. J Clin Endocrinol Metab 2013;98:E801-E806
- Hegele RA, Cao H, Liu DM, Costain GA, Charlton-Menys V, Rodger NW, Durrington PN. Sequencing of the reannotated LMNB2 gene reveals novel mutations in patients with acquired partial lipodystrophy. Am J Hum Genet 2006;79:383-389
- Hu C, Zhang R, Wang C, Wang J, Ma X, Hou X, Lu J, Yu W, Jiang F, Bao Y, Xiang K, Jia W. Variants from GIPR, TCF7L2, DGKB, MADD, CRY2, GLIS3, PROX1, SLC30A8 and IGF1 are associated with glucose metabolism in the Chinese.PLoS One 2010;5:e15542
- Imamura M, Iwata M, Maegawa H, Watada H, Hirose H, Tanaka Y, Tobe K, Kaku K, Kashiwagi A, Kawamori R, Nakamura Y, Maeda S. Genetic variants at CDC123/CAMK1D and SPRY2 are associated with susceptibility to type 2 diabetes in the Japanese population. Diabetologia 2011;54:3071-3077
- Imamura M, Maeda S, Yamauchi T, Hara K, Yasuda K, Morizono T, Takahashi A, Horikoshi M, Nakamura M, Fujita H, Tsunoda T, Kubo M, Watada H, Maegawa H, Okada-Iwabu M, Iwabu M, Shojima N, Ohshige T, Omori S, Iwata M, Hirose H, Kaku K, Ito C, Tanaka Y, Tobe K, Kashiwagi A, Kawamori R, Kasuga M, Kamatani N; Diabetes Genetics Replication and Meta-analysis (DIAGRAM) Consortium, Nakamura Y, Kadowaki T. A single-nucleotide polymorphism in ANK1 is associated with susceptibility to type 2 diabetes in Japanese populations. Hum Mol Genet 2012;21:3042-3049
- Jeon JP, Shim SM, Nam HY, Ryu GM, Hong EJ, Kim HL, Han BG. Copy number variation at leptin receptor gene locus associated with metabolic traits and the risk of type 2 diabetes mellitus. BMC Genomics 2010;11:426
- Jin J, Cao L, Zhao Z, Shen S, Kiess W, Zhi D, Ye R, Cheng R, Chen L, Yang Y, Luo F. Novel BSCL2 gene mutation E189X in Chinese congenital generalized lipodystrophy child with early onset diabetes mellitus. Eur J Endocrinol 2007;157:783-787
- Kamiya M, Judson H, Okazaki Y, Kusakabe M, Muramatsu M, Takada S, Takagi N, Arima T, Wake N, Kamimura K, Satomura K, Hermann R, Bonthron DT, Hayashizaki Y. The cell cycle control gene ZAC/PLAGL1 is imprinted--a strong candidate gene for transient neonatal diabetes. Hum Mol Genet 2000;9:453-460
- Kim CA, DelŽpine M, Boutet E, El Mourabit H, Le Lay S, Meier M, Nemani M, Bridel E, Leite CC, Bertola DR, Semple RK, O'Rahilly S, Dugail I, Capeau J, Lathrop M, MagrŽ J. Association of a homozygous nonsense caveolin-1 mutation with Berardinelli-Seip congenital lipodystrophy. J Clin Endocrinol Metab 2008;93:1129-1134
- Kodama S, Yamada T, Imai J, Sawada S, Takahashi K, Tsukita S, Kaneko K, Uno K, Ishigaki Y, Oka Y, Katagiri H. Simultaneous copy number losses within multiple subtelomeric regions in early-onset type 2 diabetes mellitus. PLoS One 2014 Apr 7;9(4):e88602
- Kong A, Steinthorsdottir V, Masson G, Thorleifsson G, Sulem P, Besenbacher S, Jonasdottir A, Sigurdsson A, Kristinsson KT, Jonasdottir A, Frigge ML, Gylfason A, Olason PI, Gudjonsson SA, Sverrisson S, Stacey SN, Sigurgeirsson B, Benediktsdottir KR, Sigurdsson H, Jonsson T, Benediktsson R, Olafsson JH, Johannsson OT, Hreidarsson AB, Sigurdsson G; DIAGRAM Consortium, Ferguson-Smith AC, Gudbjartsson DF, Thorsteinsdottir U, Stefansson K. Parental origin of sequence variants associated with complex diseases. Nature 2009;462:868-874
- Kooner JS, Saleheen D, Sim X, Sehmi J, Zhang W, Frossard P, Been LF, Chia KS, Dimas AS, Hassanali N, Jafar T, Jowett JB, Li X, Radha V, Rees SD, Takeuchi F, Young R, Aung T, Basit A, Chidambaram M, Das D, Grundberg E, Hedman AK, Hydrie ZI, Islam M, Khor CC, Kowlessur S, Kristensen MM, Liju S, Lim WY, Matthews DR, Liu J, Morris AP, Nica AC, Pinidiyapathirage JM, Prokopenko I, Rasheed A, Samuel M, Shah N, Shera AS, Small KS, Suo C, Wickremasinghe AR, Wong TY, Yang M, Zhang F; DIAGRAM; MuTHER, Abecasis GR, Barnett AH, Caulfield M, Deloukas P, Frayling TM, Froguel P, Kato N, Katulanda P, Kelly MA, Liang J, Mohan V, Sanghera DK, Scott J, Seielstad M, Zimmet PZ, Elliott P, Teo YY, McCarthy MI, Danesh J, Tai ES, Chambers JC. Genome-wide association study in individuals of South Asian ancestry identifies six new type 2 diabetes susceptibility loci. Nat Genet 2011;43:984-989
- Kristinsson SY, Thorolfsdottir ET, Talseth B, Steingrimsson E, Thorsson AV, Helgason T, Hreidarsson AB, Arngrimsson R. MODY in Iceland is associated with mutations in HNF-1alpha and a novel mutation in NeuroD1. Diabetologia 2001;44:2098-2103
- Kubaszek A, Markkanen A, Eriksson JG, Forsen T, Osmond C, Barker DJ, Laakso M. The association of the K121Q polymorphism of the plasma cell glycoprotein-1 gene with type 2 diabetes and hypertension depends on size at birth. J Clin Endocrinol Metab 2004;89:2044-2047
- Kudo H, Emi M, Ishigaki Y, Tsunoda U, Hinokio Y, Ishii M, Sato H, Yamada T,Katagiri H, Oka Y. Frequent loss of genome gap region in 4p16.3 subtelomere in early-onset type 2 diabetes mellitus. Exp Diabetes Res 2011;2011:498460
- Kusari J, Verma US, Buse JB, Henry RR, Olefsky JM. Analysis of the gene sequences of the insulin receptor and the insulin-sensitive glucose transporter (GLUT-4) in patients with common-type non-insulin-dependent diabetes mellitus. J Clin Invest 1991;88:1323-1330
- Lee HS, Moon S, Yun JH, Lee M, Hwang MY, Kim YJ, Han BG, Kim JM, Kim BJ. Genome-wide copy number variation study reveals KCNIP1 as a modulator of insulin secretion. Genomics 2014 Aug;104(2):113-120
- Li H, Gan W, Lu L, Dong X, Han X, Hu C, Yang Z, Sun L, Bao W, Li P, He M, Sun L, Wang Y, Zhu J, Ning Q, Tang Y, Zhang R, Wen J, Wang D, Zhu X, Guo K, Zuo X, Guo X, Yang H, Zhou X; DIAGRAM Consortium; AGEN-T2D Consortium, Zhang X, Qi L, Loos RJ, Hu FB, Wu T, Liu Y, Liu L, Yang Z, Hu R, Jia W, Ji L, Li Y, Lin X. A genome-wide association study identifies GRK5 and RASGRP1 as type 2 diabetes loci in Chinese Hans. Diabetes 2013;62:291-298
- Lyssenko V, Nagorny CL, Erdos MR, Wierup N, Jonsson A, Spégel P, Bugliani M, Saxena R, Fex M, Pulizzi N, Isomaa B, Tuomi T, Nilsson P, Kuusisto J, Tuomilehto J, Boehnke M, Altshuler D, Sundler F, Eriksson JG, Jackson AU, Laakso M, Marchetti P, Watanabe RM, Mulder H, Groop L. Common variant in MTNR1B associated with increased risk of type 2 diabetes and impaired early insulin secretion. Nat Genet 2009;41:82-88
- Mammarella S, Romano F, Di Valerio A, Creati B, Esposito DL, Palmirotta R, Capani F, Vitullo P, Volpe G, Battista P, Della Loggia F, Mariani-Costantini R, Cama A. Interaction between the G1057D variant of IRS-2 and overweight in the pathogenesis of type 2 diabetes. Hum Mol Genet 2000;9:2517-2521
- Mammarella S, Romano F, Di Valerio A, Creati B, Esposito DL, Palmirotta R, Capani F, Vitullo P, Volpe G, Battista P, Della Loggia F, Mariani-Costantini R, Cama A. Interaction between the G1057D variant of IRS-2 and overweight in the pathogenesis of type 2 diabetes. Hum Mol Genet 2000;9:2517-2521
- Mok A, Cao H, Zinman B, Hanley AJ, Harris SB, Kennedy BP, Hegele RA. A single nucleotide polymorphism in protein tyrosine phosphatase PTP-1B is associated with protection from diabetes or impaired glucose tolerance in Oji-Cree. J Clin Endocrinol Metab 2002;87:724-727
- Molven A, Ringdal M, Nordb¿ AM, Raeder H, St¿y J, Lipkind GM, Steiner DF, Philipson LH, Bergmann I, Aarskog D, Undlien DE, Joner G, S¿vik O; Norwegian Childhood Diabetes Study Group, Bell GI, Nj¿lstad PR. Mutations in the insulin gene can cause MODY and autoantibody-negative type 1 diabetes. Diabetes 2008;57:1131-1135
- Morris AP, Voight BF, Teslovich TM, Ferreira T, Segrè AV, Steinthorsdottir V, Strawbridge RJ, Khan H, Grallert H, Mahajan A, Prokopenko I, Kang HM, Dina C, Esko T, Fraser RM, Kanoni S, Kumar A, Lagou V, Langenberg C, Luan J, Lindgren CM, Müller-Nurasyid M, Pechlivanis S, Rayner NW, Scott LJ, Wiltshire S, Yengo L, Kinnunen L, Rossin EJ, Raychaudhuri S, Johnson AD, Dimas AS, Loos RJ, Vedantam S, Chen H, Florez JC, Fox C, Liu CT, Rybin D, Couper DJ, Kao WH, Li M, Cornelis MC, Kraft P, Sun Q, van Dam RM, Stringham HM, Chines PS, Fischer K, Fontanillas P, Holmen OL, Hunt SE, Jackson AU, Kong A, Lawrence R, Meyer J, Perry JR, Platou CG, Potter S, Rehnberg E, Robertson N, Sivapalaratnam S, Stančáková A, Stirrups K, Thorleifsson G, Tikkanen E, Wood AR, Almgren P, Atalay M, Benediktsson R, Bonnycastle LL, Burtt N, Carey J, Charpentier G, Crenshaw AT, Doney AS, Dorkhan M, Edkins S, Emilsson V, Eury E, Forsen T, Gertow K, Gigante B, Grant GB, Groves CJ, Guiducci C, Herder C, Hreidarsson AB, Hui J, James A, Jonsson A, Rathmann W, Klopp N, Kravic J, Krjutškov K, Langford C, Leander K, Lindholm E, Lobbens S, Männistö S, Mirza G, Mühleisen TW, Musk B, Parkin M, Rallidis L, Saramies J, Sennblad B, Shah S, Sigurðsson G, Silveira A, Steinbach G, Thorand B, Trakalo J, Veglia F, Wennauer R, Winckler W, Zabaneh D, Campbell H, van Duijn C, Uitterlinden AG, Hofman A, Sijbrands E, Abecasis GR, Owen KR, Zeggini E, Trip MD, Forouhi NG, Syvänen AC, Eriksson JG, Peltonen L, Nöthen MM, Balkau B, Palmer CN, Lyssenko V, Tuomi T, Isomaa B, Hunter DJ, Qi L; Wellcome Trust Case Control Consortium; Meta-Analyses of Glucose and Insulin-related traits Consortium (MAGIC) Investigators; Genetic Investigation of ANthropometric Traits (GIANT) Consortium; Asian Genetic Epidemiology Network–Type 2 Diabetes (AGEN-T2D) Consortium; South Asian Type 2 Diabetes (SAT2D) Consortium, Shuldiner AR, Roden M, Barroso I, Wilsgaard T, Beilby J, Hovingh K, Price JF, Wilson JF, Rauramaa R, Lakka TA, Lind L, Dedoussis G, Njølstad I, Pedersen NL, Khaw KT, Wareham NJ, Keinanen-Kiukaanniemi SM, Saaristo TE, Korpi-Hyövälti E, Saltevo J, Laakso M, Kuusisto J, Metspalu A, Collins FS, Mohlke KL, Bergman RN, Tuomilehto J, Boehm BO, Gieger C, Hveem K, Cauchi S, Froguel P, Baldassarre D, Tremoli E, Humphries SE, Saleheen D, Danesh J, Ingelsson E, Ripatti S, Salomaa V, Erbel R, Jöckel KH, Moebus S, Peters A, Illig T, de Faire U, Hamsten A, Morris AD, Donnelly PJ, Frayling TM, Hattersley AT, Boerwinkle E, Melander O, Kathiresan S, Nilsson PM, Deloukas P, Thorsteinsdottir U, Groop LC, Stefansson K, Hu F, Pankow JS, Dupuis J, Meigs JB, Altshuler D, Boehnke M, McCarthy MI; DIAbetes Genetics Replication And Meta-analysis (DIAGRAM). Large-scale association analysis provides insights into the genetic architecture and pathophysiology of type 2 diabetes. Nat Genet 2012;44:981-990
- Mueckler M, Kruse M, Strube M, Riggs AC, Chiu KC, Permutt MA. A mutation in the Glut2 glucose transporter gene of a diabetic patient abolishes transport activity. J Biol Chem 1994;269:17765-17767
- Nicolino M, Claiborn KC, SenŽe V, Boland A, Stoffers DA, Julier C. A novel hypomorphic PDX1 mutation responsible for permanent neonatal diabetes with subclinical exocrine deficiency. Diabetes 2010;59:733-740
- Novials A, Vidal J, Franco C, Ribera F, Sener A, Malaisse WJ, Gomis R. Mutation in the calcium-binding domain of the mitochondrial glycerophosphate dehydrogenase gene in a family of diabetic subjects. Biochem Biophys Res Commun 1997;231:570-572
- Palmer ND, McDonough CW, Hicks PJ, Roh BH, Wing MR, An SS, Hester JM, Cooke JN, Bostrom MA, Rudock ME, Talbert ME, Lewis JP; DIAGRAM Consortium; MAGIC Investigators, Ferrara A, Lu L, Ziegler JT, Sale MM, Divers J, Shriner D, Adeyemo A, Rotimi CN, Ng MC, Langefeld CD, Freedman BI, Bowden DW, Voight BF, Scott LJ, Steinthorsdottir V, Morris AP, Dina C, Welch RP, Zeggini E, Huth C, Aulchenko YS, Thorleifsson G, McCulloch LJ, Ferreira T, Grallert H, Amin N, Wu G, Willer CJ, Raychaudhuri S, McCarroll SA, Langenberg C, Hofmann OM, Dupuis J, Qi L, Segrè AV, van Hoek M, Navarro P, Ardlie K, Balkau B, Benediktsson R, Bennett AJ, Blagieva R, Boerwinkle E, Bonnycastle LL, Boström KB, Bravenboer B, Bumpstead S, Burtt NP, Charpentier G, Chines PS, Cornelis M, Couper DJ, Crawford G, Doney AS, Elliott KS, Elliott AL, Erdos MR, Fox CS, Franklin CS, Ganser M, Gieger C, Grarup N, Green T, Griffin S, Groves CJ, Guiducci C, Hadjadj S, Hassanali N, Herder C, Isomaa B, Jackson AU, Johnson PR, Jørgensen T, Kao WH, Klopp N, Kong A, Kraft P, Kuusisto J, Lauritzen T, Li M, Lieverse A, Lindgren CM, Lyssenko V, Marre M, Meitinger T, Midthjell K, Morken MA, Narisu N, Nilsson P, Owen KR, Payne F, Perry JR, Petersen AK, Platou C, Proença C, Prokopenko I, Rathmann W, Rayner NW, Robertson NR, Rocheleau G, Roden M, Sampson MJ, Saxena R, Shields BM, Shrader P, Sigurdsson G, Sparsø T, Strassburger K, Stringham HM, Sun Q, Swift AJ, Thorand B, Tichet J, Tuomi T, van Dam RM, van Haeften TW, van Herpt T, van Vliet-Ostaptchouk JV, Walters GB, Weedon MN, Wijmenga C, Witteman J, Bergman RN, Cauchi S, Collins FS, Gloyn AL, Gyllensten U, Hansen T, Hide WA, Hitman GA, Hofman A, Hunter DJ, Hveem K, Laakso M, Mohlke KL, Morris AD, Palmer CN, Pramstaller PP, Rudan I, Sijbrands E, Stein LD, Tuomilehto J, Uitterlinden A, Walker M, Wareham NJ, Watanabe RM, Abecasis GR, Boehm BO, Campbell H, Daly MJ, Hattersley AT, Hu FB, Meigs JB, Pankow JS, Pedersen O, Wichmann HE, Barroso I, Florez JC, Frayling TM, Groop L, Sladek R, Thorsteinsdottir U, Wilson JF, Illig T, Froguel P, van Duijn CM, Stefansson K, Altshuler D, Boehnke M, McCarthy MI, Soranzo N, Wheeler E, Glazer NL, Bouatia-Naji N, Mägi R, Randall J, Johnson T, Elliott P, Rybin D, Henneman P, Dehghan A, Hottenga JJ, Song K, Goel A, Egan JM, Lajunen T, Doney A, Kanoni S, Cavalcanti-Proença C, Kumari M, Timpson NJ, Zabena C, Ingelsson E, An P, O'Connell J, Luan J, Elliott A, McCarroll SA, Roccasecca RM, Pattou F, Sethupathy P, Ariyurek Y, Barter P, Beilby JP, Ben-Shlomo Y, Bergmann S, Bochud M, Bonnefond A, Borch-Johnsen K, Böttcher Y, Brunner E, Bumpstead SJ, Chen YD, Chines P, Clarke R, Coin LJ, Cooper MN, Crisponi L, Day IN, de Geus EJ, Delplanque J, Fedson AC, Fischer-Rosinsky A, Forouhi NG, Frants R, Franzosi MG, Galan P, Goodarzi MO, Graessler J, Grundy S, Gwilliam R, Hallmans G, Hammond N, Han X, Hartikainen AL, Hayward C, Heath SC, Hercberg S, Hicks AA, Hillman DR, Hingorani AD, Hui J, Hung J, Jula A, Kaakinen M, Kaprio J, Kesaniemi YA, Kivimaki M, Knight B, Koskinen S, Kovacs P, Kyvik KO, Lathrop GM, Lawlor DA, Le Bacquer O, Lecoeur C, Li Y, Mahley R, Mangino M, Manning AK, Martínez-Larrad MT, McAteer JB, McPherson R, Meisinger C, Melzer D, Meyre D, Mitchell BD, Mukherjee S, Naitza S, Neville MJ, Oostra BA, Orrù M, Pakyz R, Paolisso G, Pattaro C, Pearson D, Peden JF, Pedersen NL, Perola M, Pfeiffer AF, Pichler I, Polasek O, Posthuma D, Potter SC, Pouta A, Province MA, Psaty BM, Rayner NW, Rice K, Ripatti S, Rivadeneira F, Rolandsson O, Sandbaek A, Sandhu M, Sanna S, Sayer AA, Scheet P, Seedorf U, Sharp SJ, Shields B, Sijbrands EJ, Silveira A, Simpson L, Singleton A, Smith NL, Sovio U, Swift A, Syddall H, Syvänen AC, Tanaka T, Tönjes A, Uitterlinden AG, van Dijk KW, Varma D, Visvikis-Siest S, Vitart V, Vogelzangs N, Waeber G, Wagner PJ, Walley A, Ward KL, Watkins H, Wild SH, Willemsen G, Witteman JC, Yarnell JW, Zelenika D, Zethelius B, Zhai G, Zhao JH, Zillikens MC, Borecki IB, Loos RJ, Meneton P, Magnusson PK, Nathan DM, Williams GH, Silander K, Salomaa V, Smith GD, Bornstein SR, Schwarz P, Spranger J, Karpe F, Shuldiner AR, Cooper C, Dedoussis GV, Serrano-Ríos M, Lind L, Palmer LJ, Franks PW, Ebrahim S, Marmot M, Kao WH, Pramstaller PP, Wright AF, Stumvoll M, Hamsten A, Buchanan TA, Valle TT, Rotter JI, Siscovick DS, Penninx BW, Boomsma DI, Deloukas P, Spector TD, Ferrucci L, Cao A, Scuteri A, Schlessinger D, Uda M, Ruokonen A, Jarvelin MR, Waterworth DM, Vollenweider P, Peltonen L, Mooser V, Sladek R.A genome-wide association search for type 2 diabetes genes in African Americans. PLoS One 2012;7:e29202
- Pearl EJ, Jarikji Z, Horb ME. Functional analysis of Rfx6 and mutant variants associated with neonatal diabetes. Dev Biol 2011;351:135-1345
- Perez-Luque E, Malacara JM, Garay-Sevilla ME, Fajardo ME. Association of the TNF-α -308G/A polymorphism with family history of type 2 diabetes mellitus in a Mexican population. Clin Biochem 2012;45:12-15
- Perry JR, McCarthy MI, Hattersley AT, Zeggini E; Wellcome Trust Case Control Consortium, Weedon MN, Frayling TM. Interrogating type 2 diabetes genome-wide association data using a biological pathway-based approach. Diabetes 2009;58:1463-1467
- Plengvidhya N, Kooptiwut S, Songtawee N, Doi A, Furuta H, Nishi M, Nanjo K, Tantibhedhyangkul W, Boonyasrisawat W, Yenchitsomanus PT, Doria A, Banchuin N. PAX4 mutations in Thais with maturity onset diabetes of the young. J Clin Endocrinol Metab 2007;92:2821-2826
- Prokopenko I, Langenberg C, Florez JC, Saxena R, Soranzo N, Thorleifsson G, Loos RJ, Manning AK, Jackson AU, Aulchenko Y, Potter SC, Erdos MR, Sanna S, Hottenga JJ, Wheeler E, Kaakinen M, Lyssenko V, Chen WM, Ahmadi K, Beckmann JS, Bergman RN, Bochud M, Bonnycastle LL, Buchanan TA, Cao A, Cervino A, Coin L, Collins FS, Crisponi L, de Geus EJ, Dehghan A, Deloukas P, Doney AS, Elliott P, Freimer N, Gateva V, Herder C, Hofman A, Hughes TE, Hunt S, Illig T, Inouye M, Isomaa B, Johnson T, Kong A, Krestyaninova M, Kuusisto J, Laakso M, Lim N, Lindblad U, Lindgren CM, McCann OT, Mohlke KL, Morris AD, Naitza S, Orrù M, Palmer CN, Pouta A, Randall J, Rathmann W, Saramies J, Scheet P, Scott LJ, Scuteri A, Sharp S, Sijbrands E, Smit JH, Song K, Steinthorsdottir V, Stringham HM, Tuomi T, Tuomilehto J, Uitterlinden AG, Voight BF, Waterworth D, Wichmann HE, Willemsen G, Witteman JC, Yuan X, Zhao JH, Zeggini E, Schlessinger D, Sandhu M, Boomsma DI, Uda M, Spector TD, Penninx BW, Altshuler D, Vollenweider P, Jarvelin MR, Lakatta E, Waeber G, Fox CS, Peltonen L, Groop LC, Mooser V, Cupples LA, Thorsteinsdottir U, Boehnke M, Barroso I, Van Duijn C, Dupuis J, Watanabe RM, Stefansson K, McCarthy MI, Wareham NJ, Meigs JB, Abecasis GR. Variants in MTNR1B influence fasting glucose levels. Nat Genet 2009;41:77-81
- Qi L, Cornelis MC, Kraft P, Stanya KJ, Linda Kao WH, Pankow JS, Dupuis J, Florez JC, Fox CS, Paré G, Sun Q, Girman CJ, Laurie CC, Mirel DB, Manolio TA, Chasman DI, Boerwinkle E, Ridker PM, Hunter DJ, Meigs JB, Lee CH, Hu FB, van Dam RM; Meta-Analysis of Glucose and Insulin-related traits Consortium (MAGIC); Diabetes Genetics Replication and Meta-analysis (DIAGRAM) Consortium. Genetic variants at 2q24 are associated with susceptibility to type 2 diabetes. Hum Mol Genet 2010;19:2706-2715
- Qi L, Meigs J, Manson JE, Ma J, Hunter D, Rifai N, Hu FB. HFE genetic variability, body iron stores, and the risk of type 2 diabetes in U.S. women. Diabetes 2005;54:3567-3572
- Richards JB, Waterworth D, O'Rahilly S, Hivert MF, Loos RJ, Perry JR, Tanaka T, Timpson NJ, Semple RK, Soranzo N, Song K, Rocha N, Grundberg E, Dupuis J, Florez JC, Langenberg C, Prokopenko I, Saxena R, Sladek R, Aulchenko Y, Evans D, Waeber G, Erdmann J, Burnett MS, Sattar N, Devaney J, Willenborg C, Hingorani A, Witteman JC, Vollenweider P, Glaser B, Hengstenberg C, Ferrucci L, Melzer D, Stark K, Deanfield J, Winogradow J, Grassl M, Hall AS, Egan JM, Thompson JR, Ricketts SL, König IR, Reinhard W, Grundy S, Wichmann HE, Barter P, Mahley R, Kesaniemi YA, Rader DJ, Reilly MP, Epstein SE, Stewart AF, Van Duijn CM, Schunkert H, Burling K, Deloukas P, Pastinen T, Samani NJ, McPherson R, Davey Smith G, Frayling TM, Wareham NJ, Meigs JB, Mooser V, Spector TD; GIANT Consortium. A genome-wide association study reveals variants in ARL15 that influence adiponectin levels. PLoS Genet 2009;5:e1000768
- Rung J, Cauchi S, Albrechtsen A, Shen L, Rocheleau G, Cavalcanti-Proença C, Bacot F, Balkau B, Belisle A, Borch-Johnsen K, Charpentier G, Dina C, Durand E, Elliott P, Hadjadj S, Järvelin MR, Laitinen J, Lauritzen T, Marre M, Mazur A, Meyre D, Montpetit A, Pisinger C, Posner B, Poulsen P, Pouta A, Prentki M, Ribel-Madsen R, Ruokonen A, Sandbaek A, Serre D, Tichet J, Vaxillaire M, Wojtaszewski JF, Vaag A, Hansen T, Polychronakos C, Pedersen O, Froguel P, Sladek R. Genetic variant near IRS1 is associated with type 2 diabetes, insulin resistance and hyperinsulinemia. Nat Genet 2009;41:1110-1115
- Sandhu MS, Weedon MN, Fawcett KA, Wasson J, Debenham SL, Daly A, Lango H, Frayling TM, Neumann RJ, Sherva R, Blech I, Pharoah PD, Palmer CN, Kimber C, Tavendale R, Morris AD, McCarthy MI, Walker M, Hitman G, Glaser B, Permutt MA, Hattersley AT, Wareham NJ, Barroso I. Common variants in WFS1 confer risk of type 2 diabetes. Nat Genet 2007;39:951-953.
- Scott LJ, Mohlke KL, Bonnycastle LL, Willer CJ, Li Y, Duren WL, Erdos MR, Stringham HM, Chines PS, Jackson AU, Prokunina-Olsson L, Ding CJ, Swift AJ, Narisu N, Hu T, Pruim R, Xiao R, Li XY, Conneely KN, Riebow NL, Sprau AG, Tong M, White PP, Hetrick KN, Barnhart MW, Bark CW, Goldstein JL, Watkins L, Xiang F, Saramies J, Buchanan TA, Watanabe RM, Valle TT, Kinnunen L, Abecasis GR, Pugh EW, Doheny KF, Bergman RN, Tuomilehto J, Collins FS, Boehnke M. A genome-wide association study of type 2 diabetes in Finns detects multiple susceptibility variants. Science 2007;316:1341-1345
- Shimajiri Y, Sanke T, Furuta H, Hanabusa T, Nakagawa T, Fujitani Y, Kajimoto Y, Takasu N, Nanjo K. A missense mutation of Pax4 gene (R121W) is associated with type 2 diabetes in Japanese. Diabetes 2001;50:2864-2869
- Sladek R, Rocheleau G, Rung J, Dina C, Shen L, Serre D, Boutin P, Vincent D, Belisle A, Hadjadj S, Balkau B, Heude B, Charpentier G, Hudson TJ, Montpetit A, Pshezhetsky AV, Prentki M, Posner BI, Balding DJ, Meyre D, Polychronakos C, Froguel P. A genome-wide association study identifies novel risk loci for type 2 diabetes. Nature 2007;445:881-885
- Steinthorsdottir V, Thorleifsson G, Reynisdottir I, Benediktsson R, Jonsdottir T, Walters GB, Styrkarsdottir U, Gretarsdottir S, Emilsson V, Ghosh S, Baker A, Snorradottir S, Bjarnason H, Ng MC, Hansen T, Bagger Y, Wilensky RL, Reilly MP, Adeyemo A, Chen Y, Zhou J, Gudnason V, Chen G, Huang H, Lashley K, Doumatey A, So WY, Ma RC, Andersen G, Borch-Johnsen K, Jorgensen T, van Vliet-Ostaptchouk JV, Hofker MH, Wijmenga C, Christiansen C, Rader DJ, Rotimi C, Gurney M, Chan JC, Pedersen O, Sigurdsson G, Gulcher JR, Thorsteinsdottir U, Kong A, Stefansson K. A variant in CDKAL1 influences insulin response and risk of type 2 diabetes. Nat Genet 2007;39:770-775
- Strawbridge RJ, Dupuis J, Prokopenko I, Barker A, Ahlqvist E, Rybin D, Petrie JR, Travers ME, Bouatia-Naji N, Dimas AS, Nica A, Wheeler E, Chen H, Voight BF, Taneera J, Kanoni S, Peden JF, Turrini F, Gustafsson S, Zabena C, Almgren P, Barker DJ, Barnes D, Dennison EM, Eriksson JG, Eriksson P, Eury E, Folkersen L, Fox CS, Frayling TM, Goel A, Gu HF, Horikoshi M, Isomaa B, Jackson AU, Jameson KA, Kajantie E, Kerr-Conte J, Kuulasmaa T, Kuusisto J, Loos RJ, Luan J, Makrilakis K, Manning AK, Martínez-Larrad MT, Narisu N, Nastase Mannila M, Ohrvik J, Osmond C, Pascoe L, Payne F, Sayer AA, Sennblad B, Silveira A, Stancáková A, Stirrups K, Swift AJ, Syvänen AC, Tuomi T, van 't Hooft FM, Walker M, Weedon MN, Xie W, Zethelius B; DIAGRAM Consortium; GIANT Consortium; MuTHER Consortium; CARDIoGRAM Consortium; C4D Consortium, Ongen H, Mälarstig A, Hopewell JC, Saleheen D, Chambers J, Parish S, Danesh J, Kooner J, Ostenson CG, Lind L, Cooper CC, Serrano-Ríos M, Ferrannini E, Forsen TJ, Clarke R, Franzosi MG, Seedorf U, Watkins H, Froguel P, Johnson P, Deloukas P, Collins FS, Laakso M, Dermitzakis ET, Boehnke M, McCarthy MI, Wareham NJ, Groop L, Pattou F, Gloyn AL, Dedoussis GV, Lyssenko V, Meigs JB, Barroso I, Watanabe RM, Ingelsson E, Langenberg C, Hamsten A, Florez JC. Genome-wide association identifies nine common variants associated with fasting proinsulin levels and provides new insights into the pathophysiology of type 2 diabetes. Diabetes 2011;60:2624-2634
- Tabassum R, Chauhan G, Dwivedi OP, Mahajan A, Jaiswal A, Kaur I, Bandesh K, Singh T, Mathai BJ, Pandey Y, Chidambaram M, Sharma A, Chavali S, Sengupta S, Ramakrishnan L, Venkatesh P, Aggarwal SK, Ghosh S, Prabhakaran D, Srinath RK, Saxena M, Banerjee M, Mathur S, Bhansali A, Shah VN, Madhu SV, Marwaha RK, Basu A, Scaria V, McCarthy MI; DIAGRAM; INDICO, Venkatesan R, Mohan V, Tandon N, Bharadwaj D. Genome-wide association study for type 2 diabetes in Indians identifies a new susceptibility locus at 2q21. Diabetes 2013;62:977-986
- Tan MS, Chang SY, Chang DM, Tsai JC, Lee YJ. Association of resistin gene 3'-untranslated region +62G-->A polymorphism with type 2 diabetes and hypertension in a Chinese population. J Clin Endocrinol Metab 2003;88:1258-1263
- Todorova B, Kubaszek A, Pihlajamäki J, Lindström J, Eriksson J, Valle TT, Hämäläinen H, Ilanne-Parikka P, Keinänen-Kiukaanniemi S, Tuomilehto J, Uusitupa M, Laakso M; Finnish Diabetes Prevention Study. The G-250A promoter polymorphism of the hepatic lipase gene predicts the conversion from impaired glucose tolerance to type 2 diabetes mellitus: the Finnish Diabetes Prevention Study. J Clin Endocrinol Metab 2004;89:2019-2023
- Torsvik J, Johansson S, Johansen A, Ek J, Minton J, Raeder H, Ellard S, Hattersley A, Pedersen O, Hansen T, Molven A, Njølstad PR. Mutations in the VNTR of the carboxyl-ester lipase gene (CEL) are a rare cause of monogenic diabetes. Hum Genet 2010;127:55-64
- Tsai FJ, Yang CF, Chen CC, Chuang LM, Lu CH, Chang CT, Wang TY, Chen RH, Shiu CF, Liu YM, Chang CC, Chen P, Chen CH, Fann CS, Chen YT, Wu JY. A genome-wide association study identifies susceptibility variants for type 2 diabetes in Han Chinese. PLoS Genet 2010;6:e1000847
- Unoki H, Takahashi A, Kawaguchi T, Hara K, Horikoshi M, Andersen G, Ng DP, Holmkvist J, Borch-Johnsen K, Jørgensen T, Sandbaek A, Lauritzen T, Hansen T, Nurbaya S, Tsunoda T, Kubo M, Babazono T, Hirose H, Hayashi M, Iwamoto Y, Kashiwagi A, Kaku K, Kawamori R, Tai ES, Pedersen O, Kamatani N, Kadowaki T, Kikkawa R, Nakamura Y, Maeda S. SNPs in KCNQ1 are associated with susceptibility to type 2 diabetes in East Asian and European populations. Nat Genet 2008;40:1098-1102
- Voight BF, Scott LJ, Steinthorsdottir V, Morris AP, Dina C, Welch RP, Zeggini E, Huth C, Aulchenko YS, Thorleifsson G, McCulloch LJ, Ferreira T, Grallert H, Amin N, Wu G, Willer CJ, Raychaudhuri S, McCarroll SA, Langenberg C, Hofmann OM, Dupuis J, Qi L, Segrè AV, van Hoek M, Navarro P, Ardlie K, Balkau B, Benediktsson R, Bennett AJ, Blagieva R, Boerwinkle E, Bonnycastle LL, Bengtsson Boström K, Bravenboer B, Bumpstead S, Burtt NP, Charpentier G, Chines PS, Cornelis M, Couper DJ, Crawford G, Doney AS, Elliott KS, Elliott AL, Erdos MR, Fox CS, Franklin CS, Ganser M, Gieger C, Grarup N, Green T, Griffin S, Groves CJ, Guiducci C, Hadjadj S, Hassanali N, Herder C, Isomaa B, Jackson AU, Johnson PR, Jørgensen T, Kao WH, Klopp N, Kong A, Kraft P, Kuusisto J, Lauritzen T, Li M, Lieverse A, Lindgren CM, Lyssenko V, Marre M, Meitinger T, Midthjell K, Morken MA, Narisu N, Nilsson P, Owen KR, Payne F, Perry JR, Petersen AK, Platou C, Proença C, Prokopenko I, Rathmann W, Rayner NW, Robertson NR, Rocheleau G, Roden M, Sampson MJ, Saxena R, Shields BM, Shrader P, Sigurdsson G, Sparsø T, Strassburger K, Stringham HM, Sun Q, Swift AJ, Thorand B, Tichet J, Tuomi T, van Dam RM, van Haeften TW, van Herpt T, van Vliet-Ostaptchouk JV, Walters GB, Weedon MN, Wijmenga C, Witteman J, Bergman RN, Cauchi S, Collins FS, Gloyn AL, Gyllensten U, Hansen T, Hide WA, Hitman GA, Hofman A, Hunter DJ, Hveem K, Laakso M, Mohlke KL, Morris AD, Palmer CN, Pramstaller PP, Rudan I, Sijbrands E, Stein LD, Tuomilehto J, Uitterlinden A, Walker M, Wareham NJ, Watanabe RM, Abecasis GR, Boehm BO, Campbell H, Daly MJ, Hattersley AT, Hu FB, Meigs JB, Pankow JS, Pedersen O, Wichmann HE, Barroso I, Florez JC, Frayling TM, Groop L, Sladek R, Thorsteinsdottir U, Wilson JF, Illig T, Froguel P, van Duijn CM, Stefansson K, Altshuler D, Boehnke M, McCarthy MI; MAGIC investigators; GIANT Consortium. Twelve type 2 diabetes susceptibility loci identified through large-scale association analysis. Nat Genet 2010;42:579-589
- Waeber G, Delplanque J, Bonny C, Mooser V, Steinmann M, Widmann C, Maillard A, Miklossy J, Dina C, Hani EH, Vionnet N, Nicod P, Boutin P, Froguel P. The gene MAPK8IP1, encoding islet-brain-1, is a candidate for type 2 diabetes. Nat Genet 2000;24:291-295
- Wang H, Rissanen J, Miettinen R, Kärkkäinen P, Kekäläinen P, Kuusisto J, Mykkänen L, Karhapää P, Laakso M. New amino acid substitutions in the IRS-2 gene in Finnish and Chinese subjects with late-onset type 2 diabetes. Diabetes 2001;50:1949-1951
- Wegner L, Andersen G, Sparsø T, Grarup N, Glümer C, Borch-Johnsen K, Jørgensen T, Hansen T, Pedersen O. Common variation in LMNA increases susceptibility to type 2 diabetes and associates with elevated fasting glycemia and estimates of body fat and height in the general population: studies of 7,495 Danish whites. Diabetes 2007;56:694-698
- Wellcome Trust Case Control Consortium, Craddock N, Hurles ME, Cardin N, Pearson RD, Plagnol V, Robson S, Vukcevic D, Barnes C, Conrad DF, Giannoulatou E, Holmes C, Marchini JL, Stirrups K, Tobin MD, Wain LV, Yau C, Aerts J, Ahmad T, Andrews TD, Arbury H, Attwood A, Auton A, Ball SG, Balmforth AJ, Barrett JC, Barroso I, Barton A, Bennett AJ, Bhaskar S, Blaszczyk K, Bowes J, Brand OJ, Braund PS, Bredin F, Breen G, Brown MJ, Bruce IN, Bull J, Burren OS, Burton J, Byrnes J, Caesar S, Clee CM, Coffey AJ, Connell JM, Cooper JD, Dominiczak AF, Downes K, Drummond HE, Dudakia D, Dunham A, Ebbs B, Eccles D, Edkins S, Edwards C, Elliot A, Emery P, Evans DM, Evans G, Eyre S, Farmer A, Ferrier IN, Feuk L, Fitzgerald T, Flynn E, Forbes A, Forty L, Franklyn JA, Freathy RM, Gibbs P, Gilbert P, Gokumen O, Gordon-Smith K, Gray E, Green E, Groves CJ, Grozeva D, Gwilliam R, Hall A, Hammond N, Hardy M, Harrison P, Hassanali N, Hebaishi H, Hines S, Hinks A, Hitman GA, Hocking L, Howard E, Howard P, Howson JM, Hughes D, Hunt S, Isaacs JD, Jain M, Jewell DP, Johnson T, Jolley JD, Jones IR, Jones LA, Kirov G, Langford CF, Lango-Allen H, Lathrop GM, Lee J, Lee KL, Lees C, Lewis K, Lindgren CM, Maisuria-Armer M, Maller J, Mansfield J, Martin P, Massey DC, McArdle WL, McGuffin P, McLay KE, Mentzer A, Mimmack ML, Morgan AE, Morris AP, Mowat C, Myers S, Newman W, Nimmo ER, O'Donovan MC, Onipinla A, Onyiah I, Ovington NR, Owen MJ, Palin K, Parnell K, Pernet D, Perry JR, Phillips A, Pinto D, Prescott NJ, Prokopenko I, Quail MA, Rafelt S, Rayner NW, Redon R, Reid DM, Renwick, Ring SM, Robertson N, Russell E, St Clair D, Sambrook JG, Sanderson JD, Schuilenburg H, Scott CE, Scott R, Seal S, Shaw-Hawkins S, Shields BM, Simmonds MJ, Smyth DJ, Somaskantharajah E, Spanova K, Steer S, Stephens J, Stevens HE, Stone MA, Su Z, Symmons DP, Thompson JR, Thomson W, Travers ME, Turnbull C, Valsesia A, Walker M, Walker NM, Wallace C, Warren-Perry M, Watkins NA, Webster J, Weedon MN, Wilson AG, Woodburn M, Wordsworth BP, Young AH, Zeggini E, Carter NP, Frayling TM, Lee C, McVean G, Munroe PB, Palotie A, Sawcer SJ, Scherer SW, Strachan DP, Tyler-Smith C, Brown MA, Burton PR, Caulfield MJ, Compston A, Farrall M, Gough SC, Hall AS, Hattersley AT, Hill AV, Mathew CG, Pembrey M, Satsangi J, Stratton MR, Worthington J, Deloukas P, Duncanson A, Kwiatkowski DP, McCarthy MI, Ouwehand W, Parkes M, Rahman N, Todd JA, Samani NJ, Donnelly P. Genome-wide association study of CNVs in 16,000 cases of eight common diseases and 3,000 shared controls. Nature 2010;464:713-720
- Winckler W, Weedon MN, Graham RR, McCarroll SA, Purcell S, Almgren P, Tuomi T, Gaudet D, Boström KB, Walker M, Hitman G, Hattersley AT, McCarthy MI, Ardlie KG, Hirschhorn JN, Daly MJ, Frayling TM, Groop L, Altshuler D. Evaluation of common variants in the six known maturity-onset diabetes of the young (MODY) genes for association with type 2 diabetes. Diabetes 2007;56:685-693
- Xi B, Takeuchi F, Chandak GR, Kato N, Pan HW; AGEN-T2D Consortium, Zhou DH, Pan HY, Mi J. Common polymorphism near the MC4R gene is associated with type 2 diabetes: data from a meta-analysis of 123,373 individuals. Diabetologia 2012;55:2660-2666
- Yamauchi T, Hara K, Maeda S, Yasuda K, Takahashi A, Horikoshi M, Nakamura M, Fujita H, Grarup N, Cauchi S, Ng DP, Ma RC, Tsunoda T, Kubo M, Watada H, Maegawa H, Okada-Iwabu M, Iwabu M, Shojima N, Shin HD, Andersen G, Witte DR, Jørgensen T, Lauritzen T, Sandbæk A, Hansen T, Ohshige T, Omori S, Saito I, Kaku K, Hirose H, So WY, Beury D, Chan JC, Park KS, Tai ES, Ito C, Tanaka Y, Kashiwagi A, Kawamori R, Kasuga M, Froguel P, Pedersen O, Kamatani N, Nakamura Y, Kadowaki T. A genome-wide association study in the Japanese population identifies susceptibility loci for type 2 diabetes at UBE2E2 and C2CD4A-C2CD4B. Nat Genet 2010;42:864-868
- Yasuda K, Miyake K, Horikawa Y, Hara K, Osawa H, Furuta H, Hirota Y, Mori H, Jonsson A, Sato Y, Yamagata K, Hinokio Y, Wang HY, Tanahashi T, Nakamura N, Oka Y, Iwasaki N, Iwamoto Y, Yamada Y, Seino Y, Maegawa H, Kashiwagi A, Takeda J, Maeda E, Shin HD, Cho YM, Park KS, Lee HK, Ng MC, Ma RC, So WY, Chan JC, Lyssenko V, Tuomi T, Nilsson P, Groop L, Kamatani N, Sekine A, Nakamura Y, Yamamoto K, Yoshida T, Tokunaga K, Itakura M, Makino H, Nanjo K, Kadowaki T, Kasuga M. Variants in KCNQ1 are associated with susceptibility to type 2 diabetes mellitus. Nat Genet 2008;40:1092-1097
- Zeggini E, Scott LJ, Saxena R, Voight BF, Marchini JL, Hu T, de Bakker PI, Abecasis GR, Almgren P, Andersen G, Ardlie K, Bostršm KB, Bergman RN, Bonnycastle LL, Borch-Johnsen K, Burtt NP, Chen H, Chines PS, Daly MJ, Deodhar P, Ding CJ, Doney AS, Duren WL, Elliott KS, Erdos MR, Frayling TM, Freathy RM, Gianniny L, Grallert H, Grarup N, Groves CJ, Guiducci C, Hansen T, Herder C, Hitman GA, Hughes TE, Isomaa B, Jackson AU, J¿rgensen T, Kong A, Kubalanza K, Kuruvilla FG, Kuusisto J, Langenberg C, Lango H, Lauritzen T, Li Y, Lindgren CM, Lyssenko V, Marvelle AF, Meisinger C, Midthjell K, Mohlke KL, Morken MA, Morris AD, Narisu N, Nilsson P, Owen KR, Palmer CN, Payne F, Perry JR, Pettersen E, Platou C, Prokopenko I, Qi L, Qin L, Rayner NW, Rees M, Roix JJ, Sandbaek A, Shields B, Sjšgren M, Steinthorsdottir V, Stringham HM, Swift AJ, Thorleifsson G, Thorsteinsdottir U, Timpson NJ, Tuomi T, Tuomilehto J, Walker M, Watanabe RM, Weedon MN, Willer CJ; Wellcome Trust Case Control Consortium, Illig T, Hveem K, Hu FB, Laakso M, Stefansson K, Pedersen O, Wareham NJ, Barroso I, Hattersley AT, Collins FS, Groop L, McCarthy MI, Boehnke M, Altshuler D. Meta-analysis of genome-wide association data and large-scale replication identifies additional susceptibility loci for type 2 diabetes. Nat Genet 2008;40:638-645
- Zeggini E, Weedon MN, Lindgren CM, Frayling TM, Elliott KS, Lango H, Timpson NJ, Perry JR, Rayner NW, Freathy RM, Barrett JC, Shields B, Morris AP, Ellard S, Groves CJ, Harries LW, Marchini JL, Owen KR, Knight B, Cardon LR, Walker M, Hitman GA, Morris AD, Doney AS; Wellcome Trust Case Control Consortium (WTCCC), McCarthy MI, Hattersley AT. Replication of genome-wide association signals in UK samples reveals risk loci for type 2 diabetes. Science 2007;316:1336-1341 [Erratum in Science 2007;317:1035-1036]
